# Exercise Modalities, Training Dose, and Circulating Myostatin and Follistatin in Adults: A Systematic Review and Multilevel Meta-Analysis

**DOI:** 10.3390/ijms27219657

**Published:** 2026-10-28

**Authors:** Chuyuan Qiao, Chuwei Yang, Sitong Guo, Shenglei Yang, Yan Wang

**Affiliations:** 1 School of Sports Medicine and Rehabilitation, Beijing Sport University, Beijing 100084, China; xiaosadashuai@163.com (C.Q.); chuizigou@163.com (C.Y.); y17616251939@163.com (S.Y.); 2 National Institute of Biological Sciences, Beijing 102206, China; guositong@nibs.ac.cn

**Keywords:** myostatin, follistatin, structured exercise, skeletal muscle, exerkines, multilevel meta-analysis

## Abstract

Exercise-associated changes in circulating myostatin (MSTN) and follistatin (FST) across training modalities remain uncertain. Seven bibliographic databases were searched for controlled exercise interventions lasting at least four weeks in adults (PROSPERO CRD420261492548). Multilevel random-effects models used study-clustered CR2 inference. Renewed retrieval enabled assessment of 203 of 220 reports. The review included 79 reports from 78 study families; 39 studies contributed primary effects. Exercise was associated with lower MSTN (57 effects; 33 studies; Hedges’ *g* = 1.25, 95% CI 0.68–1.82) and higher FST (42 effects; 23 studies; *g* = 1.58, 95% CI 0.92–2.23). Heterogeneity remained high and reference-model prediction intervals crossed zero. Excluding extreme effects attenuated the pooled magnitudes. Graph-exclusion and leave-one-study-out analyses retained positive average-effect intervals. Exploratory modality and dose analyses did not establish superiority or an optimal prescription. GRADE certainty was low for both outcomes. Exercise may alter circulating MSTN and FST, but assay differences, retrieval restrictions and heterogeneous protocols limit interpretation. These findings do not establish a confirmed intracellular mechanism or validated clinical surrogate endpoint.

## 1. Introduction

The maintenance of skeletal muscle mass and function depends on a continuously regulated balance among mechanical loading, energy metabolism, and growth-factor signaling. Myostatin (MSTN; also known as growth differentiation factor 8), a member of the transforming growth factor-β superfamily, is a major negative regulator of skeletal muscle growth; deletion of the mouse *Mstn* gene causes marked myofiber hyperplasia and hypertrophy [1]. After binding to ActRIIB, mature MSTN promotes the assembly of a heteromeric receptor complex in which the type II receptor recruits and activates ALK4/5. Activated ALK4/5 receptors phosphorylate SMAD2/3, which associates with the common-mediator SMAD4 and translocates to the nucleus to regulate transcription, thereby constraining myogenesis and anabolic processes [2,3,4]. Follistatin (FST) binds MSTN, activin A, and related ActRIIB ligands in the extracellular space, reducing their availability for receptor binding and acting as a key antagonist of this inhibitory axis [2]. Circulating FST, however, does not simply reflect local release from skeletal muscle. Human arterial–hepatic venous sampling demonstrated net splanchnic FST release at rest and during exercise, supporting a hepatic contribution to circulating FST; the same study implicated regulation by the glucagon-to-insulin ratio [5]. Thus, circulating MSTN and FST may inform the study of exercise-related molecular adaptation, but both are influenced by multiple tissue sources and the systemic metabolic milieu.

Structured exercise provides a biologically plausible, nonpharmacological stimulus for modulating the MSTN–FST axis. Human studies and reviews suggest that endurance and resistance training may alter MSTN expression or circulating concentrations, potentially contributing to muscular and metabolic adaptation [6]. Acute experiments further indicate that different exercise modalities can produce transient changes in circulating FST and related activin ligands, while baseline concentrations vary with age, adiposity, and metabolic status [7]. Circulating FST responses are also shaped by hepatic endocrine signaling [5]. A recent review of exercise intensity and circulating exerkines found inconsistent MSTN and FST responses across acute and longitudinal studies, with evidence on dose and intensity remaining largely descriptive and hypothesis generating [8]. Collectively, these findings suggest that observed changes in MSTN or FST may depend on exercise modality, training dose, program duration, participant metabolic status, the interval between the final exercise session and sampling, and the assay platform. No single training modality or biomarker is therefore likely to capture the full long-term adaptation of this molecular axis.

Existing evidence has not addressed these issues simultaneously. A 2023 systematic review and meta-analysis reported that resistance training was associated with lower circulating MSTN and higher FST in adults, but its scope was restricted to resistance training [9]. A meta-analysis of exerkines in people with type 2 diabetes included diverse exercise protocols and molecular markers, yet focused on the broader exerkine profile and metabolic control rather than an evidence framework centered on the MSTN–FST axis [10]. A 2026 multilevel meta-analysis explicitly addressed within-study effect-size dependence but examined only resistance training and serum MSTN in frail or sarcopenic older adults [11]. Another meta-analysis, focused primarily on circulating irisin in adults with overweight or obesity, included MSTN and FST only as secondary outcomes and did not conduct an independent systematic search for these markers [12]. Although these studies advanced specific aspects of the field, an integrated evaluation covering healthy and clinically at-risk adults, multiple long-term structured exercise modalities, correlated within-study effects, and training-dose information remains lacking. Multiple intervention arms, shared controls, and multiple eligible comparisons can generate dependent effect sizes; ignoring this structure may underestimate uncertainty. Whether exercise modality and training dose modify the molecular response also remains unresolved.

To address these gaps, we conducted a systematic review, multilevel meta-analysis, and exploratory dose–response analysis of structured exercise programs lasting at least 4 weeks in adults. Circulating MSTN and FST were the primary quantitative outcomes; skeletal muscle protein and mRNA expression were analyzed separately by measurement level. Multilevel random-effects models with study-clustered robust correction were used to account for correlated effects within studies. Where permitted by the evidence, we explored resistance training, aerobic or concurrent training, and high-intensity functional training, together with program duration and other extractable dose characteristics. These analyses were intended to identify potential effect modifiers and evidence gaps, not to rank exercise modalities or define an unvalidated optimal prescription.

## 2. Results

### 2.1. Literature Search and Screening

**Figure 1:**
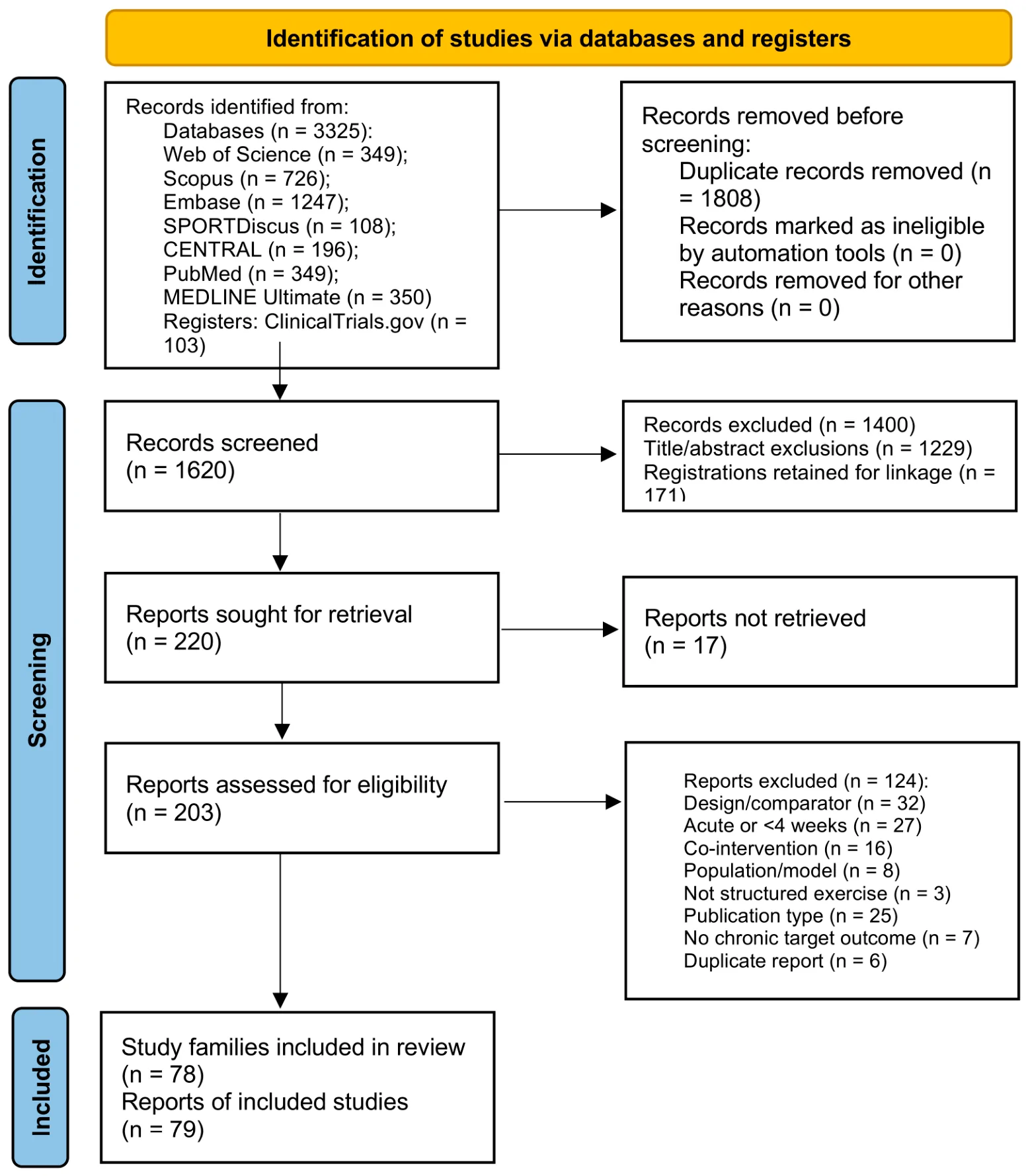
PRISMA 2020 flow diagram. Searches yielded 3428 records; 1808 duplicates were removed. Of 1620 unique records, 1229 were excluded by title/abstract and 171 registry records were retained separately for linkage. Of 220 sought reports, 203 underwent eligibility assessment and 17 remained unavailable. A total of 124 retrieved reports were excluded; 79 reports represented 78 independent study families. Report overlap identified after retrieval was handled at full-text assessment, without changing the historical deduplication count.

After historical deduplication, 1620 records were screened; 1229 were excluded by title/abstract and 171 registry records were retained for linkage. Of 220 sought reports, 203 underwent eligibility assessment and 17 remained unavailable. A total of 124 retrieved reports were excluded; 79 reports represented 78 independent study families (Figure 1; Supplementary Table S1). Eligibility reassessment excluded four previously included reports (reasons in Supplementary Table S1): Ryan, AS (2013) [13]; Jerobin, J. (2021) [14]; Micielska K (2021) [15]; Park J (2021) [16].

Registration identifiers were reported for 15 previously verified studies. Registration metadata for recovered reports are retained separately and have not been assigned retrospective concordance without verification. A bounded revision-stage linkage audit identified exact registry-ID matches to two included studies within the 171-record queue. Current ClinicalTrials.gov outcome lists were retrievable for seven included studies: one listed muscle myostatin expression, which does not establish concordance with circulating measurements, and six did not explicitly list the target biomarkers. Historical registry versions and the remaining non-ClinicalTrials records were not adjudicated, so neither the number of unpublished trials nor prospective outcome concordance could be established (Supplementary Table S11 and Supplementary File S2).

### 2.2. Characteristics of Included Studies

Reports published from 2004 to 2026 encompassed resistance, aerobic/endurance, concurrent, HIFT/CrossFit, and other structured exercise, including multicomponent training and Pilates. Thirty-nine independent study families supplied compatible primary circulating effects. Other eligible reports contributed active-training comparisons, tissue or ratio outcomes, or findings without compatible quantitative summaries. Source-specific denominators, assays, and sampling caveats are retained in Table 1, Tables S2 and S3.

### 2.3. Main Results

#### 2.3.1. Circulating Myostatin

**Figure 2:**
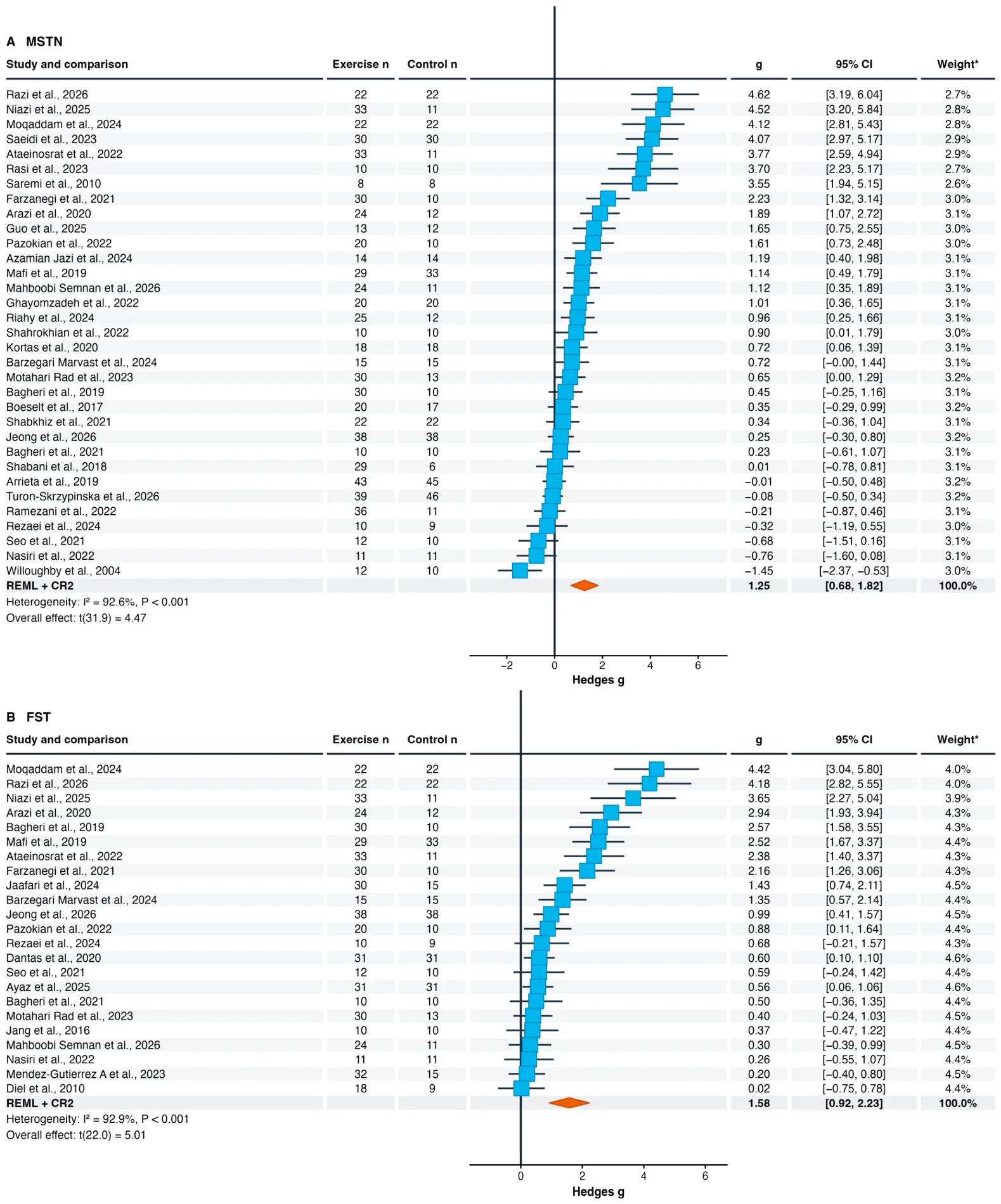
Circulating MSTN (**A**) and FST (**B**). For readability, squares and horizontal lines display study-level summaries and 95% CIs obtained by within-study aggregation under a compound-symmetry correlation of 0.5. All 99 comparison effects remain in the primary multilevel model and Supplementary Table S3A. Diamonds show the primary multilevel REML estimates with study-clustered CR2 inference, not a model refitted to the displayed summaries. Exercise/control *n* denotes distinct reported arms within each study; shared controls are not summed repeatedly. Symbol sizes and Weight* are descriptive working-independence display weights, not CR2 inferential weights. Positive *g* denotes lower MSTN or higher FST and does not establish benefit. CI, confidence interval; CR2, cluster-robust small-sample covariance correction; FST, follistatin; MSTN, myostatin; REML, restricted maximum likelihood. The studies contributing to the two panels are reported in [17,19,21,22,24,25,26,27,28,30,31,34,36,39,44,45,48,49,50,51,52,54,55,57,59,60,62,69,75,78,80,83,84,85,90,92,93,94,95].

The primary MSTN analysis included 57 effects from 33 independent studies. Multilevel REML with study-clustered CR2 inference associated exercise with lower circulating MSTN (Hedges’ *g* = 1.25, 95% CI 0.68 to 1.82, *p* < 0.001; Figure 2A and Table 2). Positive values denote lower exercise-group concentrations, not established benefit. The working-independence reference model showed *I*^2^ = 92.56% and a prediction interval from −1.74 to 4.57.

#### 2.3.2. Circulating Follistatin

The primary FST analysis included 42 effects from 23 independent studies. Multilevel REML with study-clustered CR2 inference associated exercise with higher circulating FST (Hedges’ *g* = 1.58, 95% CI 0.92 to 2.23, *p* < 0.001; Figure 2B and Table 2). The working-independence reference model showed *I*^2^ = 92.89% and a prediction interval from −1.46 to 5.35.

#### 2.3.3. Risk of Bias, Certainty of Evidence, and Reporting Bias

Risk-of-bias assessments covered 78 study families: 73 assessed with RoB 2 and five with ROBINS-I. RoB 2 judgments were Some concerns for 70 families and High for three; all five ROBINS-I judgments were Serious (Table S8A,B; Figures S1 and S2). CQ and CY reviewed the domain-level judgments. Assessments were outcome-specific: for example, laboratory-based MSTN/IGF-I and ultrasound-dependent MSTN/CSA were distinguished. Both circulating outcomes had Low GRADE certainty after one-level downgrades for risk of bias and inconsistency (Table S7).

Formal small-study-effect tests were not performed. Neither the larger evidence set nor the absence of a formal asymmetry test establishes absence of publication or availability bias; these risks remain relevant to certainty.

#### 2.3.4. Sensitivity and Influence Analyses

Sixty-three of 99 effects used graph-derived summaries. Excluding these gave MSTN *g* = 0.94 (95% CI 0.22 to 1.66) and FST *g* = 1.31 (95% CI 0.41 to 2.22). These intervals did not cross zero, but retained fewer studies and do not remove extraction or availability concerns (Supplementary Table S4).

Excluding effects with |*g*| > 3 attenuated the estimates to MSTN *g* = 0.56 (95% CI 0.22 to 0.90) and FST *g* = 1.00 (95% CI 0.62 to 1.37). Between-study variance in the primary multilevel models fell from 2.4053 to 0.5546 for MSTN and from 2.1005 to 0.5061 for FST; working-independence reference-model *I*^2^ fell from 92.56% to 78.36% and from 92.89% to 73.40%, respectively. All 56 leave-one-study-out refits using the primary multilevel CR2 model retained intervals above zero (Supplementary Table S4). This does not establish homogeneous effects across settings.

#### 2.3.5. Exploratory Subgroup and Dose–Response Analyses

All modality analyses were post hoc and exploratory. Circuit resistance training was classified as resistance based; Pilates was classified as Other structured exercise. One-study subgroups were not pooled. Nominal omnibus statistics were MSTN F(3, 1.95) = 36.24, *p* = 0.029 and FST F(3, 2.33) = 5130.34, *p* < 0.001. Sparse categories and low effective degrees of freedom make these diagnostic rather than confirmatory comparisons; no modality ranking follows (Table S5A–C).

**Figure 3:**
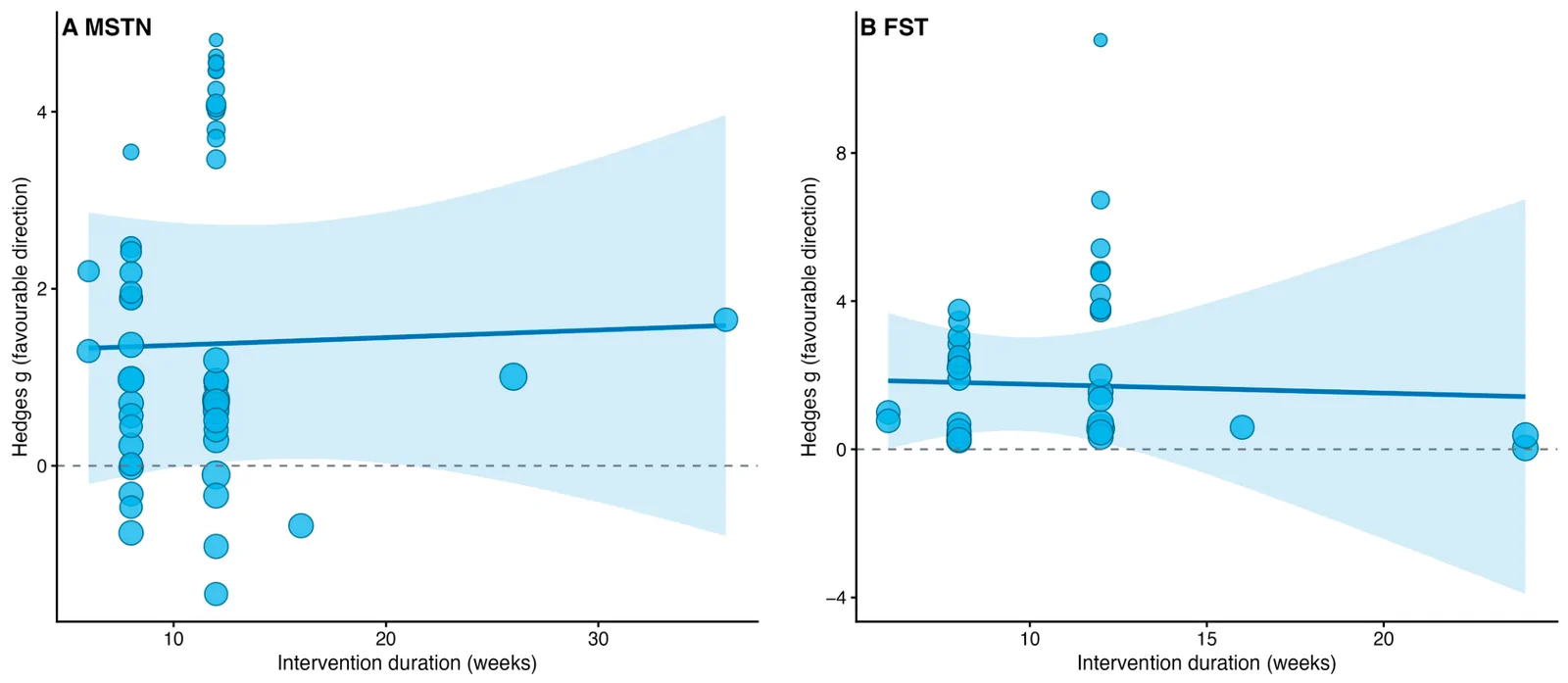
Exploratory linear dose–response associations between program duration and effect sizes for circulating myostatin and follistatin. (**A**) Myostatin (MSTN). (**B**) Follistatin (FST). Solid lines show multilevel meta-regression predictions, shaded bands show 95% confidence intervals, and circles represent eligible comparisons scaled by effect-size precision. Horizontal dashed lines mark the null. Neither linear slope was statistically significant. Given the limited number of studies, effective degrees of freedom, and dose variation, these analyses are exploratory and do not identify an optimal program duration or nonlinear dose threshold.

Per four-week increase in duration, the change in *g* was 0.03 for MSTN (95% CI −0.33 to 0.40, *p* = 0.717; 30 studies) and −0.09 for FST (95% CI −1.54 to 1.35, *p* = 0.833; 20 studies; Figure 3). Per 12 sessions, slopes were 0.03 for MSTN (95% CI −0.30 to 0.37, *p* = 0.707; 30 studies) and −0.09 for FST (95% CI −1.50 to 1.33, *p* = 0.851; 19 studies). The duration reported by Jaafari et al. [93] was excluded because the source reports both eight and twelve weeks. Low effective degrees of freedom and uneven coverage preclude an optimal-dose inference (Table S6A,B).

#### 2.3.6. Secondary Muscle and Ratio Outcomes

Supplementary Table S9 contains 36 descriptive report entries; 19 include muscle-level measurements, and six report circulating FST:MSTN ratios, with overlap between categories. Muscle MSTN findings included reductions and unchanged responses, while some reports described increased muscle FST expression. Molecular level, sampling, and comparator differences precluded a common secondary SMD. The Watson report contributes an independent active-training study, not a primary exercise-versus-nonexercise contrast [86]. de Souza contributes muscle *MSTN* mRNA; FSTL3 is not canonical FST [88]. Hooshmandi reports MSTN/IGF-I and MSTN/CSA ratios, which were not converted to absolute MSTN concentrations [91]. The MSTN:FST ratio reported by Bijeh et al. [82] was kept distinct from FST:MSTN.

## 3. Discussion

### 3.1. Key Findings and Comparison with Previous Evidence

The enlarged evidence set associated exercise with lower circulating MSTN and higher FST. The current average estimates were 1.25 and 1.58; removing extreme effects attenuated their magnitudes. Graph-exclusion and primary-model influence analyses retained positive average-effect intervals. Nevertheless, high heterogeneity and reference prediction intervals crossing zero preclude treating standardized magnitudes as fixed across settings or as evidence of a confirmed intracellular pathway.

These findings extend the scope and methods of the 2023 meta-analysis by Khalafi et al., which reported lower circulating MSTN and higher FST after resistance training [9]. The present synthesis broadens modality coverage and handles multiple effects from each study using multilevel models with study-clustered CR2 inference. It also separates available dose information from dimensions that could not be modeled. These changes address evidence structure rather than establish causal superiority of any modality. Differences in study populations, assays, sampling schedules, and statistical estimators mean that standardized magnitudes should not be directly compared across reviews.

### 3.2. Exercise Modality and Training Dose

Resistance-based training contributed most evidence. The HIFT/CrossFit evidence remained sparse, and the FST interval crossed zero. The expanded aerobic/concurrent FST group contained six studies, while multicomponent exercise and Pilates were kept in an Other structured exercise category. Categorical models reduced in-sample between-study variance, but low effective degrees of freedom, post hoc definitions and study-level confounding prevent confirmatory modality comparisons. The binary model also did not establish a difference. Broader exerkine research likewise suggests that aerobic and resistance exercise can alter circulating factors, with magnitude and time course depending on the molecule and sampling schedule [96]. Adequately powered, assay-standardized, head-to-head trials are needed to determine whether modality truly modifies the response.

The available study-level evidence did not resolve linear associations with duration or session count. Although the recovered reports broadened the duration range, informative dose levels remained uneven and the CR2 slope degrees of freedom were low. Calendar months without exact weeks or sessions were not converted to artificial dose values. Duration and session count do not capture intensity, volume, progression, adherence, or energy expenditure, and their separate regressions do not estimate independent causal effects. Future trials should report these components consistently and vary a defined component while holding others comparable.

### 3.3. Molecular Interpretation and Assay Context

**Figure 4:**
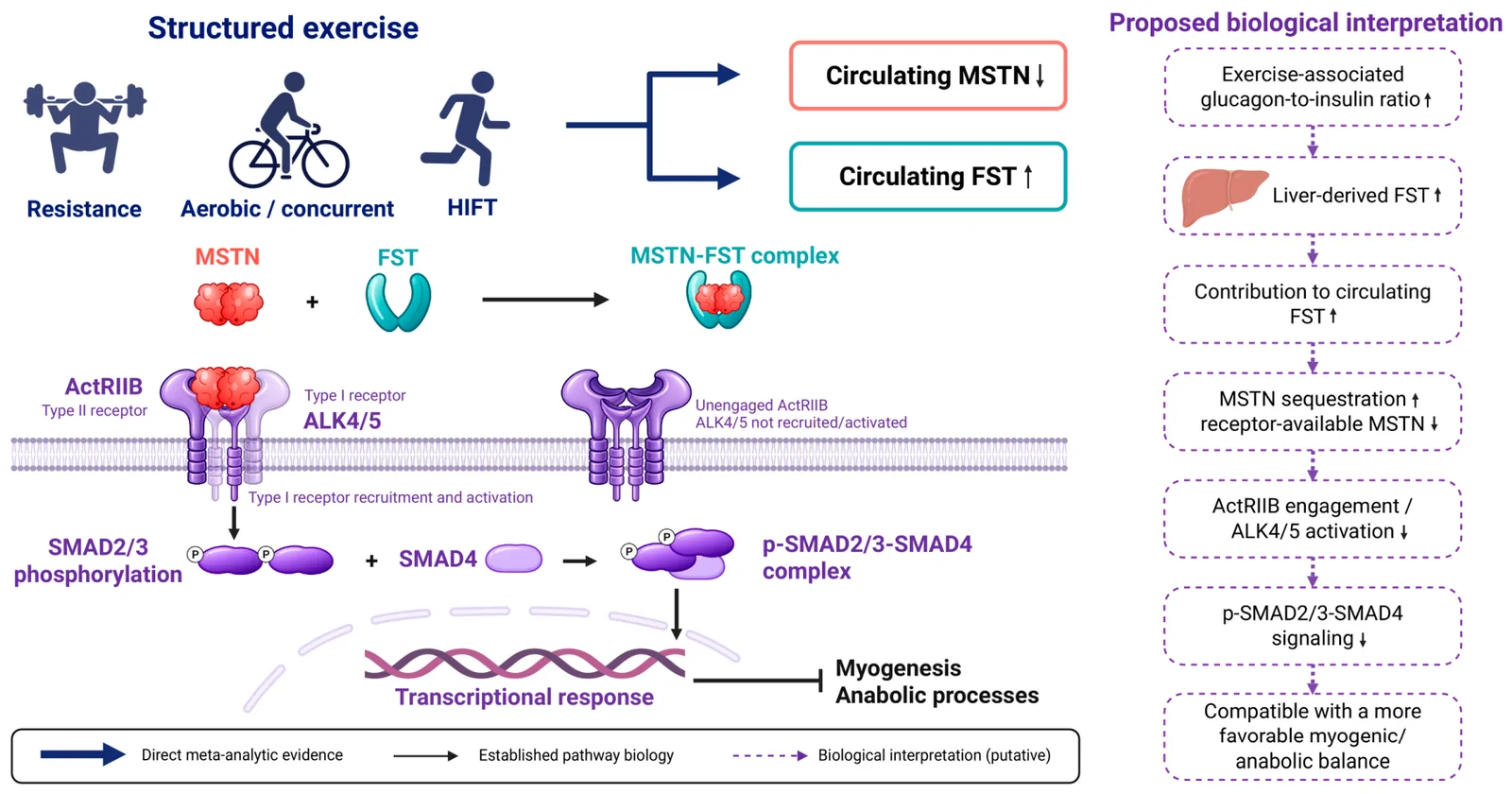
Evidence-informed model of exercise-associated modulation of the myostatin–follistatin axis. Across the included structured-exercise interventions, the pooled evidence indicated lower circulating myostatin (MSTN) and higher circulating follistatin (FST). Thick navy arrows denote associations directly synthesized in this meta-analysis. Thin black arrows show established pathway biology that was not directly tested in the included studies. Red and teal denote MSTN and FST, respectively; purple receptor and SMAD illustrations depict pathway components. Upward and downward arrows indicate increases and decreases, respectively; circled P denotes phosphorylation, and T-shaped bars denote inhibition. FST sequesters MSTN and reduces its availability for receptor binding [2]. Receptor-available MSTN binds ActRIIB, promotes ALK4/5 activation and SMAD2/3 phosphorylation, and facilitates formation of the p-SMAD2/3–SMAD4 complex involved in transcriptional regulation of myogenesis and anabolic processes [2,3,4,97]. Purple dashed elements indicate a proposed interpretation in which an exercise-associated increase in the glucagon-to-insulin ratio may promote hepatic FST secretion and attenuate downstream MSTN signaling [5]. The entire proposed sequence in the right-hand column, including downstream myogenic or anabolic adaptation, was not measured as a mediating pathway in this review. Circulating concentrations do not establish ligand bioactivity, receptor occupancy, intracellular signaling, or muscle adaptation. Given the high heterogeneity and prediction intervals crossing the null, the model does not imply a superior exercise modality, an optimal dose, a confirmed mechanism, or a validated clinical surrogate endpoint. Abbreviations: ActRIIB, activin receptor type IIB; ALK4/5, activin receptor-like kinases 4 and 5; FST, follistatin; HIFT, high-intensity functional training; MSTN, myostatin; p-SMAD2/3, phosphorylated SMAD2/3; SMAD4, SMAD family member 4.

The observed effect directions are compatible with established regulation of skeletal muscle growth, but this meta-analysis does not show that the pathway mediates exercise adaptation. MSTN is a negative regulator of skeletal muscle mass [1]. After binding to ActRIIB, receptor-available MSTN promotes recruitment and activation of ALK4/5, followed by phosphorylation of SMAD2/3. Phosphorylated SMAD2/3 associates with SMAD4, and the resulting complex translocates to the nucleus to regulate transcriptional responses that suppress myogenesis and anabolic signaling [2,3,4,97]. FST binds and neutralizes MSTN and activin, reducing ligand availability for activin-receptor binding [2,7]. Lower circulating MSTN together with higher FST after exercise is therefore compatible with reduced availability of inhibitory ligands, and the opposing directions may be more informative than either marker alone. Circulating concentration, however, is not equivalent to ligand bioactivity, receptor occupancy, ALK4/5 activation, SMAD2/3 phosphorylation, p-SMAD2/3–SMAD4 complex formation, nuclear translocation, transcriptional regulation, muscle protein synthesis, or tissue-specific signaling flux. An exercise-associated increase in circulating FST is not a muscle-specific readout. Future studies should pair blood with muscle sampling, distinguish active, latent, and protein-bound ligand forms, and concurrently measure ActRIIB/ALK4/5–SMAD2/3–SMAD4 signaling, myogenic targets, and functional adaptation (Figure 4).

Between-study differences may reflect both biological and methodological variation. Participants ranged from healthy adults and older adults to people with sarcopenia, obesity, metabolic disease, cancer, chronic kidney disease, heart failure, or HIV infection. Age, sex, adiposity, metabolic status, disease severity, and medication exposure may affect baseline concentrations; circulating activin and FST are themselves associated with metabolic and anthropometric characteristics [7]. Exercise protocols also varied in modality, intensity, duration, co-interventions, and the interval between the final session and sampling. Circulating MSTN measurement is particularly challenging because MSTN forms complexes with binding proteins, occurs in active and latent forms, and shares high homology with related ligands. Antibody cross-reactivity, sample pretreatment, and variation among commercial assays can yield substantially different concentrations [98,99,100]. These factors also explain why circulating protein, muscle protein, and muscle mRNA measurements are not interchangeable [6].

### 3.4. Research and Clinical Implications

Circulating MSTN and FST are better regarded at present as candidate biomarkers of molecular adaptation to exercise than as independent therapeutic targets or validated clinical surrogate endpoints. Expert consensus recognizes the potential value of the MSTN–FST system in studies of musculoskeletal aging while emphasizing the need for improved assays and validation against clinically relevant outcomes [101]. This distinction matters because pharmacological inhibition of MSTN signaling often increases muscle mass without consistently improving strength or physical function [3,102]. A concrete validation design would randomize adults within a single predefined clinical phenotype across centers to progressive resistance training, energy-expenditure-matched aerobic training, or nonexercise control. Circulating MSTN and a prespecified functional measure could be co-primary outcomes. Samples would be assayed centrally using one validated platform with a stated calibrator and a common analytical batch where feasible. Fasting status and the interval after the final session would be prespecified and recorded for each participant. Confounders would be specified in advance, and a biopsy subset would assess muscle MSTN/FST and *p*-SMAD2/3 alongside circulating markers. No target condition or reference standard was defined for either biomarker in this review; the estimates describe standardized between-group concentration differences, not diagnostic or prognostic performance.

### 3.5. Strengths and Limitations

This study has several strengths in evidence coverage, process control, and statistical analysis. Searches encompassed multiple databases and a trial registry, and search, screening, and full-text retrieval records were retained in accordance with PRISMA 2020 and PRISMA-S. CQ and CY reviewed eligibility, numerical inputs, and domain-level risk-of-bias judgments. Circulating MSTN and FST were synthesized separately, and multilevel models treated studies as independent clusters while examining sensitivity to assumed within-study correlations and extreme effects. Estimable and nonestimable dose dimensions were explicitly distinguished. By situating multiple structured exercise modalities within one evidence framework, this review provides a broader view of long-term exercise effects on the molecular axis than earlier resistance-training reviews.

Important limitations remain. Modalities, assays, populations, and doses were not randomized across studies; moderator signals remain vulnerable to confounding and sparse-category instability. Sixty-three of 99 effects were graph-derived approximations. Some reports used detection-limited subsets, noncommercial assays, ambiguous sampling, or imperfectly documented cointerventions. Unadjusted arm-summary SMDs do not reproduce source-adjusted analyses. Seventeen of 220 sought reports remained unavailable after renewed retrieval, leaving potential availability bias. Source inconsistencies prevented inclusion of the Jaafari et al. [93] MSTN denominator and duration. Original search omissions remain a limitation; an expanded PubMed export was not screened or incorporated, and the impact of these omissions is unknown. Cohort sex composition was extracted but not modeled because aggregate data could not separate sex effects from study-level confounding, and mixed-sex cohorts did not consistently provide sex-specific contrasts. Future trials should report sex-disaggregated outcomes.

## 4. Materials and Methods

### 4.1. Study Design and Reporting Standards

This systematic review, multilevel meta-analysis, and exploratory dose–response analysis evaluated the effects of structured exercise on the MSTN–FST axis in adults. Reporting followed PRISMA 2020, and the search process was reported in accordance with PRISMA-S [103,104]. The review was registered in PROSPERO (CRD420261492548). Revision-stage corrections to quantitative inputs and inference, and the separate narrative reporting of secondary measures specified in the Methods, are documented in the Supplementary Methods. These decisions are not retrospectively described as prespecified. Only aggregate data from publicly available studies were used; additional ethical approval and informed consent were therefore not required.

### 4.2. Eligibility Criteria and Exclusion Criteria

Eligibility criteria were defined using the PICOS framework. Population: adults aged ≥18 years, irrespective of sex or health status. Mixed-age samples were eligible only when adult data could be extracted separately or the sample was explicitly composed entirely of adults. Intervention: active, structured exercise lasting at least 4 weeks, with a repeatable and clearly defined protocol. In direct training comparisons, each arm had to meet this minimum duration; a longer-arm pre–post change did not substitute for an eligible controlled contrast. Eligible modalities included resistance, aerobic or endurance, interval, concurrent, circuit, high-intensity functional, and other active training programs with quantifiable dose characteristics. Combined interventions were eligible only when the effect of exercise could be isolated or all groups received the same nonexercise co-intervention (Supplementary File S1).

Comparator: nonexercise, usual-lifestyle, usual-care, or wait-list controls, as well as direct comparisons of exercise modalities, sequences, intensities, or doses. Groups defined only by disease status, with all groups receiving the same exercise, were not eligible exercise comparators. Outcomes: the primary outcomes were circulating MSTN and FST, with serum and plasma recorded separately. Secondary measures included skeletal muscle MSTN/FST protein or mRNA expression and reliably calculated FST/MSTN ratios. Circulating concentrations, tissue protein, and mRNA outcomes were not pooled together. Secondary outcomes were summarized narratively by measurement level, comparator, and sampling context because these features and reported scales differed; this synthesis choice was made during revision. Ratios were reported only as presented in the source study and were not inferred from separate group means for MSTN and FST.

Study design: eligible reports were original randomized controlled trials or nonrandomized longitudinal intervention studies with a contemporaneous control group that met the PICOS criteria and were available in full text. Preprints were flagged separately and evaluated against the same data-adequacy criteria as journal articles before quantitative synthesis. We excluded animal, cellular, and in vitro studies; mixed-age studies without separable adult data; single-session acute exercise studies and interventions lasting <4 weeks; observational physical-activity studies; multicomponent rehabilitation studies in which the exercise effect could not be isolated; and reviews, meta-analyses, editorials, protocols, cross-sectional studies, case reports, and conference abstracts with insufficient information.

### 4.3. Information Sources and Search Strategies

We searched PubMed, MEDLINE Ultimate (EBSCOhost), Embase (Embase.com), Web of Science Core Collection, Scopus, SPORTDiscus with Full Text (EBSCOhost), and Cochrane CENTRAL. ClinicalTrials.gov was searched to identify registered trials. Searches were completed on 22–23 August 2026, with no restrictions on publication year or language. Except for the exclusion of animal-only studies in Embase, no age or study-design filters were applied (Supplementary File S1).

Search strategies combined two concept blocks, MSTN/FST and structured exercise, and were adapted to the controlled vocabulary, field tags, and syntax of each platform. Dose terms were not required because frequency, intensity, volume, and program duration are generally reported only in full texts. A formal PRESS peer review was not documented [105]; complete platform-specific strategies are provided in Supplementary File S1.

The seven bibliographic databases yielded 3325 records, and ClinicalTrials.gov yielded 103 trial registrations. Records were exported in RIS or PubMed/NBIB format and merged. Duplicates were removed sequentially using normalized DOIs, PMIDs, and titles with publication years; fuzzy title matching was used only to support manual review. Of 3428 imported records, 1808 duplicates were removed, leaving 1620 unique records for screening. Original export files, batch details, record counts, and search audit logs were retained. The separate registry queue contained registrations indexed across sources, not only the direct ClinicalTrials.gov export. Of 103 directly exported registrations, 102 entered that queue and one was consolidated with a publication record during deduplication; 69 additional queue records were identified through CENTRAL or Embase. The 171 queue records mapped to 162 distinct registry identifiers (Supplementary Table S11 and Supplementary File S2).

### 4.4. Literature Screening

CQ and CY independently assessed the original screening and full-text evidence set and reviewed the recovered eligibility decisions and numerical inputs. Title and abstract screening prioritized sensitivity: records were retained for full-text review whenever the available information was insufficient to confirm an adult population, a structured exercise intervention lasting at least 4 weeks, a longitudinal intervention design, or an MSTN/FST outcome. Each reviewer’s original decision, date, exclusion reason, and any screening conflict were retained. Disagreements were resolved by discussion, with YW adjudicating when required.

Retrieval was sought for 220 reports. Renewed retrieval through interlibrary loan, institutional access, author contact, and publisher purchase supplied 99 research-full-text records (98 unique texts) and 22 original conference reports from the original 138-record queue; one related conference version did not replace its original journal report. Together with the previously assessed 82 reports, 203 reports underwent eligibility assessment; 17 originals remained unavailable. Downloaded version identities and the author Word-to-PDF conversion were retained. One primary reason was recorded per excluded retrieved report (Supplementary Table S1 and File S2).

### 4.5. Data Extraction and Outcome Definitions

The 79 included reports represented 78 independent study families. Review inclusion was distinguished from contribution to each quantitative synthesis (Supplementary Tables S2 and S3).

Outcome fields included specimen matrix, measurement level, assay platform and kit, sampling time point and interval after the final exercise session, unit, group sample size, mean, standard deviation (SD), standard error (SE), change score, and 95% confidence interval (CI), together with the source location and extraction method. SEs were converted using SD = SE × √*n*; where necessary, SEs were reconstructed from 95% CIs. Graphical values were obtained using a digitization procedure with source locations retained. Table S3A identifies original-extraction and revision-stage figure-derived values separately and provides assay, kit, and sampling metadata. Missing or inconsistent source details were not imputed. Target-marker baseline reporting and original model adjustment were checked separately from the arm-summary SMD; nonsignificant baseline tests were not taken as proof of balance (Supplementary Methods). Circulating proteins, muscle proteins, and muscle mRNA were coded separately. Follistatin-related protein 3 (FSTL3) was outside the predefined canonical FST outcome, which did not include FST-related proteins. ChatGPT (OpenAI; model GPT-5.6 Sol; application version 26.1013.40934) assisted preliminary screening and the location of bibliographic records and relevant source passages. CQ and CY independently extracted and cross-checked study characteristics, exercise prescriptions, comparators, biological specimens, assay methods, and outcome data against the source reports, including the recovered evidence. Unresolved source reporting is retained explicitly. Final eligibility decisions and extraction data remained the responsibility of CQ and CY; AI-assisted screening and source-location outputs were not accepted as final decisions or data.

### 4.6. Risk of Bias and Certainty of Evidence

Randomized trials were assessed with Cochrane RoB 2 [106], and nonrandomized intervention studies with ROBINS-I [107]. Tool selection followed the allocation underlying the extracted comparison, not the study label: reported random allocation was assessed with RoB 2, with unclear sequence generation or concealment judged within its domains; nonrandom allocation or a control group augmented after randomization was assessed with ROBINS-I. RoB 2 assessments targeted the effect of assignment; adherence-related exclusions and missing outcomes were considered separately. CQ and CY independently reviewed the original 33-study judgments and all domain-level judgments; disagreements were resolved by consensus or adjudication. Studies are labeled by first author and year in the risk-of-bias figures. Certainty of evidence was assessed separately for circulating MSTN and circulating FST using GRADE [108], considering risk of bias, inconsistency, indirectness, imprecision, and publication bias. Assessments concerned the direction of the mean between-group concentration difference and were based on the studies contributing to each outcome-specific synthesis. Uncertainty in effect magnitude and clinical relevance was considered separately, with explicit domain-level reasons reported (Supplementary Tables S7 and S8A,B; Supplementary Figures S1 and S2).

### 4.7. Effect Sizes and Data Conversion

Because specimen matrices, assay platforms, and measurement units differed across studies, the primary effect measure was Hedges’ *g*, the small-sample-corrected standardized mean difference. The current primary analyses comprised 94 postintervention comparisons and five change-score comparisons, the latter from Ghayomzadeh [39] and the matched-supplement contrasts of Mafi [55]. For postintervention data, Cohen’s *d* was calculated as the between-group mean difference divided by the pooled within-group SD and multiplied by the small-sample correction factor *J* to obtain Hedges’ *g*. For change-score comparisons, the between-group difference in mean post-minus-pre changes was divided by the pooled SD of individual change scores, not the baseline or postintervention SD, before the same small-sample correction was applied. Exact standardization formulae are given in the Supplementary Methods. These are not covariate-adjusted estimates. Effect direction was coded so that positive values represented lower MSTN or higher FST in the exercise group; this sign convention does not establish biological or clinical benefit. Sampling variances, SEs, and 95% CIs were derived from the corresponding sample sizes and Hedges’ *g*. Dependencies arising from shared controls, multiple intervention arms, and multiple eligible comparisons were modeled within studies rather than treating repeated use of a shared control as independent information. All eligible primary-outcome effects were retained to limit selective inclusion among multiple available effects [109].

### 4.8. Data Synthesis and Statistical Analysis

Meta-analyses were conducted separately for MSTN and FST. Primary concentration syntheses compared exercise with a concurrent nonexercise comparator, with nonexercise co-interventions matched where applicable. Direct active-training comparisons were not pooled with these contrasts and are identified separately in the study-level tables. The primary model was a multilevel random-effects model estimated by restricted maximum likelihood, with effect sizes nested within studies and the study as the clustering unit. SEs, 95% CIs, and *p* values were obtained using CR2 robust covariance correction and Satterthwaite small-sample degrees-of-freedom approximation [110,111,112]. A conventional independent-effects random-effects model was fitted as a reference to report the *Q* statistic, *I*^2^, and 95% prediction interval. Prediction intervals described the range of plausible true effects in a new study setting and the practical implications of between-study heterogeneity [112]. This reference model did not replace the primary model accounting for effect dependence.

Sensitivity to assumed within-study correlations was examined at ρ = 0, 0.5, and 0.8. Effect sizes were aggregated within studies under a compound-symmetry correlation structure, and restricted maximum likelihood random-effects models were refitted with the Knapp–Hartung correction. We also excluded effect sizes with |Hedges’ *g*| > 3 to assess the influence of a small number of extreme effects and conducted leave-one-study-out analyses. For the report with inconsistent participant-flow and outcome-table denominators, we retained the table-reported sizes and examined completer-size and study-omission sensitivities (Table S4).

Exercise-modality groups were data informed and analyzed post hoc. Operational rules were formalized during revision: explicit mixed strength/endurance HIFT or CrossFit workouts were classified as HIFT/CrossFit; otherwise, predominantly resistance-loading protocols were resistance based, and endurance or combined aerobic/resistance protocols were aerobic/concurrent. The recovered multicomponent balance/gait/strength program was retained as Other multicomponent rather than forced into these classes. Interval resistance training was not classified as HIFT solely because of its intensity or interval structure. Arm-specific classifications and primary-analysis assignments are listed in Supplementary Table S10. Subgroup effects were estimated only when a category contained at least two independent studies. For modality subgroup pools, dependent effects were first aggregated within studies assuming a correlation of 0.5, followed by REML estimation and a modified Knapp–Hartung interval with *k* − 1 degrees of freedom and the variance multiplier bounded below by 1, where *k* is the number of independent studies. This prevented underdispersion from producing spuriously narrow intervals. We also fitted an exploratory categorical multilevel meta-regression with resistance training as the reference, using CR2 robust covariance and Satterthwaite degrees of freedom. An HTZ Wald test with small-sample correction was used for the omnibus comparison. Because sparse categories produced extremely low effective degrees of freedom, this test was treated as diagnostic rather than confirmatory. A binary exploratory model contrasted conventional resistance-based training with the other modalities; the latter category does not imply absence of a resistance component. Moderator pseudo-R^2^ was calculated from the change in between-study variance; negative values were retained computationally and interpreted as 0%. Residual heterogeneity was reported. These post hoc modality analyses were exploratory and did not support reliable ranking. Robustness was further assessed by retaining only resistance training, excluding HIFT/CrossFit, and sequentially excluding each modality. For the restriction and category-exclusion sensitivity analyses, effects were aggregated at the study level under compound symmetry with ρ = 0.5, followed by a REML random-effects model with modified Knapp–Hartung correction, bounding its variance multiplier below by 1.

Candidate dose variables were training frequency, duration, session time, total sessions, and total exercise time. Coverage and between-study variation were checked before modeling. Frequency ranged from two to three sessions per week; only three MSTN studies and one FST study represented twice-weekly training, so frequency was not modeled. Duration per four weeks and sessions per 12 sessions were examined in separate exploratory linear multilevel models with CR2 correction, not as mutually adjusted causal dose effects. Calendar-month reports lacking exact week or session counts were not converted to assumed durations. Sparse and inconsistent reporting precluded session-time and nonlinear models. Tests were two-sided. Exploratory subgroup, dose, and sensitivity *p* values were not multiplicity adjusted and were not treated as independent confirmatory findings. The full fitting ledger is retained in Supplementary File S2.

Formal funnel-plot asymmetry tests were not performed. The circulating MSTN and FST sets contained 33 and 23 independent studies, respectively, with dependent comparisons and substantial clinical and analytical heterogeneity. These features complicate interpretation of small-study effects; the absence of a formal test is not evidence against publication bias.

Analyses were conducted in R version 4.6.1, primarily with metafor 5.0–1 [113] and clubSandwich 0.7.0. Data import and graphics also used readxl 1.5.0, ggplot2 4.0.3, patchwork 1.3.2, svglite 2.2.2, and ragg 1.5.2. Scripts for the primary meta-analysis, sensitivity analyses, leave-one-study-out diagnostics, subgroup analyses, dose meta-regression, and figures are supplied in Supplementary File S2 with R session information, analysis inputs, and plot-ready data. The execution ledger documents primary, sensitivity, influence and exploratory moderator refits; these execution counts are not independent confirmatory hypotheses. The aggregate extraction data, analysis inputs, R scripts, and numerical results are openly available in Zenodo at https://doi.org/10.5281/zenodo.23455332.

## 5. Conclusions

Exercise was associated with lower circulating MSTN and higher FST, but the low-certainty estimates remained heterogeneous and sensitive in magnitude. Exploratory modality and dose results do not identify a superior exercise type, optimal dose, confirmed intracellular mechanism, or validated clinical surrogate endpoint.

## Figures and Tables

**Table 1:** Characteristics of the included studies.

Study	Country	Design and Population	Baseline/ Analyzed *n*	Duration	Exercise Intervention	Comparator	Outcome, Specimen, and Assay
Ayaz et al. (2025) [17]	Turkey	Multicenter RCT; institutionalized adults aged ≥65 years	66/62	12 weeks	Combined aerobic and resistance training	Exercise vs. nonexercise control	FST; serum; immunoassay
Zhuang et al. (2025) [18]	China	Assessor-blinded RCT; adults aged ≥65 years with sarcopenia	27/27	12 weeks	Whole-body vibration or resistance training	Direct comparison of two training modalities	MSTN and FST; blood/serum; ELISA
Jeong et al. (2026) [19]	Republic of Korea	Four-arm RCT; healthy women aged ≥65 years without insulin resistance	96/76	12 weeks	Circuit resistance training	Control/RE/EAA/RE + EAA	MSTN and FST; serum; immunoassay
Zhang et al. (2024) [20]	China	Single-blind RCT; adults aged ≥65 years with sarcopenia	21/21	12 weeks	Low-load BFR resistance training or conventional high-intensity resistance training	Direct comparison of training doses/modes	MSTN and FST; serum; ELISA
Ataeinosrat et al. (2022) [21]	Iran	Four-arm RCT; adult men with obesity	44/44	12 weeks	Low-, moderate-, or high-intensity interval resistance training	Three intensities vs. nonexercise control	MSTN and FST; plasma; ELISA
Moqaddam et al. (2024) [22]	Iran	Four-arm RCT; adult men with obesity	68/44	12 weeks	CrossFit/high-intensity functional training	Control/AST/training/training + AST	MSTN and FST; plasma; ELISA
Hofmann et al. (2016) [23]	Austria	Three-arm RCT (VAAS); institutionalized older women	91/70	26 weeks	Elastic-band resistance training	RT/RT + nutrition/cognitive training control	MSTN and FST; serum; immunoassay
Seo et al. (2021) [24]	Republic of Korea	RCT; women aged ≥65 years with sarcopenia	22/22	16 weeks	Bodyweight plus elastic-band resistance training	RT vs. nonexercise control	MSTN and FST; plasma; ELISA
Saeidi et al. (2023) [25]	Iran	Four-arm RCT; adult men with obesity	68/60	12 weeks	HIFT/CrossFit	Control/AST/training/training + AST	MSTN; plasma; ELISA
Arazi et al. (2020) [26]	Iran	Three-arm RCT; untrained young women	36/36	8 weeks	Variable- or constant-resistance training	VRT/CRT/non-training control	MSTN and FST; serum; immunoassay
Mahboobi Semnan et al. (2026) [27]	Iran	Three-arm, single-blind RCT; adults who are overweight	40/35	8 weeks	Whole-body EMS or resistance training	WB-EMS/RT/non-training control	MSTN and FST; serum; ELISA
Rezaei et al. (2024) [28]	Iran	Two-arm RCT; older men with sarcopenia	19/19	8 weeks	TRX suspension training	TST vs. non-training control	MSTN and FST; serum; ELISA
Antunes-Correa et al. (2020) [29]	Brazil	Three-arm RCT; patients with NYHA Class II–III heart failure	44/33	NR	Aerobic or inspiratory muscle training	AET/IMT/control	*FST* mRNA; vastus lateralis; qPCR (Western blots concern other pathway proteins)
Dantas et al. (2020) [30]	Brazil	RCT in post-bariatric surgery patients; women who underwent Roux-en-Y gastric bypass	NR/NR	24 weeks	Combined aerobic and resistance training	RYGB + exercise/RYGB/lean reference	Circulating FST (ELISA); muscle *MSTN* transcript and FST protein (RNA profiling/Western blot)
Shahrokhian et al. (2022) [31]	Iran	Reported random allocation; two versus three arms inconsistently described; women with breast cancer	20/NR	12 weeks	Nonlinear or linear resistance training	NLRT/LRT/control	MSTN; serum; assay not specified
Binns et al. (2017) [32]	USA	Two-arm randomized longitudinal study; community-dwelling older adults	33/NR	20 weeks	High-velocity resistance training	HVRT/control	MSTN; serum; ELISA
Planella-Farrugia et al. (2019) [33]	Spain	Three-arm trial with initial random allocation and five additional controls; adults aged 60–75 years	43/43	16 weeks	Low-intensity resistance exercise	Control/RE/RE + nutrition	MSTN; serum; ELISA
Kortas et al. (2020) [34]	Poland	Two-arm RCT; postmenopausal older women	42/36	12 weeks	Nordic walking	Nordic walking/non-training control	MSTN; serum; ELISA
Zhou et al. (2021) [35]	Sweden	Parallel-group RCT; patients with non-dialysis CKD	151/NR	52 weeks	Strength or balance training	Strength/balance training	MSTN; plasma; ELISA
Guo et al. (2025) [36]	China	Two-arm randomized clinical trial; older women with sarcopenic obesity	25/25	36 weeks	Individualized resistance training	Training vs. lifestyle control	MSTN; serum; ELISA
Brooks et al. (2010) [37]	USA	Three-arm randomized controlled trial; healthy men on bed rest with an energy deficit	31/31	4 weeks	Resistance exercise during bed rest	EAA/delayed RT + EAA/pre-exercise RT + EAA	*MSTN*; vastus lateralis; qPCR
Dawson et al. (2021) [38]	USA	Randomized trial substudy; prostate cancer survivors receiving ADT	17/17	12 weeks	Periodized resistance training	RT/home-stretching control	*MSTN*; skeletal muscle biopsy; qPCR
Ghayomzadeh et al. (2022) [39]	Iran	Assessor-blinded RCT; adults living with HIV	40/37	26 weeks	Concurrent training	Concurrent training/lifestyle control	MSTN; serum; ELISA
Gould et al. (2025) [40]	UK	Randomized trial muscle biopsy substudy; patients with CKD	19/19	12 weeks	Aerobic or concurrent training	Aerobic/concurrent exercise	*MSTN* mRNA; vastus lateralis; qPCR (acute-bout sampling before/after training)
Zuo et al. (2023) [41]	China	Two-arm RCT; young men	20/18	6 weeks	Functional or traditional resistance training	FRT/TRT	MSTN; serum; ELISA
Watson et al. (2017) [42]	UK	Randomized trial biopsy substudy; patients with CKD stages 3b–4	17/17	8 weeks	Progressive resistance training	RT/non-training control	*MSTN* mRNA; vastus lateralis; qPCR (acute-bout sampling before/after training)
Radzimińska et al. (2018) [43]	Poland	Randomized double-blind controlled trial; adult women with urinary incontinence	84/74	4 weeks	Pelvic-floor muscle training	PFMT/no-treatment control	MSTN; EDTA plasma; ELISA
Razi et al. (2026) [44]	Iran	2 × 2 factorial RCT; adult men with obesity	60/44	12 weeks	HIFT/CrossFit	Placebo/supplement/HIFT/HIFT + supplement	MSTN and FST; plasma; ELISA
Turoń-Skrzypińska et al. (2026) [45]	Poland	Two-arm RCT; patients undergoing hemodialysis	NR/NR	13 weeks	Intradialytic virtual-reality exercise	NefroVR/usual dialysis	MSTN; plasma; ELISA
Brown et al. (2024) [46]	USA	2 × 2 factorial multicenter RCT; stage I–III breast or colorectal cancer	116/116	12 weeks	Aerobic exercise	Exercise/metformin/combined/control	MSTN and FST; EDTA plasma; multiplex immunoassay
Benavente et al. (2024) [47]	Spain	Three-group parallel training study; resistance-trained men	33/NR	8 weeks	Whole-body resistance training	Normoxia/terrestrial altitude/normobaric hypoxia	MSTN; serum; ELISA
Pazokian et al. (2022) [48]	Iran	Three-arm RCT; older men	30/30	6 weeks	Functional training with or without BFR	FT/FT-BFR/control	MSTN and FST; serum; ELISA
Jang et al. (2016) [49]	Republic of Korea	Two-arm RCT; male college students	20/20	8 weeks	Open- and closed-chain pulley exercises	Pulley training/non-training control	FST; serum; ELISA
Arrieta et al. (2019) [50]	Spain	RCT; older nursing-home residents	112/88	6 months	Twice-weekly multicomponent strength, balance and gait training	Usual activity	MSTN; serum; ELISA
Motahari Rad et al. (2023) [51]	Iran	Three-arm RCT; men with type 2 diabetes	51/43	12 weeks	Concurrent aerobic/resistance in opposite sequences; 3 sessions/week	Nonexercise control	MSTN and FST; serum; immunoassays
Bagheri et al. (2019) [52]	Iran	Four-arm RCT; middle-aged men	40/40	8 weeks	Upper-, lower-, or combined-body RT; 3 sessions/week	Nonexercise control	MSTN and FST; serum; ELISA
Bagheri et al. (2020) [53]	Iran	Three-arm RCT; older men with sarcopenia	30/30	8 weeks	Endurance then resistance or reverse order; 3 sessions/week	Nonexercise control	MSTN and FST; serum; immunoassays
Diel et al. (2010) [54]	Germany	Three-arm RCT; young men	33/27	3 months	Resistance or endurance; 3 sessions/week	Nonexercise control	Canonical FST; serum; immuno-PCR
Mafi et al. (2019) [55]	Iran	Four-arm RCT; older men with sarcopenia	68/62	8 weeks	RT with or without epicatechin; 3 sessions/week	Placebo or epicatechin-only matched strata	MSTN and FST; plasma in Methods; figure captions say serum
Ghobadi et al. (2024) [56]	Iran	Two-arm RCT; recreationally active men	30/30	8 weeks	Autoregulated versus linear periodized resistance exercise; 3 sessions/week	Active RT comparator	Circulating MSTN/FST
Willoughby (2004) [57]	USA	Two-arm RCT; untrained young men	22/22	12 weeks	Heavy resistance training; 3 sessions/week	Nonexercise control	MSTN; serum and muscle protein/mRNA; noncommercial immunoassay
Koczulla et al. (2020) [58]	Germany	Two-arm RCT; adults with interstitial lung disease	31/26	12 weeks	Whole-body vibration at therapeutic frequency	Low-frequency sham vibration with the same exercises	MSTN; circulating
Bagheri et al. (2021) [59]	Iran	Three-arm RCT; postmenopausal women	30/30	8 weeks	RT + placebo or RT + BCAA; 3 sessions/week	Nonexercise control; food provision not clearly matched	MSTN and FST; serum; ELISA
Saremi et al. (2010) [60]	Iran	Three-arm RCT; young men	27/24	8 weeks	RT + placebo or RT + creatine; 3 sessions/week	Nonexercise control	MSTN; serum; ELISA
Lenk et al. (2012) [61]	Germany	Two-arm RCT; adults with advanced heart failure	24/24	12 weeks	Supervised exercise training	Sedentary control	Muscle *MSTN* mRNA/protein; circulating MSTN mentioned
Shabkhiz et al. (2021) [62]	Iran	Stratified RCT; older men with or without type 2 diabetes	44/44	12 weeks	Resistance training; 3 sessions/week	Nonexercise within each health stratum	MSTN; circulating immunoassay
Schiffer et al. (2011) [63]	Germany	Three-arm RCT; physically active sports students	21/21	12 weeks	Strength or moderate endurance training	Nonexercise control	*MSTN* mRNA; vastus lateralis
Heisterberg et al. (2018) [64]	Denmark	Three-arm RCT; older men	71/58	16 weeks	Heavy RT plus losartan or placebo	Losartan without RT	MSTN expression; skeletal muscle; RT-PCR
Micielska et al. (2019) [65]	Poland	Nonrandomized controlled trial; inactive women	33/33	5 weeks	High-intensity circuit training; 15 sessions	No chronic training; two assessment bouts	MSTN; circulating
Nilsen et al. (2016) [66]	Norway	RCT biopsy substudy; men receiving ADT for prostate cancer	37/NR	16 weeks	Strength training	Usual care without strength training	MSTN protein; muscle cellular measurements
Hulmi et al. (2009) [67]	Finland	Three-arm RCT; young men	38/31	21 weeks	RT twice weekly with protein or placebo	No resistance training	*MSTN* mRNA; vastus lateralis
Lewis et al. (2007) [68]	USA	Factorial RCT biopsy substudy; men with COPD	40/40	10 weeks	Resistance training with or without testosterone	Matched no-training hormone stratum	*MSTN* mRNA; skeletal muscle
Boeselt et al. (2017) [69]	Germany	Nonrandomized controlled study; adults with COPD	49/37	3 months comparable follow-up	Individualized high-intensity aerobic/strength training twice weekly	No intervention	MSTN; serum; ELISA
Santos et al. (2015) [70]	Brazil	Randomized strength-versus-power comparison with separately recruited controls; young men	29/29	8 weeks	Strength versus power resistance-training regimens; 3 sessions/week	Power versus strength is randomized; separate nontraining control	*MSTN* and canonical *FST* gene expression; muscle
Lundberg et al. (2013) [71]	Sweden	Within-person leg comparison; men	10/10	5 weeks	Unilateral aerobic plus resistance exercise	Contralateral resistance-only training	*MSTN* gene expression; vastus lateralis
Laurentino et al. (2012) [72]	Brazil	Three-arm RCT; physically active men	29/29	8 weeks	Low-load RT with blood-flow restriction or high-load RT	Low-load RT without restriction	MSTN/FST expression; skeletal muscle
Walker et al. (2004) [73]	New Zealand	Two-arm randomized training comparison; healthy men	20/17	10 weeks	Whole-body resistance training	Elbow-flexor resistance training	MSTN; plasma Western blot
Irimia et al. (2017) [74]	France	Nonrandomized controlled bed-rest study; men	21/21	84 days	Flywheel resistance exercise during bed rest	Bed rest without exercise	*MSTN* gene expression; vastus lateralis
Mendez-Gutierrez et al. (2023) [75]	Spain	RCT substudy (ACTIBATE); sedentary young adults	110/110	24 weeks	Moderate or vigorous concurrent exercise; 3 sessions/week	Nonexercise control	Canonical FST; plasma; immunoassay
Bozyilan et al. (2026) [76]	Turkey	Two-arm RCT; elite male kickboxers	40/40	8 weeks	Added HIIT; usual kickboxing retained in both groups	Usual kickboxing training	Circulating MSTN
Strojek et al. (2021) [77]	Poland	Two-arm RCT; men after radical prostatectomy	37/34	12 weeks	Supervised pelvic-floor muscle training twice weekly	No intervention	MSTN; circulating
Farzanegi et al. (2021) [78]	Iran	Four-arm RCT; sedentary male students	40/40	8 weeks	Upper-, lower-, or combined-body resistance training; 3 sessions/week	Nonexercise control	MSTN and FST; serum; ELISA
Barati et al. (2025) [79]	Iran	Two-arm RCT; sedentary young men	20/20	9 weeks	Linear versus weekly undulating periodized RT	Active RT comparator	FST; circulating
Shabani et al. (2018) [80]	Iran	Four-arm RCT; untrained women	35/35	8 weeks	Resistance, aerobic or combined training; 3 sessions/week, 65 min/session	Nonexercise control	MSTN; serum; ELISA
Karimifard et al. (2023) [81]	Iran	Three-arm RCT; recreationally trained men	30/NR	12 weeks	Rest-pause or traditional resistance training; 3 sessions/week	Nonexercise control	MSTN and FST; circulating
Bijeh et al. (2022) [82]	Iran	Four-arm RCT; older men	60/NR	12 weeks	RT with/without soy milk; 3 sessions/week	Matched soy milk or usual activity	Serum MSTN/FST and source-reported MSTN:FST ratio
Riahy (2024) [83]	Iran	Three-arm RCT; men with type 2 diabetes	45/37	12 weeks	HIIT or MICT; 3 sessions/week	Nonexercise control	MSTN; serum; ZellBio ELISA; 48 h postexercise
Niazi et al. (2025) [84]	Iran	Four-arm RCT; men with obesity	44/44	12 weeks	Circuit RT at 40/60/80% 1RM; 3 sessions/week	Nonexercise control	Serum MSTN and FST; R&D ELISA
Nasiri et al. (2022) [85]	Iran	Two-arm RCT; women with overweight or obesity	26/22	8 weeks	Progressive Pilates; 3 sessions/week	Nonexercise control	Serum MSTN and FST; Figure 2
Watson et al. (2022) [86]	UK	Randomized exercise substudy; adults with CKD	NR/41	12 weeks	Aerobic versus combined aerobic/RT	Active aerobic training	Circulating and muscle MSTN
Sönmez et al. (2026) [87]	Turkey	Randomized active-training comparison; adults with heart failure	32/23	6 weeks	HIIT versus moderate continuous training	Active training	Serum MSTN
Souza et al. (2014) [88]	Brazil	Four-arm RCT; untrained men	43/37	8 weeks	Strength, interval or concurrent training	Nonexercise control	Muscle *MSTN* mRNA; FSTL3 is not FST
Izaddoust et al. (2017) [89]	Iran	Controlled resistance-training report; adult women	16/16	8 weeks	Resistance training	Nonexercise control	Serum MSTN
Rasi et al. (2023) [90]	Iran	Two-arm RCT; middle-aged men with metabolic syndrome	20/20	12 weeks	Aerobic; 3 sessions/week, 45 min	Nonexercise control	MSTN; serum; 48 h
Hooshmandi et al. (2022) [91]	Iran	Two-arm RCT; older women with sarcopenia	30/30	8 weeks	High-intensity interval resistance training	Nonexercise control	MSTN/IGF-I and MSTN/CSA ratios
Azamian Jazi et al. (2024) [92]	Iran	Two-arm RCT; older women with overweight	28/28	12 weeks	Elastic-band resistance training; 3 sessions/week	Nonexercise control	MSTN; serum; 48 h
Jaafari et al. (2024) [93]	Iran	Three-arm randomized trial; young women	45/45	8 versus 12 weeks unresolved	Combined training with or without BFR	Nonexercise control	FST; serum; MSTN denominator unresolved
Barzegari Marvast et al. (2024) [94]	Iran	Two-arm RCT; sedentary older men	30/30	12 weeks	Progressive RT; 2–4 sessions/week	Nonexercise control	Serum MSTN and FST; 24/48 h conflict
Ramezani et al. (2022) [95]	Iran	Four-arm RCT; men with overweight or obesity	47/47	12 weeks	Low/moderate/high interval intensity; 3 sessions/week	Nonexercise control	MSTN; serum; 48 h

Note. Baseline/analyzed *n* indicates the total enrolled or allocated and the total analyzed or completing the reported trial or substudy, rather than intervention/control group sizes. NR, not reported or not clearly ascertainable; missing totals were not imputed. Outcome-specific sample sizes may differ from trial totals. Country indicates the study setting. Abbreviations: ADT, androgen-deprivation therapy; AET, aerobic exercise training; AST, astaxanthin; BFR, blood-flow restriction; CKD, chronic kidney disease; EAA, essential amino acids; ELISA, enzyme-linked immunosorbent assay; EMS, electromyostimulation; FST, follistatin; HIFT, high-intensity functional training; HIIT, high-intensity interval training; IMT, inspiratory muscle training; MSTN, myostatin; PCOS, polycystic ovary syndrome; qPCR, quantitative polymerase chain reaction; RCT, randomized controlled trial; RT, resistance training; WB-EMS, whole-body electromyostimulation.

**Table 2:** Primary meta-analytic estimates and heterogeneity statistics for circulating myostatin and follistatin.

Outcome	Model	Effects	Studies	Hedges’ *g*	95% CI	*p*	*I*^2^ (%)	95% Prediction Interval
MSTN	Multilevel REML + CR2	57	33	1.25	0.68 to 1.82	<0.001	—	—
MSTN	Conventional REML (working independence)	57	33	1.41	0.96 to 1.86	<0.001	92.56	−1.74 to 4.57
FST	Multilevel REML + CR2	42	23	1.58	0.92 to 2.23	<0.001	—	—
FST	Conventional REML (working independence)	42	23	1.94	1.34 to 2.55	<0.001	92.89	−1.46 to 5.35

Note. Multilevel REML with study-clustered CR2 inference was primary; conventional REML assumed working independence and supplied reference *I*^2^ and prediction intervals. Multilevel variance components were τ^2^ between = 2.4053 and τ^2^ within = 0.0262 for MSTN, and τ^2^ between = 2.1005 and τ^2^ within = 0.0440 for FST. Dashes denote statistics not estimated in that model. CI, confidence interval; CR2, cluster-robust small-sample covariance correction; REML, restricted maximum likelihood.

## Data Availability

The original data presented in the study are openly available in Zenodo at https://doi.org/10.5281/zenodo.23455332. The analysis code is available in the same repository.

## Supplementary Materials

The following supporting information can be downloaded at https://www.mdpi.com/article/10.3390/ijms27219657/s1.

## Abbreviations

The following abbreviations are used in this manuscript:

ActRIIB	Activin receptor type IIB
ADT	Androgen-deprivation therapy
AET	Aerobic exercise training
ALK4/5	Activin receptor-like kinases 4 and 5
AST	Astaxanthin
BFR	Blood-flow restriction
CI	Confidence interval
CKD	Chronic kidney disease
CR2	Bias-reduced cluster-robust variance correction
EAA	Essential amino acids
ELISA	Enzyme-linked immunosorbent assay
EMS	Electromyostimulation
FST	Follistatin
GDF-8	Growth differentiation factor 8
GRADE	Grading of Recommendations Assessment, Development and Evaluation
HIFT	High-intensity functional training
HIIT	High-intensity interval training
HTZ	Approximate Hotelling’s T^2^ test with Zhang correction
IMT	Inspiratory muscle training
MSTN	Myostatin
NR	Not reported
PCOS	Polycystic ovary syndrome
PICOS	Population, Intervention, Comparator, Outcomes, and Study design
PRESS	Peer Review of Electronic Search Strategies
PRISMA	Preferred Reporting Items for Systematic Reviews and Meta-Analyses
PRISMA-S	PRISMA extension for reporting literature searches in systematic reviews
PROSPERO	International Prospective Register of Systematic Reviews
p-SMAD2/3	Phosphorylated SMAD family members 2 and 3
qPCR	Quantitative polymerase chain reaction
RCT	Randomized controlled trial
REML	Restricted maximum likelihood
RoB 2	Cochrane Risk of Bias 2 tool
ROBINS-I	Risk Of Bias In Non-randomized Studies of Interventions
RT	Resistance training
SD	Standard deviation
SE	Standard error
SMAD2/3	SMAD family members 2 and 3
SMAD4	SMAD family member 4
WB-EMS	Whole-body electromyostimulation
