# Supplementary material for: Exercise Modalities, Training Dose, and Circulating Myostatin and Follistatin in Adults: A Systematic Review and Multilevel Meta-Analysis
Source: Int J Mol Sci. 2026 Oct 28;27(21):9657. doi: 10.3390/ijms27219657 (PMC13763500; doi:10.3390/ijms27219657)
Supplement: Supplementary file 1 [file ijms-27-09657-s001.zip › PRISMA_Checklist.pdf]

PRISMA 2020 Checklist

Section and Topic	Item #	Checklist item	Location where item is reported
**TITLE**			
Title	1	Identify the report as a systematic review.	Revised manuscript, title, p. 1, lines 2–4.
**ABSTRACT**			
Abstract	2	See the PRISMA 2020 for Abstracts checklist.	Revised manuscript, Abstract, p. 1, lines 13–28.
**INTRODUCTION**			
Rationale	3	Describe the rationale for the review in the context of existing knowledge.	Revised manuscript, Introduction, pp. 1–3.
Objectives	4	Provide an explicit statement of the objective(s) or question(s) the review addresses.	Revised manuscript, final paragraph of Introduction, pp. 2–3.
**METHODS**			
Eligibility criteria	5	Specify the inclusion and exclusion criteria for the review and how studies were grouped for the syntheses.	Revised manuscript, Section 2.2, p. 3, lines 104–136; Section 2.8, pp. 6–7; Supplementary Methods, pp. 1–2.
Information sources	6	Specify all databases, registers, websites, organisations, reference lists and other sources searched or consulted to identify studies. Specify the date when each source was last searched or consulted.	Revised manuscript, Section 2.3, pp. 3–4; Supplementary File S1, pp. 53–54.
Search strategy	7	Present the full search strategies for all databases, registers and websites, including any filters and limits used.	Supplementary File S1, full electronic search strategies, pp. 53–54; revised manuscript, Section 2.3, pp. 3–4.
Selection process	8	Specify the methods used to decide whether a study met the inclusion criteria of the review, including how many reviewers screened each record and each report retrieved, whether they worked independently, and if applicable, details of automation tools used in the process.	Revised manuscript, Section 2.4, p. 4, lines 162–177; Section 2.5, p. 5, lines 195–202; Supplementary Methods, pp. 1–2.
Data collection process	9	Specify the methods used to collect data from reports, including how many reviewers collected data from each report, whether they worked independently, any processes for obtaining or confirming data from study investigators, and if applicable, details of automation tools used in the process.	Revised manuscript, Section 2.5, pp. 4–5, lines 178–202; Supplementary Methods, pp. 1–2.
Data items	10a	List and define all outcomes for which data were sought. Specify whether all results that were compatible with each outcome domain in each study were sought (e.g. for all measures, time points, analyses), and if not, the methods used to decide which results to collect.	Revised manuscript, Sections 2.2 and 2.5, pp. 3–5; Supplementary Tables S3A (pp. 18–27) and S9 (pp. 44–48); Supplementary File S2, source and comparison tables.
	10b	List and define all other variables for which data were sought (e.g. participant and intervention characteristics, funding sources). Describe any assumptions made about any missing or unclear information.	Revised manuscript, Section 2.5, pp. 4–5; Table 1, pp. 9–13; Supplementary Tables S2, S3A and S3B, pp. 12–32.
Study risk of bias assessment	11	Specify the methods used to assess risk of bias in the included studies, including details of the tool(s) used, how many reviewers assessed each study and whether they worked independently, and if applicable, details of automation tools used in the process.	Revised manuscript, Section 2.6, p. 5, lines 203–220; Supplementary Methods, pp. 1–2; Tables S8A–S8B, pp. 41–43.
Effect measures	12	Specify for each outcome the effect measure(s) (e.g. risk ratio, mean difference) used in the synthesis or presentation of results.	Revised manuscript, Section 2.7, p. 5, lines 221–239; Supplementary Methods, pp. 1–2.
Synthesis	13a	Describe the processes used to decide which studies were eligible for each synthesis (e.g. tabulating the study	Revised manuscript, Sections 2.2 and

PRISMA 2020 Checklist

Section and Topic	Item #	Checklist item	Location where item is reported
methods		intervention characteristics and comparing against the planned groups for each synthesis (item #5)).	2.8, pp. 3 and 6–7; Supplementary Tables S2 and S10, pp. 12–18 and 48–52.
	13b	Describe any methods required to prepare the data for presentation or synthesis, such as handling of missing summary statistics, or data conversions.	Revised manuscript, Sections 2.5 and 2.7, pp. 4–5; Supplementary Methods, pp. 1–2; Tables S3A–S3B, pp. 18–32.
	13c	Describe any methods used to tabulate or visually display results of individual studies and syntheses.	Revised manuscript, Section 2.8, pp. 6–7; Figure 2 legend, p. 14, lines 369–378; Figure 3 legend, p. 16, lines 430–436.
	13d	Describe any methods used to synthesize results and provide a rationale for the choice(s). If meta-analysis was performed, describe the model(s), method(s) to identify the presence and extent of statistical heterogeneity, and software package(s) used.	Revised manuscript, Section 2.8, pp. 6–7, lines 240–315; Table 2, p. 15; Supplementary File S2, reproducible code and model results.
	13e	Describe any methods used to explore possible causes of heterogeneity among study results (e.g. subgroup analysis, meta-regression).	Revised manuscript, Section 2.8, pp. 6–7, lines 262–301; Supplementary Tables S5A–S6B, pp. 37–40.
	13f	Describe any sensitivity analyses conducted to assess robustness of the synthesized results.	Revised manuscript, Section 2.8, p. 6, lines 254–261 and 285–290; Supplementary Table S4, pp. 32–37.
Reporting bias assessment	14	Describe any methods used to assess risk of bias due to missing results in a synthesis (arising from reporting biases).	Revised manuscript, Section 2.8, p. 7, lines 302–306; formal asymmetry tests were not performed, with rationale stated.
Certainty assessment	15	Describe any methods used to assess certainty (or confidence) in the body of evidence for an outcome.	Revised manuscript, Section 2.6, p. 5, lines 213–220; Supplementary Table S7, pp. 40–41.
**RESULTS**			
Study selection	16a	Describe the results of the search and selection process, from the number of records identified in the search to the number of studies included in the review, ideally using a flow diagram.	Revised manuscript, Section 3.1, p. 7, lines 317–334; Figure 1 and legend, p. 8, lines 336–341.
	16b	Cite studies that might appear to meet the inclusion criteria, but which were excluded, and explain why they were excluded.	Supplementary Table S1, pp. 4–12, identifies 124 excluded retrieved reports with one primary reason each; revised manuscript, Section 3.1, p. 7.
Study characteristics	17	Cite each included study and present its characteristics.	Revised manuscript, Section 3.2, pp. 8–9; Table 1, pp. 9–13; Supplementary Table S2, pp. 12–18.
Risk of bias in studies	18	Present assessments of risk of bias for each included study.	Revised manuscript, Section 3.3.3, p. 15, lines 392–399; Supplementary Figures S1–S2, pp. 3–4; Tables S8A–S8B, pp. 41–43.
Results of individual studies	19	For all outcomes, present, for each study: (a) summary statistics for each group (where appropriate) and (b) an effect estimate and its precision (e.g. confidence/credible interval), ideally using structured tables or plots.	Revised manuscript, Figure 2, p. 14; Supplementary Tables S3A (pp. 18–27)

Section and Topic	Item #	Checklist item	Location where item is reported
			and S9 (pp. 44–48); Supplementary File S2, group summaries and effect-size inputs.
Results of syntheses	20a	For each synthesis, briefly summarise the characteristics and risk of bias among contributing studies.	Revised manuscript, Section 3.2, pp. 8–9; Section 3.3.3, p. 15; Table 1, pp. 9–13; Supplementary Tables S2 and S8A–S8B.
	20b	Present results of all statistical syntheses conducted. If meta-analysis was done, present for each the summary estimate and its precision (e.g. confidence/credible interval) and measures of statistical heterogeneity. If comparing groups, describe the direction of the effect.	Revised manuscript, Sections 3.3.1–3.3.6, pp. 13–16; Figures 2–3; Table 2; Supplementary Tables S3A–S6B and S9.
	20c	Present results of all investigations of possible causes of heterogeneity among study results.	Revised manuscript, Section 3.3.5, pp. 15–16, lines 415–428; Supplementary Tables S5A–S6B, pp. 37–40.
	20d	Present results of all sensitivity analyses conducted to assess the robustness of the synthesized results.	Revised manuscript, Section 3.3.4, p. 15, lines 403–414; Supplementary Table S4, pp. 32–37; Supplementary Table S5C, p. 38.
Reporting biases	21	Present assessments of risk of bias due to missing results (arising from reporting biases) for each synthesis assessed.	Revised manuscript, Section 3.3.3, p. 15, lines 400–402; Strengths and Limitations, p. 19, lines 563–575; Supplementary Table S7. No formal asymmetry tests were performed.
Certainty of evidence	22	Present assessments of certainty (or confidence) in the body of evidence for each outcome assessed.	Revised manuscript, Section 3.3.3, p. 15, lines 398–399; Supplementary Table S7, pp. 40–41. Both outcomes have Low certainty.
**DISCUSSION**			
Discussion	23a	Provide a general interpretation of the results in the context of other evidence.	Revised manuscript, Discussion, Sections 4.1–4.3, pp. 16–18.
	23b	Discuss any limitations of the evidence included in the review.	Revised manuscript, Strengths and Limitations, p. 19, lines 563–575.
	23c	Discuss any limitations of the review processes used.	Revised manuscript, Strengths and Limitations, p. 19, lines 563–575; Supplementary Methods, pp. 1–2; evidence update, p. 48.
	23d	Discuss implications of the results for practice, policy, and future research.	Revised manuscript, Section 4.4, pp. 18–19; Conclusions, p. 19, lines 576–580.
**OTHER INFORMATION**			
Registration and protocol	24a	Provide registration information for the review, including register name and registration number, or state that the review was not registered.	Revised manuscript, Section 2.1, p. 3, lines 95–103. PROSPERO CRD420261492548.

PRISMA 2020 Checklist

Section and Topic	Item #	Checklist item	Location where item is reported
	24b	Indicate where the review protocol can be accessed, or state that a protocol was not prepared.	Revised manuscript, Section 2.1, p. 3, lines 95–103; PROSPERO CRD420261492548. The registration record is the public protocol record; no separate protocol document is supplied.
	24c	Describe and explain any amendments to information provided at registration or in the protocol.	Revised manuscript, Section 2.1, p. 3, lines 99–101; Sections 2.5 and 2.8; Supplementary Methods, pp. 1–2; evidence update, p. 48; Tables S9–S10, pp. 44–52.
Support	25	Describe sources of financial or non-financial support for the review, and the role of the funders or sponsors in the review.	Revised manuscript, Funding, p. 20, line 604. No external funding.
Competing interests	26	Declare any competing interests of review authors.	Revised manuscript, Conflicts of Interest, p. 20, line 615.
Availability of data, code and other materials	27	Report which of the following are publicly available and where they can be found: template data collection forms; data extracted from included studies; data used for all analyses; analytic code; any other materials used in the review.	Revised manuscript, Section 2.8, p. 7, lines 307–315; Data Availability Statement, p. 20, lines 608–609; Supplementary File S2. Aggregate extraction data, analysis inputs, R scripts and results are publicly available at https://doi.org/10.5281/zenodo.23455332.

From: Page MJ, McKenzie JE, Bossuyt PM, Boutron I, Hoffmann TC, Mulrow CD, et al. The PRISMA 2020 statement: an updated guideline for reporting systematic reviews. BMJ 2021;372:n71. doi: 10.1136/bmj.n71. This work is licensed under CC BY 4.0. To view a copy of this license, visit https://creativecommons.org/licenses/by/4.0/
