# Supplementary material for: Exercise Modalities, Training Dose, and Circulating Myostatin and Follistatin in Adults: A Systematic Review and Multilevel Meta-Analysis
Source: Int J Mol Sci. 2026 Oct 28;27(21):9657. doi: 10.3390/ijms27219657 (PMC13763500; doi:10.3390/ijms27219657)
Supplement: Supplementary file 1 [file ijms-27-09657-s001.zip › Supplementary_Material.pdf]

Supplementary Material

This supplementary file accompanies the systematic review and multilevel meta-analysis entitled "Exercise Modalities, Training Dose, and Circulating Myostatin and Follistatin in Adults: A Systematic Review and Multilevel Meta-Analysis". It reports the prespecified eligibility framework, complete search syntax, study-level source data, effect-size inputs, robustness analyses, certainty assessments, and risk-of-bias judgements supporting the main manuscript. The review was registered in PROSPERO (CRD420261492548).

Supplementary Methods

Eligibility framework and outcome hierarchy

Eligibility was operationalised using PICOS. Adults aged 18 years or older were eligible irrespective of health status. Interventions had to comprise structured exercise training lasting at least 4 weeks, with a concurrent eligible comparator. Randomised and non-randomised longitudinal intervention studies were considered. Eligible outcomes were myostatin (MSTN/GDF-8) or classical follistatin (FST) measured in blood, serum, plasma, or skeletal muscle. Acute exercise studies, paediatric or non-human studies, uncontrolled pre–post studies, inseparable co-interventions, non-structured physical activity exposures, and ineligible publication types were excluded. Follistatin-related protein 3 (FSTL3) was outside the predefined canonical FST outcome, which did not include FST-related proteins.

Search strategy and record management

Retrieval was sought for 220 reports. Renewed retrieval through interlibrary loan, institutional access, author contact and publisher purchase supplied 99 research-full-text records (98 unique texts) and 22 original conference reports from the original 138-record queue; one related conference version did not replace its original journal report. Together with the previously assessed 82 reports, 203 reports underwent eligibility assessment; 17 originals remained unavailable. Downloaded version identities and the author Word-to-PDF conversion were retained. One primary reason was recorded per excluded retrieved report (Supplementary Table S1 and File S2).

Study selection and report retrieval

After deduplication, 1,620 records formed the unique screening baseline; 1,229 were excluded by title/abstract and 171 registry records were retained separately for linkage. Of 220 reports sought, 203 were assessed and 17 remained unavailable. Assessment excluded 124 retrieved reports; 79 reports represented 78 study families. Historical reviewer decisions and adjudication were preserved, and the latest author eligibility corrections are recorded separately (Supplementary Table S1).

Study linkage, extraction, and effect-size hierarchy

Reports were linked to study families before extraction. ChatGPT (OpenAI; model GPT-5.6 Sol; application version 26.1013.40934) assisted preliminary screening and the location of bibliographic records and relevant source passages. CQ and CY independently extracted and cross-checked study characteristics, exercise prescriptions, comparators, biological specimens, assay methods and outcome data against the source reports, including the recovered evidence. They also reviewed domain-level risk judgments. Unresolved source limitations remain explicit. Final eligibility decisions and extraction data remained the responsibility of CQ and CY; AI-assisted screening and source-location outputs were not accepted as final decisions or data. Circulating and muscle-expression outcomes were kept distinct. Primary circulating syntheses used exercise-versus-nonexercise contrasts with matched nonexercise co-interventions where applicable; direct active-training comparisons were not pooled with them. When multiple eligible intervention arms shared a comparator, the dependency structure was retained by study and shared-control identifiers. Post-intervention or change-score data were used according to the prespecified hierarchy, and standard errors or 95% confidence intervals were converted to standard deviations only when required and traceable (Supplementary Tables S2 and S3). During revision, omitted circulating inputs were restored and numerical values unavailable in tables were digitized from Arazi Figure 3 and Mahboobi Semnan Figures 3–4. Source images, pixel calibrations and extracted values were retained. These are approximate figure-derived summaries, not individual participant data. Arazi's error convention followed its statistical-methods statement (SD); an SEM alternative and exclusion of both newly digitized studies were evaluated in Table S4.

Effect-size derivation and primary meta-analysis

Standardization formulae. Let n_I and n_C be the reported arm sizes used in the effect-size calculation and $\nu = n\_I + n\_C - 2$. The pooled variance was $s\_p^2 = [(n\_I - 1)s\_I^2 + (n\_C - 1)s\_C^2]/\nu$. Cohen's d was $(\text{mean\_I} - \text{mean\_C})/s\_p$ and Hedges' $g = Jd$, using $J = 1 - 3/(4\nu - 1)$. The sign was reversed for MSTN so that positive values denote lower MSTN; positive FST values denote higher FST. This sign convention is not a clinical-benefit criterion.

For postintervention effects, the arm means and SDs were final-value summaries. For change-score effects, mean_I and mean_C were the reported arm-level post-minus-pre changes, and s_I and s_C were the reported change-score SDs. Thus, its denominator was the pooled change-score SD, not the SD of baseline values, final values, or the difference between arm means. It is not a standardized paired within-arm change or a reconstructed ANCOVA coefficient. The Ghayomzadeh source labels Table 3 change summaries as adjusted; its exact underlying summary construction should not be inferred solely from the mean ± SD label. Baseline assessment distinguished target-marker arm summaries from general demographic comparability and from the original investigators' model adjustment. A nonsignificant baseline test was not treated as proof of balance. Available baseline tables and previously extracted graphical summaries were reviewed; values not numerically verified were not assumed balanced. Baseline MSTN differences were evident in Rezaei and Seo, supporting caution with postintervention-only SMDs. For Dantas, POST3, immediately before the exercise phase, was distinguished from the presurgical PRE assessment. The arm-summary SMD calculation did not inherit covariate

adjustment from a source ANCOVA or mixed model; no participant-level baseline adjustment was reconstructed. Baseline differences alone were not used as a post hoc exclusion rule.

Hedges' g was calculated from continuous outcomes with small-sample correction. Effects were oriented so that positive values represented lower circulating MSTN or higher circulating FST, without implying biological or clinical benefit. MSTN and FST were analysed separately. The primary model was a multilevel random-effects meta-analysis with restricted maximum likelihood estimation and cluster-robust CR2 inference at the study level. Conventional random-effects models under working independence were retained as secondary descriptors. Prediction intervals and variance components were reported where estimable (Supplementary Tables S3 and S4).

Modality, dose, and robustness analyses

Exercise-modality grouping was data-informed, post hoc and exploratory, not established as prospectively prespecified. Rules formalized during revision prioritized explicit mixed strength/endurance HIFT/CrossFit workouts, then predominantly resistance-loading protocols, then endurance or concurrent aerobic/resistance protocols. Interval resistance training remained resistance-based unless an explicit mixed HIFT/CrossFit prescription was reported. The term functional alone did not determine HIFT membership. Other targeted modalities and active training arms were recorded separately rather than forced into a primary moderator group. Table S10 reports assignments for all 79 included reports representing 78 study families and identifies their primary-analysis contributions. The updated primary evidence comprises 39 study families; a study may contribute more than one arm-specific modality. The binary comparison is conventional resistance-based training versus other modalities, not the presence versus absence of any resistance exercise. Modality subgroup pools used within-study aggregation at $\rho = 0.5$ and REML with modified Knapp–Hartung inference, bounding the variance multiplier below by 1 and using $k - 1$ degrees of freedom. This correction was introduced during revision. Omnibus moderator tests, meta-regression, variance-explained diagnostics, resistance-only restriction, HIFT/CrossFit exclusion and leave-one-modality-class-out analyses remained exploratory and were not interpreted as rankings. Dose analyses were limited to variables with sufficient coverage and independent-study variation. Sensitivity analyses varied the assumed within-study correlation (ρ=0, 0.5, and 0.8), excluded extreme effects where prespecified, aggregated dependent effects, and iteratively omitted individual studies (Supplementary Tables S4, S5C and S6B). Category restrictions used the same modified Knapp–Hartung rule. Duration and total sessions were modeled separately per 4 weeks and 12 sessions; neither coefficient represents a mutually adjusted causal effect. The available-case sets need not coincide when session counts are missing, and complete collinearity is not assumed. Exploratory P values are unadjusted for multiplicity and should not be interpreted as independent confirmatory tests. Frequency was not modeled because twice-weekly training was represented by only three MSTN studies and one FST study. Newly recovered dose information was incorporated during revision. The aggregate extraction data, analysis inputs, R scripts and numerical results are openly available in Zenodo at https://doi.org/10.5281/zenodo.23455332.

Risk of bias and certainty of evidence

Tool allocation followed the allocation underlying the extracted comparison rather than its design label. Reported random allocation was assessed with RoB 2; insufficient sequence or concealment reporting was evaluated within its domains. Nonrandom allocation, including a control group augmented after randomization, was assessed with ROBINS-I. For RoB 2, the effect of interest was assignment to intervention; adherence-related exclusions were considered in D2 and missing outcomes in D3. The inability to blind exercise participants or intervention providers was not, by itself, used to assign high risk; the judgement was escalated only when lack of blinding plausibly affected deviations from intended intervention or outcome measurement. Revised tool assignments and domain-level justifications are provided in Supplementary Tables S2, S8A and S8B. Certainty was rated separately for circulating MSTN and FST using GRADE (Supplementary Figures S1 and S2; Supplementary Table S7; Supplementary Tables S8A and S8B).

Supplementary Figures

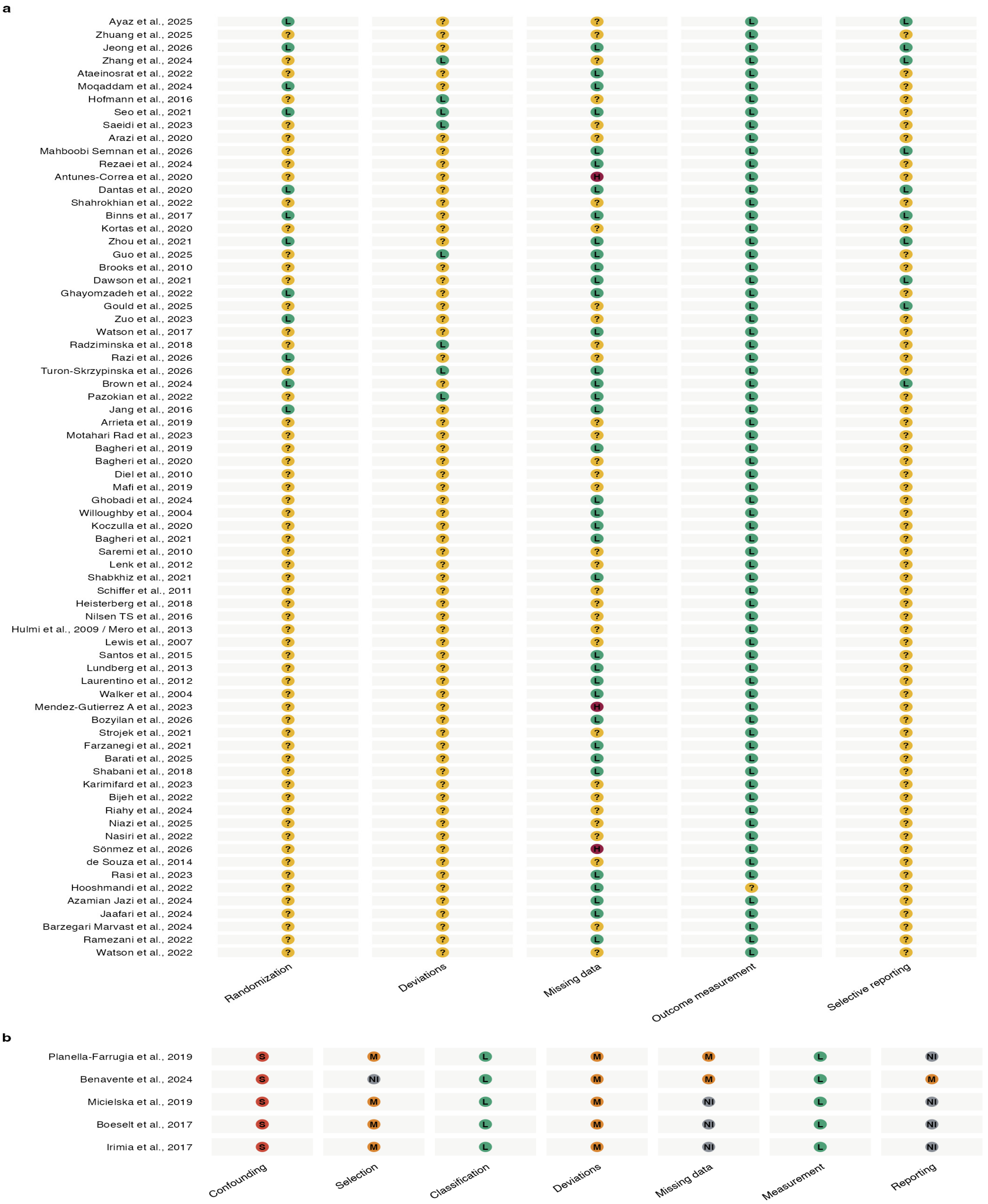

Supplementary Figure S1. Domain-level risk-of-bias traffic-light plots for 78 study families. (a) RoB 2 judgments for 73 randomized study families. (b) ROBINS-I judgments for five nonrandomized study families. CQ and CY reviewed the assessments. The family-level display uses the more cautious eligible-outcome judgment when outcomes differ; Hooshmandi's laboratory MSTN/IGF-I ratio had Low measurement risk, whereas the ultrasound-dependent MSTN/CSA ratio had Some concerns. Outcome-specific reasons are retained in Table S8 and File S2. ROBINS-I uses No information, not Some concerns, where information is insufficient.

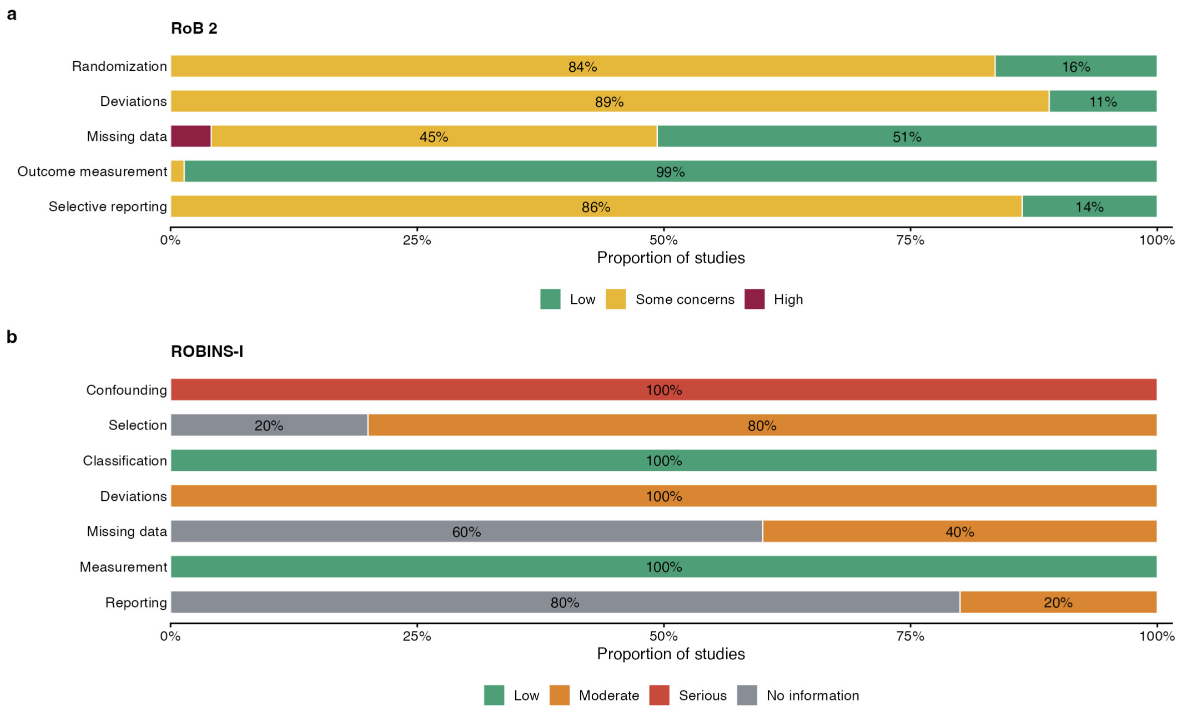

Supplementary Figure S2. Distribution of domain-level risk-of-bias judgments. (a) RoB 2 assessments for 73 randomized study families. (b) ROBINS-I assessments for five nonrandomized study families. Each panel uses its own tool-specific categories. The two panels describe distinct assessment sets and should not be combined into a common percentage.

Supplementary Tables

Supplementary Table S1. Reports excluded after full-text assessment and the single primary reason for exclusion

Author, year	Report title	Primary reason for exclusion
Pourabbas, M. (2021)	Strategic ingestion of high-protein dairy milk during a resistance training program increases lean mass, strength, and power in trained young males	Inseparable co-intervention
McIntosh, M.C. (2023)	Different Resistance Exercise Loading Paradigms Similarly Affect Skeletal Muscle Gene Expression Patterns of Myostatin-Related Targets and mTORC1 Signaling Markers	Acute intervention or duration <4 weeks
Ziyaiyan A (2023)	High-intensity circuit training change serum myostatin but not myogenin in adolescents' soccer players: a quasi-experimental study.	Paediatric population
Paoli, A (2015)	Protein supplementation increases postexercise plasma myostatin concentration after 8 weeks of resistance training in young physically active subjects	Inseparable co-intervention
Kvorning, T. (2007)	Suppression of testosterone does not blunt mRNA expression of myoD, myogenin, IGF, myostatin or androgen receptor post strength training in humans	Inseparable co-intervention
Schwarz, NA (2016)	Effect of resistance exercise intensity on the expression of PGC-1α isoforms and the anabolic and catabolic signaling mediators, IGF-1 and myostatin, in human skeletal muscle	Acute intervention or duration <4 weeks
Kerr, A. (2023)	Improved Strength Recovery and Reduced Fatigue with Suppressed Plasma Myostatin Following Supplementation of a Vicia faba Hydrolysate, in a Healthy Male Population	Acute intervention or duration <4 weeks

Author, year	Report title	Primary reason for exclusion
Sawicka, A.K. (2022)	L-Carnitine Combined with Leucine Supplementation Does Not Improve the Effectiveness of Progressive Resistance Training in Healthy Aged Women	Inseparable co-intervention
Zhao, C. (2025)	The effects of vitamin D combined with thermotherapy and resistance exercise on inflammatory factors, thyroid hormones and MSTN in elderly patients with sarcopenia	Inseparable co-intervention
Haß, U. (2022)	Effects of an Omega-3 Supplemented, High-Protein Diet in Combination with Vibration and Resistance Exercise on Muscle Power and Inflammation in Old Adults: A Pilot Randomized Controlled Trial	Inseparable co-intervention
Martínez-Noguera FJ (2025)	Comparison of Hormonal, Inflammatory, Muscle Damage and Oxidative Stress Biomarkers Changes in Response to High-Intensity Interval, Circuit and Concurrent Exercise Bouts.	Acute intervention or duration <4 weeks
Amasene M (2021)	Effects of Resistance Training Intervention along with Leucine-Enriched Whey Protein Supplementation on Sarcopenia and Frailty in Post-Hospitalized Older Adults: Preliminary Findings of a Randomized Controlled Trial.	Inseparable co-intervention
Cotter, J.A. (2015)	Concurrent exercise on a gravity-independent device during simulated microgravity	Acute intervention or duration <4 weeks
Agergaard J (2015)	Does vitamin-D intake during resistance training improve the skeletal muscle hypertrophic and strength response in young and elderly men? - a randomized controlled trial.	Inseparable co-intervention
Ivey, FM (2017)	Strength Training for Skeletal Muscle Endurance after Stroke	No eligible MSTN or FST outcome
Hostrup, M. (2018)	Beta2-adrenoceptor agonist salbutamol increases protein turnover rates and alters signalling in skeletal muscle after resistance exercise in young men	Acute intervention or duration <4 weeks
Manini, T.M. (2011)	Myogenic and proteolytic mRNA expression following blood flow restricted exercise	Acute intervention or duration <4 weeks
Mosler S (2014)	Combinatory effects of siRNA-induced myostatin inhibition and exercise on skeletal muscle homeostasis and body composition.	Non-human study
Allen, D.L. (2011)	Expression and function of myostatin in obesity, diabetes, and exercise adaptation	Not an eligible longitudinal intervention
Ryan, A.S. (2011)	Skeletal muscle hypertrophy and muscle myostatin reduction after resistive training in stroke survivors	No eligible concurrent comparator
Konopka AR (2010)	Molecular adaptations to aerobic exercise training in skeletal muscle of older women.	No eligible concurrent comparator
Hittel, D.S. (2010)	Myostatin decreases with aerobic exercise and associates with insulin resistance	No eligible concurrent comparator
Williamson DL (2010)	Resistance exercise, skeletal muscle FOXO3A, and 85-year-old women.	No eligible concurrent comparator
Jensky, N.E. (2010)	Exercise does not influence myostatin and follistatin messenger rna expression in young women	Acute intervention or duration <4 weeks
Andonian, B.J. (2018)	Effect of high-intensity interval training on muscle remodeling in rheumatoid arthritis compared to prediabetes	No eligible concurrent comparator
Alexanderson H (2018)	Exercise in Myositis.	Not an eligible longitudinal intervention

Author, year	Report title	Primary reason for exclusion
Konopka AR (2018)	Relationship between intermuscular adipose tissue infiltration and myostatin before and after aerobic exercise training.	No eligible concurrent comparator
Perakakis, N. (2018)	Regulation of the activins-follistatins-inhibins axis by energy status: Impact on reproductive function	Not structured exercise training
Han, D.-S. (2016)	Association of serum myokines and aerobic exercise training in patients with spinal cord injury: An observational study	No eligible concurrent comparator
Willoughby DS (2022)	Resistance Exercise-Induced Increases in Muscle Myostatin mRNA and Protein Expression Are Subsequently Decreased in Circulation in the Presence of Increased Levels of the Extracellular Matrix Stabilizing Protein Decorin.	Acute intervention or duration <4 weeks
Santos HO (2022)	The Effects of Dietary Supplements, Nutraceutical Agents, and Physical Exercise on Myostatin Levels: Hope or Hype?	Not an eligible longitudinal intervention
Acevedo, O.G. (2022)	Seawater Hydration Modulates IL-6 and Apelin Production during Triathlon Events: A Crossover Randomized Study	Acute intervention or duration <4 weeks
Alizadeh Pahlavani H (2022)	Exercise Therapy for People With Sarcopenic Obesity: Myokines and Adipokines as Effective Actors.	Not an eligible longitudinal intervention
Petrie MA (2022)	Genomic and Epigenomic Evaluation of Electrically Induced Exercise in People With Spinal Cord Injury: Application to Precision Rehabilitation.	Acute intervention or duration <4 weeks
Almquist NW (2021)	Effects of including sprints during prolonged cycling on hormonal and muscular responses and recovery in elite cyclists.	Acute intervention or duration <4 weeks
McDonald, C.M. (2021)	(−)-Epicatechin induces mitochondrial biogenesis and markers of muscle regeneration in adults with Becker muscular dystrophy	Not structured exercise training
Jaworska, J. (2020)	Short-term resistance training supported by whole-body cryostimulation induced a decrease in myostatin concentration and an increase in isokinetic muscle strength	Inseparable co-intervention
Negaresh R (2019)	Skeletal Muscle Hypertrophy, Insulin-like Growth Factor 1, Myostatin and Follistatin in Healthy and Sarcopenic Elderly Men: The Effect of Whole-body Resistance Training.	No eligible concurrent comparator
Tichy, L. (2025)	Concurrent Physical Activity Protects Against C26 Adenocarcinoma Tumor-Mediated Cardiac and Skeletal Muscle Dysfunction and Wasting in Males	Non-human study
Peng, L.-N. (2024)	Protein-enriched soup and weekly exercise improve muscle health: A randomized trial in mid-to-old age with inadequate protein intake	Inseparable co-intervention
Wang S (2026)	Partial ischemia as molecular medicine: molecular mechanisms and clinical horizons of blood flow restriction training.	Not an eligible longitudinal intervention
Xiang B (2026)	Sarcopenia in breast cancer: prognostic values and emerging therapeutic strategies.	Not an eligible longitudinal intervention
Santos AR (2014)	Blood flow restricted resistance training attenuates myostatin gene expression in a patient with inclusion body myositis.	Ineligible publication type
Schwarz NA (2018)	(-)-Epicatechin Supplementation Inhibits Aerobic Adaptations to Cycling Exercise in Humans.	Inseparable co-intervention
Kishida, T. (2023)	Dietary fish protein improves skeletal muscle weight	Ineligible publication type

Author, year	Report title	Primary reason for exclusion
Ryan et al., 2013	Aerobic exercise + weight loss decreases skeletal muscle myostatin expression and improves insulin sensitivity in older adults	No eligible concurrent comparator. Single-group aerobic exercise plus weight loss; the duplicate publication does not provide an additional study.
Jerobin et al., 2021	Regulation of circulating CTRP-2/CTRP-9 and GDF-8/GDF-15 by intralipids and insulin in healthy control and polycystic ovary syndrome women following chronic exercise training	No eligible concurrent comparator
Micielska et al., 2021	Nordic Walking Rather Than High Intensity Interval Training Reduced Myostatin Concentration More Effectively in Elderly Subjects and the Range of This Drop Was Modified by Metabolites of Vitamin D.	Acute intervention or duration <4 weeks
Park et al., 2021	Complex Exercise Improves Anti-Inflammatory and Anabolic Effects in Osteoarthritis-Induced Sarcopenia in Elderly Women.	No eligible concurrent comparator
Bagheri, R. (2020)	Comparison of whole egg v. egg white ingestion during 12 weeks of resistance training on skeletal muscle regulatory markers in resistance-trained men	Nonisolatable exercise effect. Both arms received the same resistance training; whole-egg versus egg-white nutrition differs.
Willoughby, D.S. (2004)	Effects of an alleged myostatin-binding supplement and heavy resistance training on serum myostatin, muscle strength and mass, and body composition	Nonisolatable exercise effect. Both arms received identical resistance training; randomized contrast was Cystoseira supplement versus placebo (OCR *p*.1/3).
Dalbo, V.J. (2013)	Effects of pre-exercise feeding on serum hormone concentrations and biomarkers of myostatin and ubiquitin proteasome pathway activity	Acute/<4-week intervention. Single exercise bouts with biopsies 2 and 6 hours afterward; feeding crossover, not a chronic exercise contrast.
Thomis, M.A.I. (2004)	Exploration of myostatin polymorphisms and the angiotensin-converting enzyme insertion/deletion genotype in responses of human muscle to strength training	No eligible target outcome/design. Genotype association with training response; no eligible longitudinal MSTN/FST protein/expression outcome contrast.
Bagheri, R. (2022)	Effects of Icelandic yogurt consumption and resistance training in healthy untrained older males	Nonisolatable exercise effect. Identical resistance training in both groups; Icelandic yogurt versus isocaloric placebo differs.
Kazemi, F. (2016)	The correlation of resistance exercise-induced myostatin with insulin resistance and plasma cytokines in healthy young men	Acute/<4-week intervention. Single circuit resistance-exercise session; samples before and 24 h after.
D'Lugos, AC (2018)	Prior acetaminophen consumption impacts the early adaptive cellular response of human skeletal muscle to resistance exercise	Acute/<4-week intervention. Early acute resistance-exercise response with acetaminophen manipulation, not >=4-week controlled training.
Lundberg, T.R. (2014)	Exercise-induced AMPK activation does not interfere with muscle hypertrophy in response to resistance training in men	No eligible chronic target-outcome contrast. MSTN gene expression was assessed in the acute pre/3-hour experiment; the chronic resting biopsies assessed citrate synthase and glycogen, not a chronic MSTN/FST contrast.
Li, X. (2014)	The A55T and K153R polymorphisms of MSTN gene are associated with the strength training-induced muscle hypertrophy among Han Chinese men.	No eligible target outcome/design. MSTN polymorphism association with hypertrophy; genotype is not longitudinal MSTN/FST expression or concentration.
Besse-Patin, A. (2014)	Effect of endurance training on skeletal muscle myokine expression in obese men: Identification of apelin as a novel myokine	No eligible contemporaneous comparator. Eleven obese men all completed endurance training; myotube experiments do not provide a human training control.
Dieli-Conwright, C.M. (2012)	Hormone therapy and maximal eccentric exercise alters myostatin-related gene expression in postmenopausal women	Acute/<4-week intervention. Acute maximal eccentric bout in hormone-therapy users and nonusers.
Jespersen, J.G. (2011)	Myostatin expression during human muscle hypertrophy and subsequent atrophy: Increased myostatin with detraining	No eligible contemporaneous comparator. Sequential training and detraining in the same cohort; no concurrent target-outcome control.

Author, year	Report title	Primary reason for exclusion
Hansen, J. (2011)	Exercise induces a marked increase in plasma follistatin: Evidence that follistatin is a contraction-induced hepatokine	Acute/<4-week intervention. Acute contraction/hepatokine experiments, not chronic >=4-week exercise.
Vogiatzis, I. (2010)	Effect of pulmonary rehabilitation on muscle remodelling in cachectic patients with COPD	No eligible contemporaneous comparator. Cachectic and non-cachectic COPD cohorts both received the same 10-week cycling programme; disease status is not an exercise contrast.
Harber, M.P. (2009)	Protein synthesis and the expression of growth-related genes are altered by running in human vastus lateralis and soleus muscles	Acute/<4-week intervention. Acute running study and early postexercise biopsies, not chronic training.
Hulmi, J.J. (2008)	The effects of whey protein on myostatin and cell cycle-related gene expression responses to a single heavy resistance exercise bout in trained older men	Acute/<4-week intervention. Single resistance bout with peri-exercise whey feeding and short-term gene-expression responses.
Kopple, J.D. (2006)	Effect of Exercise on mRNA Levels for Growth Factors in Skeletal Muscle of Hemodialysis Patients	No eligible contemporaneous comparator. Ten hemodialysis patients underwent pre-post endurance training; eight paired biopsy specimens, no concurrent training control.
Huygens, W. (2004)	Linkage of myostatin pathway genes with knee strength in humans	No eligible target outcome/design. Linkage/genetic association of myostatin-pathway loci and knee strength, not eligible intervention biomarker outcome.
Roth, S.M. (2003)	Myostatin gene expression is reduced in humans with heavy-resistance strength training: A brief communication	No eligible contemporaneous comparator. Only trained-leg muscle biopsies available; report explicitly states muscle samples unavailable from untrained leg.
Carvalho, L.P. (2018)	Myostatin and adipokines: The role of the metabolically unhealthy obese phenotype in muscle function and aerobic capacity in young adults	Observational/nonintervention. Cross-sectional metabolic-obesity phenotypes and biomarker correlations, no longitudinal exercise intervention.
Hansen, J.S. (2016)	Exercise-induced secretion of FGF21 and follistatin are blocked by pancreatic clamp and impaired in type 2 diabetes	Acute/<4-week intervention. Acute exercise with pancreatic-clamp manipulation.
Hjorth, M. (2016)	Myostatin in relation to physical activity and dysglycaemia and its effect on energy metabolism in human skeletal muscle cells	No eligible contemporaneous comparator. Normoglycaemic and dysglycaemic men received the same combined training; metabolic cohorts do not isolate exercise.
Taghibeikzadehbadr, P. (2020)	Effect of different muscle contraction mode on the expression of Myostatin, IGF-1, and PGC-1 alpha family members in human Vastus Lateralis muscle	Acute/<4-week intervention. A biopsy four weeks before a single eccentric/concentric exercise bout is not a four-week training intervention.
Pegoraro, V. (2020)	MyomiRNAs and myostatin as physical rehabilitation biomarkers for myotonic dystrophy	No eligible contemporaneous comparator. Nine DM1 patients assessed pre-post rehabilitation without contemporaneous exercise comparator.
Willis, S.A. (2019)	Effect of exercise intensity on circulating hepatokine concentrations in healthy men	Acute/<4-week intervention. Three eight-hour trials and acute treadmill exercise, not chronic training.
Diaz-Castro, J. (2024)	Assessment of muscle endocrine function and inflammatory signalling in male school children following a physical activity programme	Ineligible population. Male school children; no separately extractable adult sample.
Padrão AI (2015)	Endurance training prevents TWEAK but not myostatin-mediated cardiac remodelling in cancer cachexia.	Nonhuman study. Mammary-tumour animal model; rats, not adult humans.
Martin CI (2005)	The role of myostatin and the calcineurin-signalling pathway in regulating muscle mass in response to exercise training in the rainbow trout Oncorhynchus mykiss Walbaum.	Nonhuman study. Exercise intervention in rainbow trout.
Sandhu, J.S. (2010)	A correlation study between plasma myostatin and peak torque strength gains of bilateral knee extensors and flexors after resistance training in healthy Asian Indians	No eligible contemporaneous comparator. Eighteen subjects all received six-week resistance training; same-subject pre-post design.

Author, year	Report title	Primary reason for exclusion
Sierra, A.P.R. (2023)	Chronic Low or High Nutrient Intake and Myokine Levels	Acute/<4-week intervention. Marathon acute/recovery observations stratified by habitual nutrition; no chronic controlled training contrast.
Sharp, Matthew (2014)	The effects of a myostatin inhibitor on lean body mass, strength, and power in resistance trained males.	Conference abstract/insufficient report. Only conference abstract pages recovered; not an adequate full research report for the prespecified assessment.
Santos, AR (2014)	Blood flow restricted resistance training attenuates myostatin gene expression in a patient with inclusion body myositis	Case report. Single 65-year-old inclusion-body-myositis patient; no concurrent comparator.
Hulmi, JJ (2006)	Resistance Exercise-Induced Myostatin mRNA Expression Before and After a 6-month Strength Training Period	Conference abstract/insufficient report. Only conference abstract pages recovered; not an adequate full research report for the prespecified assessment.
Gray, M (2015)	Effects Of High-velocity Resistance Training On Lean-tissue Mass And Myostatin Among Community-dwelling Older Adults	Conference abstract/insufficient report. Only conference abstract pages recovered; not an adequate full research report for the prespecified assessment.
Bagheri, R (2020)	Comparison of whole egg v. egg white ingestion during 12 weeks of resistance training on skeletal muscle regulatory markers in resistance-trained men	Duplicate report of Bagheri et al. (2020), “Comparison of whole egg v. egg white ingestion during 12 weeks of resistance training on skeletal muscle regulatory markers in resistance-trained men”; the same full-text publication, not an independent report.
Ryan et al., 2013 (duplicate record)	Aerobic Exercise plus Weight Loss Decreases Skeletal Muscle Myostatin Expression and Improves Insulin Sensitivity in Older Adults	Duplicate record of Ryan et al. (2013), DOI 10.1002/oby.20216. Identified after retrieval and counted once among duplicate/overlap exclusions, not again among design/comparator exclusions.
De Sire, A. (2020)	Is myostatin a promising biomarker to monitor muscle modifications in hip fracture patients undergoing therapeutic exercise and amino acids supplementation?	Conference abstract/insufficient report. Only conference abstract pages recovered; not an adequate full research report for the prespecified assessment.
Loenkvist, C (2016)	Progressive resistance training in head and neck cancer patients undergoing concomitant chemoradiotherapy	No eligible contemporaneous comparator. Twelve head-and-neck cancer patients in prospective feasibility training pilot without concurrent control.
McDonald, C. (2015)	Epicatechin enhances mitochondrial biogenesis, increases dystrophin and utrophin, increases follistatin while decreasing myostatin, and improves skeletalmuscle exercise response in adults with Beckermuscular dystrophy (BMD)	Conference abstract/insufficient report. Only conference abstract pages recovered; not an adequate full research report for the prespecified assessment.
Kim, H. (2015)	Exercise and nutrition supplementation on hematological factors in community-dwelling japanese frail elderly women-RCT placebo trial	Conference abstract/insufficient report. Only conference abstract pages recovered; not an adequate full research report for the prespecified assessment.
Dziegala, M.M. (2018)	Iron status and muscle-derived secretion of regenerative and pro-hypertrophic myokines (follistatin and decorin) in men with heart failure with reduced ejection fraction	Conference abstract/insufficient report. Only conference abstract pages recovered; not an adequate full research report for the prespecified assessment.
Willis, S.A. (2018)	The effect of exercise intensity on circulating hepatokine concentrations in healthy men	Conference abstract/insufficient report. Only conference abstract pages recovered; not an adequate full research report for the prespecified assessment.
Glasser, C.E. (2017)	An ongoing phase 2 study evaluating the safety, efficacy, and pharmacokinetics of ACE-083 in patients with CMT1 and CMTX	Conference abstract/insufficient report. Only conference abstract pages recovered; not an adequate full research report for the prespecified assessment.
Vokurková, M. (2023)	The effect of a 24-week training on the production of myokines in patients with idiopathic inflammatory myopathies	Conference abstract/insufficient report. Only conference abstract pages recovered; not an adequate full research report for the prespecified assessment.

Author, year	Report title	Primary reason for exclusion
Tsutsui Y et al., 2024	Impact of electrical muscle stimulation on serum myostatin level and maintenance of skeletal muscle mass in patients undergoing living-donor liver transplantation: Single-center controlled trial.	Acute/<4-week intervention. Target serum MSTN was assessed across preoperative electrical-stimulation exposure; the one-month postoperative CT outcome is not a >=4-week target-marker exercise contrast. Passive EMS is also distinct from active structured exercise.
Ivey FM et al., 2000	Effects of age, gender, and myostatin genotype on the hypertrophic response to heavy resistance strength training.	No eligible target outcome/design. The reported myostatin variable is genotype in age/sex comparisons of training hypertrophy, not longitudinal MSTN/FST concentration or expression.
Vann et al., 2021	Molecular Differences in Skeletal Muscle After 1 Week of Active vs. Passive Recovery From High-Volume Resistance Training	Acute/<4-week intervention. All participants completed the same six-week resistance block, then differed only during a one-week active/passive recovery period. There is no eligible >=4-week controlled exercise contrast.
Li et al., 2025	Efficacy and safety of Shen-Qi Paste, a Traditional Chinese Medicine, in dialysis patients with sarcopenia: A randomized, double-blind, placebo-controlled trial	Nonisolatable exercise effect. Both randomized groups received the same low-intensity exercise; the treatment contrast is Shen-Qi Paste versus placebo.
Primo D et al., 2023	Anthropometric, muscle and serum myokine levels effects of physical exercise with an online platform in female patients with obesity.	No eligible contemporaneous comparator. Twenty-five women underwent the same online training in a single-group pre/post design; no eligible concurrent exercise comparator.
Gorgey AS et al., 2023	Predictors of muscle hypertrophy responsiveness to electrically evoked resistance training after spinal cord injury.	No eligible contemporaneous comparator. Retrospective high-/low-hypertrophy-responder classification after all participants received NMES resistance training; response groups are not randomized or concurrent exercise controls.
Lenk K et al., 2009	Impact of exercise training on myostatin expression in the myocardium and skeletal muscle in a chronic heart failure model.	Nonhuman study. CHF LAD-ligation animal model and C2C12 experiments, not controlled adult human exercise.
Gould et al., 2016	Intramuscular Myostatin Gene Expression following Aerobic and Combined Exercise in Chronic Kidney Disease	Conference abstract/insufficient report. Original target-abstract journal pages and continuation were supplied, not a complete research report. Design, analysis and/or target-marker arm data are insufficient for the full-report assessment; retained for report linkage only.
Zhou et al., 2019	Sarcopenia, muscle mass and plasma myostatin after 12 months of exercise training in patients with CKD: A sub-study of renexc-a randomized controlled trial	Conference abstract/insufficient report. Original target-abstract journal pages and continuation were supplied, not a complete research report. Design, analysis and/or target-marker arm data are insufficient for the full-report assessment; retained for report linkage only.
Dziegala et al., 2018	Muscle-derived follistatin and decorin levels in men with heart failure with reduced ejection fraction and different iron status	Conference abstract/insufficient report. Original target-abstract journal pages and continuation were supplied, not a complete research report. Design, analysis and/or target-marker arm data are insufficient for the full-report assessment; retained for report linkage only.
Lara Vargas et al., 2024	Expression of myostatin and folistatin as a response to high-load strength training to counteract the loss of muscle mass in heart failure with preserved ejection fraction	Conference abstract/insufficient report. Original target-abstract journal pages and continuation were supplied, not a complete research report. Design, analysis and/or target-marker arm data are insufficient for the full-report assessment; retained for report linkage only.
Negaresh et al., 2017	The effect of resistance training on quadriceps muscle volume and some growth factors in elderly and young men	No eligible contemporaneous comparator. Older and younger men all performed the same resistance program; age cohorts do not isolate exercise and there is no nonexercise or differing-training arm.

Author, year	Report title	Primary reason for exclusion
Baghban et al., 2014	Effect of creatine and ginseng supplements on level of the selected growth factors after a period of simulated karate competition in young boys	Nonisolatable exercise effect. All karate groups undertook the competitive/training exposure; randomized differences were creatine, ginseng or placebo, not exercise.
Andonian et al., 2017	Skeletal muscle cytokine and myostatin responses to high-intensity interval training in rheumatoid arthritis contrasted with prediabetes mellitus	Conference abstract/insufficient report. Original target-abstract journal pages and continuation were supplied, not a complete research report. Design, analysis and/or target-marker arm data are insufficient for the full-report assessment; retained for report linkage only.
Shoji et al., 2016	The Increase of Serum Myostatin Level Was Suppressed by Intra-Dialytic Exercise in Hemodialysis Patients	Conference abstract/insufficient report. Original target-abstract journal pages and continuation were supplied, not a complete research report. Design, analysis and/or target-marker arm data are insufficient for the full-report assessment; retained for report linkage only.
Troosters et al., 2010	Resistance training prevents deterioration in quadriceps muscle function during acute exacerbations of chronic obstructive pulmonary disease	Acute or <4 weeks. Randomized resistance exercise during seven-day COPD hospital admission; one-month follow-up does not make the exercise intervention >=4 weeks.
Bazyler et al., 2018	Characteristics of a National Level Female Weightlifter Peaking for Competition: A Case Study	Case report. One national-level female weightlifter followed over 28 weeks; no eligible contemporaneous controlled intervention.
Curiel-Cervantes et al., 2022	Systematic training in master swimmer athletes increases serum insulin growth factor-1 and decreases myostatin and irisin levels	Observational/nonintervention. Habitual systematic versus nonsystematic swimmer groups with cross-sectional marker comparison, not assigned longitudinal controlled training.
Yadav et al., 2026	Impact of structured exercise-based regimen on frailty in pediatric cirrhosis: A randomized controlled trial	Ineligible population. Pediatric cirrhosis trial aged 9–18; no separable eligible adult subgroup established.
Chen et al., 2018	Effect of MSTN rs7570532 polymorphism on body-composition responses to whole-body vibration training in postmenopausal women and underlying molecular mechanisms [title translated from Chinese]	No eligible contemporaneous comparator. All recruited postmenopausal women underwent WBV; genotype groups and plasmid reporter assays do not provide an eligible human controlled exercise contrast.
Bagheri et al., 2021	High-protein Milk Ingestion During A Resistance Training Program Improves Muscular Regulatory Markers In Trained Males.	Conference abstract/insufficient report. Original conference abstract pages supplied, not a complete research report with sufficient eligible design/outcome information. Retain for report linkage only.
Dalbo et al., 2010	Effects Of Different Pre-Exercise Feeding Regimens On Intramuscular Markers Of Myostatin Signaling Following Resistance Training	Conference abstract/insufficient report. Original conference abstract pages supplied, not a complete research report with sufficient eligible design/outcome information. Retain for report linkage only.
Yadav et al., 2025	Effect of Exercise Based Regimen on Frailty in Children with Liver Disease-A Randomized Controlled Trial	Conference abstract/insufficient report. Original conference abstract pages supplied, not a complete research report with sufficient eligible design/outcome information. Retain for report linkage only.
van Bellegem et al., 2022	046 Anorexia nervosa: The influence of underweight on pubertal development and the HPG axis	Conference abstract/insufficient report. Original conference abstract pages supplied, not a complete research report with sufficient eligible design/outcome information. Retain for report linkage only.
Kritikaki et al., 2023	Impaired training-induced myogenesis in COPD is due to blunt expression of extracellular matrix proteins	No eligible canonical MSTN/FST outcome. Five-page conference-author draft contains abstract-submission notes and decorin/SPARC/biglycan results, not measured canonical MSTN/FST outcomes; do not treat references to MSTN inhibition as a measured target outcome.

Author, year	Report title	Primary reason for exclusion
Han et al., 2018	Circulating myostatin level did not change during diet or exercise induced weight loss in type 2 diabetes	Conference abstract/insufficient report. Original conference abstract pages supplied, not a complete research report with sufficient eligible design/outcome information. Retain for report linkage only.
Li et al., 2025	Effects of 12 Weeks of Whole-Body Vibration Training and Vitamin D Supplementation on Bone Density and Muscle Quality in the Aged with Osteosarcopenia: A Randomized Controlled Trial	not structured exercise. Author re-review: whole-body vibration not eligible structured exercise
Barros et al., 2024	Feasibility and Effectiveness of a 12-Week Concurrent Exercise Training on Physical Performance, Muscular Strength, and Myokines in Frail Individuals Living in Nursing Homes: A Cluster Randomized Crossover Trial	Overlapping report. Excluded following author verification of study overlap; no additional independent study was counted.
Mero et al., 2013	Resistance training induced increase in muscle fiber size in young and older men	Overlapping report. Hulmi et al. (2009), "Acute and long-term effects of resistance exercise with or without protein ingestion on muscle hypertrophy and gene expression", was retained as the representative report after author verification.
Hulmi et al., 2009	Resistance exercise with whey protein ingestion affects mTOR signaling pathway and myostatin in men	Overlapping report. Hulmi et al. (2009), "Acute and long-term effects of resistance exercise with or without protein ingestion on muscle hypertrophy and gene expression", was retained as the representative report after author verification.
Huntula et al., 2026	Mechanical loading vs. hypoxic stress: differential regulation of circulating irisin, myostatin, and HIF-1α following resistance training in young adults with overweight	Overlapping report. Excluded following author verification of study overlap; no additional independent study was counted.

Note. One primary reason is assigned per excluded retrieved report (n = 124). The 17 reports not retrieved are not included. Six duplicate or overlapping records were excluded after retrieval and were not counted again as historical database duplicates. Supplied conference reports were assessed rather than automatically classified as unavailable originals.

Supplementary Table S2. Study-level methodological classification and quantitative contribution

Study	Registration	Risk-of-bias tool	Overall judgement	MSTN effects	FST effects	Dose fields available	Synthesis status and reason
Ayaz et al., 2025	NCT05251597	RoB 2	Some concerns	0	1	frequency, duration, session time, sessions	Included in the primary circulating synthesis; comparison-level inputs are provided in Table S3A.
Zhuang et al., 2025	CHICTR2100051178	RoB 2	Some concerns	0	0	No primary dose input	Active WBV-versus-resistance comparison; no non-exercise arm in the submitted primary contrast.
Jeong et al., 2026	NR	RoB 2	Some concerns	2	2	frequency, duration, session time, sessions, total minutes	Included in the primary circulating synthesis; comparison-level inputs are provided in Table S3A.
Zhang et al., 2024	CHICTR2100042803	RoB 2	Some concerns	0	0	No primary dose input	Active resistance/BFR modality comparisons; retained separately, not an exercise-versus-no-exercise effect.
Ataeinosrat et al., 2022	NR	RoB 2	Some concerns	3	3	frequency, duration, session time, sessions, total minutes	Included in the primary circulating synthesis; comparison-level inputs are provided in Table S3A.
Moqaddam et al., 2024	IRCT20151228025732N76	RoB 2	Some concerns	2	2	frequency, duration, session time, sessions, total minutes	Included in the primary circulating synthesis; comparison-level inputs are provided in Table S3A.

Study	Registration	Risk-of-bias tool	Overall judgement	MSTN effects	FST effects	Dose fields available	Synthesis status and reason
Hofmann et al., 2016	NCT01775111	RoB 2	Some concerns	0	0	No primary dose input	Circulating outcomes reported as medians and ranges; arm-level means/SDs were not extracted or imputed.
Seo et al., 2021	NR	RoB 2	Some concerns	1	1	frequency, duration, session time, sessions, total minutes	Circulating MSTN and FST numerical inputs restored from previously extracted group means, SDs and sample sizes.
Saeidi et al., 2023	NR	RoB 2	Some concerns	2	0	frequency, duration, session time, sessions, total minutes	Included in the primary circulating synthesis; comparison-level inputs are provided in Table S3A.
Arazi et al., 2020	NR	RoB 2	Some concerns	2	2	frequency, duration, sessions	Controlled circulating MSTN and FST means/SDs digitized from Figure 3; SD interpretation follows original statistical methods; SEM sensitivity reported.
Mahboobi Semnan et al., 2026	NR	RoB 2	Some concerns	2	2	frequency, duration, session time, sessions, total minutes	Controlled circulating MSTN/FST means and SDs digitized from Figures 3–4; voluntary WB-EMS and resistance arms retained.
Rezaei et al., 2024	IRCT20230727058944N1	RoB 2	Some concerns	1	1	frequency, duration, sessions	Included in the primary circulating synthesis; comparison-level inputs are provided in Table S3A.
Antunes-Correa et al., 2020	NCT01747395	RoB 2	High	0	0	No primary dose input	Skeletal-muscle FST expression, not circulating FST; assess in the separate secondary-outcome synthesis.
Dantas et al., 2020	NCT02441361	RoB 2	Some concerns	0	1	frequency	Circulating FST restored from Table 2 (POST9, n = 31 per arm); muscle *MSTN* transcript/FST protein are reported separately in Table S9.
Shahrokhian et al., 2022	NR	RoB 2	Some concerns	1	0	frequency, duration, sessions	Included in the primary circulating synthesis; comparison-level inputs are provided in Table S3A.
Binns et al., 2017	NR	RoB 2	Some concerns	0	0	No primary dose input	MSTN summaries are presented by sex rather than complete randomized-arm post-training data; compatible randomized-arm mean/SD inputs were not recovered.
Planella-Farrugia et al., 2019	NR	ROBINS-I	Serious	0	0	No primary dose input	MSTN is reported as median/IQR without usable arm SD; no conversion was applied in the submitted synthesis.
Kortas et al., 2020	NR	RoB 2	Some concerns	1	0	frequency, duration, sessions	Included in the primary circulating synthesis; comparison-level inputs are provided in Table S3A.
Zhou et al., 2021	NCT02041156	RoB 2	Some concerns	0	0	No primary dose input	Strength versus balance training with shared exercise exposure; no sedentary comparator for the primary contrast.
Guo et al., 2025	CHICTR2300069599	RoB 2	Some concerns	1	0	frequency, duration, session time, sessions, total minutes	Included in the primary circulating synthesis; comparison-level inputs are provided in Table S3A.
Brooks et al., 2010	NR	RoB 2	Some concerns	0	0	No primary dose input	Muscle expression during bed rest/recovery with amino-acid co-intervention; not a primary circulating effect.

Study	Registration	Risk-of-bias tool	Overall judgement	MSTN effects	FST effects	Dose fields available	Synthesis status and reason
Dawson et al., 2021	NCT01909440	RoB 2	Some concerns	0	0	No primary dose input	Muscle-biopsy MSTN expression, not a circulating concentration outcome.
Ghayomzadeh et al., 2022	IRCT20100601004076N21	RoB 2	Some concerns	1	0	frequency, duration, sessions	Included in the primary circulating synthesis; comparison-level inputs are provided in Table S3A.
Gould et al., 2025	ISRCTN18221837	RoB 2	Some concerns	0	0	No primary dose input	Muscle mRNA acute-bout responses nested in training; not a resting chronic circulating outcome.
Zuo et al., 2023	ChiCTR2200059775 (reported)	RoB 2	Some concerns	0	0	No primary dose input	Functional versus traditional resistance training; both arms trained, so not a non-exercise primary contrast.
Watson et al., 2017	NR	RoB 2	Some concerns	0	0	No primary dose input	Muscle-biopsy mRNA/protein responses, not circulating MSTN/FST concentrations.
Radziminska et al., 2018	NR	RoB 2	Some concerns	0	0	No primary dose input	MSTN reported as medians and quartiles; group means/SDs not available in the extracted dataset.
Razi et al., 2026	NR	RoB 2	Some concerns	2	2	frequency, duration, sessions	Matched exercise-versus-placebo and supplement-matched circulating MSTN/FST comparisons restored from Table 4.
Turon-Skrzypinska et al., 2026	NR	RoB 2	Some concerns	1	0	frequency, session time	Eligible plasma MSTN means, SDs and sample sizes restored from Table II.
Brown et al., 2024	NCT01340300	RoB 2	Some concerns	0	0	No primary dose input	MSTN is eligible, but compatible arm-level SDs were not recovered from adjusted factorial means. FSTL-1 is not classic FST.
Benavente et al., 2024	NR	ROBINS-I	Serious	0	0	No primary dose input	Extracted values describe acute exercise-bout responses nested in training, not a verified resting chronic comparison.
Pazokian et al., 2022	NR	RoB 2	Some concerns	2	2	frequency, duration, sessions	Included in the primary circulating synthesis; comparison-level inputs are provided in Table S3A.
Jang et al., 2016	NR	RoB 2	Some concerns	0	1	duration	Included in the primary circulating synthesis; comparison-level inputs are provided in Table S3A.
Arrieta et al., 2019	Not verified from available report	RoB 2	Some concerns	2	0	6 months; Twice-weekly multicomponent strength, balance and gait training	Two nonoverlapping sex strata clustered within one trial; exact weeks not inferred
Motahari Rad et al., 2023	Not verified from available report	RoB 2	Some concerns	2	2	12 weeks; Concurrent aerobic/resistance in opposite sequences; 3/week	Graphical SD; analyzed rather than randomized *n* used
Bagheri et al., 2019	Not verified from available report	RoB 2	Some concerns	3	3	8 weeks; Upper-, lower-, or combined-body RT; 3/week	Three contrasts share one control; Figure 2 states SD

Study	Registration	Risk-of-bias tool	Overall judgement	MSTN effects	FST effects	Dose fields available	Synthesis status and reason
Bagheri et al., 2020	Not verified from available report	RoB 2	Some concerns	0	0	8 weeks; Endurance then resistance or reverse order; 3/week	Supplied pre-proof has Figure 2 caption but no actual target graph or extractable control dispersion; narrative only
Diel et al., 2010	Not verified from available report	RoB 2	Some concerns	0	2	3 months; Resistance or endurance; 3/week	MYOPRO is propeptide, not circulating mature MSTN; human SD convention differs from animal SEM
Mafi et al., 2019	Not verified from available report	RoB 2	Some concerns	2	2	8 weeks; RT with or without epicatechin; 3/week	Change-score SD; RT vs placebo and RT+EP vs EP only; specimen wording conflict retained
Ghobadi et al., 2024	Not verified from available report	RoB 2	Some concerns	0	0	8 weeks; Autoregulated versus linear periodized resistance exercise; 3/week	Narrative direct-training comparison; no nonexercise arm
Willoughby et al., 2004	Not verified from available report	RoB 2	Some concerns	1	0	12 weeks; Heavy resistance training; 3/week	Serum and muscle levels separated; noncommercial assay sensitivity retained
Koczulla et al., 2020	Not verified from available report	RoB 2	Some concerns	0	0	12 weeks; Whole-body vibration at therapeutic frequency	Direct vibration-dose contrast; not pooled as inactive control
Bagheri et al., 2021	Not verified from available report	RoB 2	Some concerns	1	1	8 weeks; RT+placebo or RT+BCAA; 3/week	RT+placebo comparison retained with uncertainty in banana/dinner matching across groups; exclusion sensitivity reported; Figure 2 SEM converted to SD
Saremi et al., 2010	Not verified from available report	RoB 2	Some concerns	1	0	8 weeks; RT+placebo or RT+creatine; 3/week	Only RT+placebo vs control; creatine arm not isolated
Lenk et al., 2012	Not verified from available report	RoB 2	Some concerns	0	0	12 weeks; Supervised exercise training	Serum numerical data explicitly not shown; secondary muscle outcomes only
Shabkhiz et al., 2021	Not verified from available report	RoB 2	Some concerns	2	0	12 weeks; Resistance training; 3/week	Two nonoverlapping health strata remain one study cluster
Schiffer et al., 2011	Not verified from available report	RoB 2	Some concerns	0	0	12 weeks; Strength or moderate endurance training	No clear chronic group differences; not pooled with blood concentrations
Heisterberg et al., 2018	Not verified from available report	RoB 2	Some concerns	0	0	16 weeks; Heavy RT plus losartan or placebo	Use losartan+RT versus losartan-only to isolate exercise; placebo+RT is not a matched drug comparison
Micielska et al., 2019	Not verified from available report	ROBINS-I	Serious	0	0	5 weeks; High-intensity circuit training; 15 sessions	Not synthesized quantitatively because the target graph did not clearly identify the error-bar definition.
Nilsen TS et al., 2016	Not verified from available report	RoB 2	Some concerns	0	0	16 weeks; Strength training	Secondary outcome; not circulating concentration
Hulmi et al., 2009	Not verified from available report	RoB 2	Some concerns	0	0	21 weeks; RT twice weekly with protein or placebo	Chronic resting and acute biopsy contrasts separated; linked to Hulmi companion report

Study	Registration	Risk-of-bias tool	Overall judgement	MSTN effects	FST effects	Dose fields available	Synthesis status and reason
Lewis et al., 2007	Not verified from available report	RoB 2	Some concerns	0	0	10 weeks; Resistance training with or without testosterone	Hormone cointervention matched; do not mix unmatched testosterone effects
Boeselt et al., 2017	Not verified from available report	ROBINS-I	Serious	1	0	3 months comparable follow-up; Individualized high-intensity aerobic/strength training twice weekly	Use T1; source T2 table conflicts with explicit control follow-up of only 3 months; no 6-month contrast
Santos et al., 2015	Not verified from available report	RoB 2	Some concerns	0	0	8 weeks; Strength versus power resistance-training regimens; 3/week	Narrative tissue outcome; RoB 2 assessment targets randomized power-versus-strength comparison, not the separately recruited control contrast
Lundberg et al., 2013	Not verified from available report	RoB 2	Some concerns	0	0	5 weeks; Unilateral aerobic plus resistance exercise	Paired active comparison; no independent-groups SMD
Laurentino et al., 2012	Not verified from available report	RoB 2	Some concerns	0	0	8 weeks; Low-load RT with blood-flow restriction or high-load RT	Active training contrasts and tissue expression only
Walker et al., 2004	Not verified from available report	RoB 2	Some concerns	0	0	10 weeks; Whole-body resistance training	Active comparator and relative immunoreactivity; narrative only
Irimia et al., 2017	Not verified from available report	ROBINS-I	Serious	0	0	84 days; Flywheel resistance exercise during bed rest	Secondary tissue outcome; no conversion to circulating MSTN
Mendez-Gutierrez A et al., 2023	Not verified from available report	RoB 2	High	0	2	24 weeks; Moderate or vigorous concurrent exercise; 3/week	Figure 3J outcome-specific *n* and SE; chronic MSTN detected in fewer than half and not quantitatively reported
Bozyilan et al., 2026	Not verified from available report	RoB 2	Some concerns	0	0	8 weeks; Added HIIT; usual kickboxing retained in both groups	Active training comparator; narrative only
Strojek et al., 2021	Not verified from available report	RoB 2	Some concerns	0	0	12 weeks; Supervised pelvic-floor muscle training twice weekly	Median/IQR presentation and baseline imbalance; no unplanned conversion to means/SD
Farzanegi et al., 2021	Not verified from available report	RoB 2	Some concerns	3	3	8 weeks; Upper-, lower-, or combined-body resistance training; 3/week	Target post means/SD directly reported in Results; shared control retained
Barati et al., 2025	Not verified from available report	RoB 2	Some concerns	0	0	9 weeks; Linear versus weekly undulating periodized RT	Narrative active-training comparison
Shabani et al., 2018	Not verified from available report	RoB 2	Some concerns	3	0	8 weeks; Resistance, aerobic or combined training; 3/week, 65 min/session	One study contributes both resistance and aerobic/concurrent categories; subgroup study counts are not additive

Study	Registration	Risk-of-bias tool	Overall judgement	MSTN effects	FST effects	Dose fields available	Synthesis status and reason
Karimifard et al., 2023	Not verified from available report	RoB 2	Some concerns	0	0	12 weeks; Rest-pause or traditional resistance training; 3/week	Not synthesized quantitatively because the graph uncertainty type and final control denominator were insufficiently reported.
Bijeh et al., 2022	IRCT20190731044398N3	RoB 2	Some concerns	0	0	12 weeks; RT with/without soy milk; 3 sessions/week	Undefined target-graph error bars; narrative only
Riahy et al., 2024	IRCT20181002041204N2	RoB 2	Some concerns	2	0	12 weeks; HIIT or MICT; 3 sessions/week	Two primary effects; analysis *n* 13/12/12
Niazi et al., 2025	IRCT20190627044037N1	RoB 2	Some concerns	3	3	12 weeks; Circuit RT at 40/60/80% 1RM; 3 sessions/week	Six primary effects; printed means and graph-derived SD
Nasiri et al., 2022	Not verified from available report	RoB 2	Some concerns	1	1	8 weeks; Progressive Pilates; 3 sessions/week	Two primary effects; 11/11 analyzed; vector-graph mean/SD
Watson et al., 2022	Not verified from available report	RoB 2	Some concerns	0	0	12 weeks; Aerobic versus combined aerobic/RT	Independent of Gould; active-training comparison only
Sönmez et al., 2026	Not verified from available report	RoB 2	High	0	0	6 weeks; HIIT versus moderate continuous training	Active-comparator narrative only; no exercise versus nonexercise primary effect
de Souza et al., 2014	Not verified from available report	RoB 2	Some concerns	0	0	8 weeks; Strength, interval or concurrent training	Muscle-level narrative; distinct baseline/training history from Santos
Izaddoust et al., 2017	Not verified from available report	RoB 2; study-family assessment represented by Shabani et al., 2018	Some concerns (study-family assessment under Shabani et al., 2018)	0	0	8 weeks; Resistance training	Companion/subset report of Shabani et al., 2018; no additional independent primary effect. Family-level RoB 2 assessment is reported under Shabani et al., 2018 in Table S8A.
Rasi et al., 2023	Not verified from available report	RoB 2	Some concerns	1	0	12 weeks; Aerobic; 3/week, 45 min	One direct mean/SD primary effect
Hooshmandi et al., 2022	Not verified from available report	RoB 2	Some concerns	0	0	8 weeks; High-intensity interval resistance training	Ratios only; no reconstruction of absolute MSTN
Jazi et al., 2024	Not verified from available report	RoB 2	Some concerns	1	0	12 weeks; Elastic-band resistance training; 3/week	One direct mean/SD effect
Jaafari et al., 2024	Not verified from available report	RoB 2	Some concerns	0	2	8 versus 12 weeks unresolved; Combined training with or without BFR	Two FST effects; MSTN not synthesized because the denominator was unresolved; duration excluded from dose regression.
Marvast et al., 2024	Not verified from available report	RoB 2	Some concerns	1	1	12 weeks; Progressive RT; 2–4/week	Two direct mean/SD effects; source sampling contradiction retained; numerical error convention reviewed
Ramezani et al., 2022	Not verified from available report	RoB 2	Some concerns	3	0	12 weeks; Low/moderate/high interval intensity; 3/week	Three effects share one control and one study cluster

The 79 included reports represented 78 independent study families. Hulmi et al. (2009), “Acute and long-term effects of resistance exercise with or without protein ingestion on muscle hypertrophy and gene expression”, was retained as the representative report from the overlapping Hulmi/Mero report group; Watson and Gould were treated as independent studies. Izaddoust et al. (2017) was

conservatively linked to Shabani et al. (2018) as a companion/subset report using shared anthropometric values and group sizes and did not add an independent primary effect. Review inclusion was distinguished from contribution to each quantitative synthesis (Supplementary Tables S2–S3).

Note. Zuo is represented by the single reported registration identifier ChiCTR2200059775, as stated in the publisher abstract and PubMed; this is not an independently verified registry linkage. Baseline/analyzed totals and trial-versus-biomarker denominator distinctions are reported in main-text Table 1 and its note, without duplicating the sample-flow narrative here. This table supplies registration, risk-of-bias tool allocation, outcome-specific quantitative contribution and dose-field availability. FST, follistatin; MSTN, myostatin; NR, not reported.

Supplementary Table S3A. Effect-size inputs entering the outcome-specific quantitative syntheses

Study and comparison	Outcome	Data form	Intervention n	Comparator n	Hedges' g	Variance	95% CI
Ayaz et al., 2025; E vs C	FST	Post	31	31	0.5573	0.065495	0.06 to 1.06
Kortas et al., 2020; NW vs C	MSTN	Post	18	18	0.7235	0.113925	0.06 to 1.39
Jang et al., 2016; PT vs C	FST	Post	10	10	0.3747	0.187356	-0.47 to 1.22
Rezaei et al., 2024; TST vs C	MSTN	Post	10	9	-0.3191	0.195623	-1.19 to 0.55
Rezaei et al., 2024; TST vs C	FST	Post	10	9	0.6787	0.206175	-0.21 to 1.57
Shahrokhian et al., 2022; Nonlinear RT vs Control	MSTN	Post	10	10	0.9003	0.205972	0.01 to 1.79
Guo et al., 2025; RT vs C	MSTN	Post	13	12	1.6506	0.209092	0.75 to 2.55
Ghayomzadeh et al., 2022; CT vs C	MSTN	Post-Pre change	20	20	1.0063	0.109391	0.36 to 1.65
Jeong et al., 2026; RE vs C †	MSTN	Post	18	18	0.7558	0.114629	0.09 to 1.42
Jeong et al., 2026; RE vs C †	FST	Post	18	18	1.5498	0.141551	0.81 to 2.29
Jeong et al., 2026; REEAA vs EAA †	MSTN	Post	20	20	-0.1030	0.096206	-0.71 to 0.50
Jeong et al., 2026; REEAA vs EAA †	FST	Post	20	20	0.6979	0.102475	0.07 to 1.33
Ataeinosrat et al., 2022; L vs C †	MSTN	Post	11	11	3.4633	0.468131	2.12 to 4.80
Ataeinosrat et al., 2022; L vs C †	FST	Post	11	11	1.9954	0.267813	0.98 to 3.01
Ataeinosrat et al., 2022; M vs C †	MSTN	Post	11	11	3.7957	0.528463	2.37 to 5.22
Ataeinosrat et al., 2022; M vs C †	FST	Post	11	11	3.7187	0.513994	2.31 to 5.12
Ataeinosrat et al., 2022; H vs C †	MSTN	Post	11	11	4.6241	0.702836	2.98 to 6.27
Ataeinosrat et al., 2022; H vs C †	FST	Post	11	11	4.8173	0.748421	3.12 to 6.51
Moqaddam et al., 2024; T vs C †	MSTN	Post	11	11	4.0084	0.569961	2.53 to 5.49

Study and comparison	Outcome	Data form	Intervention n	Comparator n	Hedges' g	Variance	95% CI
Moqaddam et al., 2024; T vs C †	FST	Post	11	11	4.7730	0.737805	3.09 to 6.46
Moqaddam et al., 2024; TS vs S †	MSTN	Post	11	11	4.2495	0.619731	2.71 to 5.79
Moqaddam et al., 2024; TS vs S †	FST	Post	11	11	4.1788	0.604825	2.65 to 5.70
Saeidi et al., 2023; T vs C †	MSTN	Post	15	15	4.0541	0.419712	2.78 to 5.32
Saeidi et al., 2023; TS vs S †	MSTN	Post	15	15	4.0865	0.424427	2.81 to 5.36
Pazokian et al., 2022; FT vs C †	MSTN	Post	10	10	1.2968	0.230173	0.36 to 2.24
Pazokian et al., 2022; FT vs C †	FST	Post	10	10	0.9968	0.211055	0.10 to 1.90
Pazokian et al., 2022; FTBFR vs C †	MSTN	Post	10	10	2.1987	0.317739	1.09 to 3.30
Pazokian et al., 2022; FTBFR vs C †	FST	Post	10	10	0.7672	0.199804	-0.11 to 1.64
Seo et al., 2021; RT vs C	MSTN	Post	12	10	-0.6777	0.181155	-1.51 to 0.16
Seo et al., 2021; RT vs C	FST	Post	12	10	0.5928	0.178460	-0.24 to 1.42
Razi et al., 2026; T vs P	MSTN	Post	11	11	4.8101	0.746704	3.12 to 6.50
Razi et al., 2026; TS vs S	MSTN	Post	11	11	4.4620	0.666006	2.86 to 6.06
Razi et al., 2026; T vs P	FST	Post	11	11	5.4282	0.904919	3.56 to 7.29
Razi et al., 2026; TS vs S	FST	Post	11	11	3.7827	0.525986	2.36 to 5.20
Turon-Skrzypinska et al., 2026; VR vs C	MSTN	Post	39	46	-0.0798	0.046564	-0.50 to 0.34
Mahboobi Semnan et al., 2026; EMS vs C †	MSTN	Post	12	11	2.1824	0.275279	1.15 to 3.21
Mahboobi Semnan et al., 2026; RT vs C †	MSTN	Post	12	11	0.7063	0.173751	-0.11 to 1.52
Mahboobi Semnan et al., 2026; EMS vs C †	FST	Post	12	11	0.2295	0.163128	-0.56 to 1.02
Mahboobi Semnan et al., 2026; RT vs C †	FST	Post	12	11	0.3710	0.165152	-0.43 to 1.17
Arazi et al., 2020; CRT vs C †	MSTN	Post	12	12	1.8981	0.237255	0.94 to 2.85
Arazi et al., 2020; VRT vs C †	MSTN	Post	12	12	1.8898	0.236542	0.94 to 2.84

Study and comparison	Outcome	Data form	Intervention n	Comparator n	Hedges' g	Variance	95% CI
Arazi et al., 2020; CRT vs C †	FST	Post	12	12	2.8403	0.338719	1.70 to 3.98
Arazi et al., 2020; VRT vs C †	FST	Post	12	12	3.0485	0.366588	1.86 to 4.24
Dantas et al., 2020; RYGB + exercise vs RYGB	FST	Post	31	31	0.5993	0.065900	0.10 to 1.10
Arrieta et al., 2019; Women multicomponent vs control	MSTN	Post	28	31	0.0406	0.066202	-0.46 to 0.54
Arrieta et al., 2019; Men multicomponent vs control	MSTN	Post	15	14	-0.2471	0.131591	-0.96 to 0.46
Shabkhiz et al., 2021; Without T2D RT vs control	MSTN	Post	12	12	0.2894	0.157274	-0.49 to 1.07
Shabkhiz et al., 2021; T2D RT vs control	MSTN	Post	10	10	0.4071	0.188059	-0.44 to 1.26
Boeselt et al., 2017; 3-month concurrent vs control	MSTN	Post	20	17	0.3502	0.105928	-0.29 to 0.99
Farzanegi et al., 2021; Upper RT vs control	MSTN	Post	10	10	1.9582	0.289973	0.90 to 3.01
Farzanegi et al., 2021; Lower RT vs control	MSTN	Post	10	10	2.4728	0.353315	1.31 to 3.64
Farzanegi et al., 2021; Combined RT vs control	MSTN	Post	10	10	2.4171	0.345746	1.26 to 3.57
Farzanegi et al., 2021; Upper RT vs control	FST	Post	10	10	1.8943	0.283137	0.85 to 2.94
Farzanegi et al., 2021; Lower RT vs control	FST	Post	10	10	2.3215	0.333165	1.19 to 3.45
Farzanegi et al., 2021; Combined RT vs control	FST	Post	10	10	2.4185	0.345937	1.27 to 3.57
Motahari Rad et al., 2023; A-R concurrent vs control †	MSTN	Post	15	13	0.6755	0.144121	-0.07 to 1.42
Motahari Rad et al., 2023; R-A concurrent vs control †	MSTN	Post	15	13	0.6181	0.142695	-0.12 to 1.36
Motahari Rad et al., 2023; A-R concurrent vs control †	FST	Post	15	13	0.3375	0.137537	-0.39 to 1.06

Study and comparison	Outcome	Data form	Intervention n	Comparator n	Hedges' g	Variance	95% CI
Motahari Rad et al., 2023; R-A concurrent vs control †	FST	Post	15	13	0.4560	0.139346	-0.28 to 1.19
Bagheri et al., 2019; LB RT vs control †	FST	Post	10	10	3.4480	0.513689	2.04 to 4.85
Bagheri et al., 2019; UB RT vs control †	FST	Post	10	10	2.3494	0.336778	1.21 to 3.49
Bagheri et al., 2019; UB+LB RT vs control †	FST	Post	10	10	2.5097	0.358421	1.34 to 3.68
Bagheri et al., 2019; LB RT vs control †	MSTN	Post	10	10	-0.0182	0.183465	-0.86 to 0.82
Bagheri et al., 2019; UB RT vs control †	MSTN	Post	10	10	0.5663	0.192364	-0.29 to 1.43
Bagheri et al., 2019; UB+LB RT vs control †	MSTN	Post	10	10	0.9770	0.209970	0.08 to 1.88
Diel et al., 2010; RT vs control †	FST	Post	10	9	-0.0980	0.192911	-0.96 to 0.76
Diel et al., 2010; Endurance vs control †	FST	Post	8	9	0.1611	0.213575	-0.74 to 1.07
Mafi et al., 2019; RT vs placebo †	MSTN	Change	14	16	1.3664	0.160129	0.58 to 2.15
Mafi et al., 2019; RT+EP vs EP †	MSTN	Change	15	17	0.9753	0.135097	0.25 to 1.70
Mafi et al., 2019; RT vs placebo †	FST	Change	14	16	3.7595	0.379173	2.55 to 4.97
Mafi et al., 2019; RT+EP vs EP †	FST	Change	15	17	2.2014	0.200009	1.32 to 3.08
Willoughby et al., 2004; Heavy RT vs control †	MSTN	Post	12	10	-1.4491	0.222173	-2.37 to -0.53
Bagheri et al., 2021; RT+placebo vs control †	FST	Post	10	10	0.4989	0.190371	-0.36 to 1.35
Bagheri et al., 2021; RT+placebo vs control †	MSTN	Post	10	10	0.2285	0.184906	-0.61 to 1.07
Saremi et al., 2010; RT+placebo vs control †	MSTN	Post	8	8	3.5455	0.672409	1.94 to 5.15
Shabani et al., 2018; Resistance vs control †	MSTN	Post	10	6	0.0160	0.238378	-0.94 to 0.97
Shabani et al., 2018; Aerobic vs control †	MSTN	Post	9	6	-0.4672	0.254455	-1.46 to 0.52

Study and comparison	Outcome	Data form	Intervention n	Comparator n	Hedges' g	Variance	95% CI
Shabani et al., 2018; Concurrent vs control †	MSTN	Post	10	6	0.4458	0.245467	-0.53 to 1.42
Mendez-Gutierrez A et al., 2023; Moderate concurrent vs control †	FST	Post	16	15	0.0343	0.122536	-0.65 to 0.72
Mendez-Gutierrez A et al., 2023; Vigorous concurrent vs control †	FST	Post	16	15	0.3748	0.124937	-0.32 to 1.07
Riahy et al., 2024; HIIT vs Control †	MSTN	Post	13	12	0.9583	0.169827	0.15 to 1.77
Riahy et al., 2024; MICT vs Control †	MSTN	Post	12	12	0.9597	0.176304	0.14 to 1.78
Niazi et al., 2025; LCRT vs Control †	FST	Post	11	11	3.7926	0.527861	2.37 to 5.22
Niazi et al., 2025; MCRT vs Control †	FST	Post	11	11	6.7298	1.300536	4.49 to 8.97
Niazi et al., 2025; HCRT vs Control †	FST	Post	11	11	11.0475	3.219450	7.53 to 14.56
Niazi et al., 2025; LCRT vs Control †	MSTN	Post	11	11	4.4656	0.666812	2.87 to 6.07
Niazi et al., 2025; MCRT vs Control †	MSTN	Post	11	11	4.5562	0.687243	2.93 to 6.18
Niazi et al., 2025; HCRT vs Control †	MSTN	Post	11	11	4.5512	0.686099	2.93 to 6.17
Nasiri et al., 2022; Pilates vs Control †	MSTN	Post	11	11	-0.7588	0.182666	-1.60 to 0.08
Nasiri et al., 2022; Pilates vs Control †	FST	Post	11	11	0.2622	0.169990	-0.55 to 1.07
Rasi et al., 2023; Aerobic vs Control	MSTN	Post	10	10	3.7002	0.563783	2.23 to 5.17
Azamian Jazi et al., 2024; Elastic-band RT vs Control	MSTN	Post	14	14	1.1923	0.161995	0.40 to 1.98
Barzegari Marvast et al., 2024; RT vs Control	MSTN	Post	15	15	0.7198	0.135475	-0.00 to 1.44
Barzegari Marvast et al., 2024; RT vs Control	FST	Post	15	15	1.3540	0.158963	0.57 to 2.14

Study and comparison	Outcome	Data form	Intervention n	Comparator n	Hedges' g	Variance	95% CI
Ramezani et al., 2022; Low intensity vs Control	MSTN	Post	12	11	-0.3387	0.164606	-1.13 to 0.46
Ramezani et al., 2022; Moderate intensity vs Control	MSTN	Post	12	11	0.5131	0.168142	-0.29 to 1.32
Ramezani et al., 2022; High intensity vs Control	MSTN	Post	12	11	-0.9117	0.181666	-1.75 to -0.08
Jaafari et al., 2024; Combined+BFR vs Control	FST	Post	15	15	1.6604	0.175457	0.84 to 2.48
Jaafari et al., 2024; Combined vs Control	FST	Post	15	15	1.2453	0.153917	0.48 to 2.01

Positive g denotes lower MSTN or higher FST, not benefit. †Figure-derived estimate. All 99 comparison effects are retained; 63 use graph-derived summaries (28 original, 35 recovered). The nine directly reported mean/SD effects added in the latest stage were reviewed by CQ and CY. Shared controls and repeated outcomes remain within study clusters; source inconsistencies are not resolved by guessing denominators.

Supplementary Table S3A (continued). Assay and postexercise sampling metadata

Rows group comparisons only when study, outcome, assay and sampling conditions are identical; every current primary effect is mapped. Extraction labels distinguish figure-derived values from pre-existing numerical entries. Timing refers to the blood-specific statement where available. Conflicting general or non-blood timing is retained rather than silently resolved.

Study, outcome and comparisons	Extraction	Assay platform / manufacturer / kit	Postexercise interval / fasting / source caveat	PDF source
Ayaz et al., 2025 FST; E vs C	Reported numerical data	ELISA; Sunred; catalogue NR	24 h after last session; 8 h overnight fast	pp. 3, 4
Kortas et al., 2020 MSTN; NW vs C	Reported numerical data	ELISA; R&D Systems; DGDF80	1 day after training programme; Overnight fast Do not convert programme-end wording to an exact timed interval.	pp. 4, 5
Jang et al., 2016 FST; PT vs C	Reported numerical data	ELISA; absorbance 450 nm; reader model NR; R&D Systems; catalogue NR	NR; 12 h overnight fast Avoidance of excessive activity the previous day is not a stated last-session interval.	pp. 2
Rezaei et al., 2024 MSTN; TST vs C	Reported numerical data	ELISA; ZellBio; ZB-0403-H9648	48 h after last session; 12 h in blood Methods; 8 h in Figure 1 Source fasting-duration discrepancy retained.	pp. 4, 5
Rezaei et al., 2024 FST; TST vs C	Reported numerical data	ELISA; ZellBio; ZB-11010 S-H9648 (printed ZB-11,010 S-H9648)	48 h after last session; 12 h in blood Methods; 8 h in Figure 1 Source fasting-duration discrepancy retained.	pp. 4, 5
Shahrokhian et al., 2022 MSTN; Nonlinear RT vs Control	Reported numerical data	NR for MSTN; NR; ZellBio kit described for insulin, not MSTN	48 h after last session; 8–10 h overnight fast Do not assign the insulin ELISA kit to MSTN.	pp. 3
Guo et al., 2025 MSTN; RT vs C	Reported numerical data	ELISA; BioTek SYNERGY H1 reader; Jianglai Biotechnology; catalogue NR	NR; 12 h overnight fast Pre/post-intervention sampling is stated without an explicit final-session interval.	pp. 5
Ghayomzadeh et al., 2022 MSTN; CT vs C	Reported numerical data	ELISA; R&D Systems; catalogue NR	At least 48 h after last session; Overnight fast Separate denominator issue: 20/20 randomized; 18/19 completed; published change-score denominator needs resolution.	pp. 3, 4

Study, outcome and comparisons	Extraction	Assay platform / manufacturer / kit	Postexercise interval / fasting / source caveat	PDF source
Jeong et al., 2026<br>MSTN; RE vs C, REEAA vs EAA	Figure: original	DuoSet ELISA; R&D Systems; DY788-05	At least 48 h after last exercise and EAA intake; 12 h; blood drawn at 08:00 96 enrolled/randomized; 76 completed (CON/RE/EAA/RE+EAA: 18/18/20/20).	pp. 3, 4, 5
Jeong et al., 2026<br>FST; RE vs C, REEAA vs EAA	Figure: original	DuoSet ELISA; R&D Systems; DY669	At least 48 h after last exercise and EAA intake; 12 h; blood drawn at 08:00 96 enrolled/randomized; 76 completed (CON/RE/EAA/RE+EAA: 18/18/20/20).	pp. 3, 4, 5
Ataeinosrat et al., 2022<br>MSTN; L vs C, M vs C, H vs C	Figure: original	ELISA; R&D Systems; DGDF80	48 h after last session; 12 h overnight fast	pp. 4
Ataeinosrat et al., 2022<br>FST; L vs C, M vs C, H vs C	Figure: original	ELISA; R&D Systems; DFN00	48 h after last session; 12 h overnight fast	pp. 4
Moqaddam et al., 2024<br>MSTN; T vs C, TS vs S	Figure: original	ELISA; R&D Systems (Boston Biochem as printed); catalogue NR	72 h in blood Methods; 48 h for general posttests; Fasting; exact fasting-duration sentence ambiguous Blood-specific 72 h retained alongside conflicting general 48 h; 60 randomized in abstract versus 68 in Methods/results; 44 completers.	pp. 1, 4, 6, 7
Moqaddam et al., 2024<br>FST; T vs C, TS vs S	Figure: original	ELISA; R&D Systems (Boston Biochem as printed); catalogue NR	72 h in blood Methods; 48 h for general posttests; Fasting; exact fasting-duration sentence ambiguous Blood-specific 72 h retained alongside conflicting general 48 h; 60 randomized in abstract versus 68 in Methods/results; 44 completers.	pp. 1, 4, 6, 7
Saeidi et al., 2023<br>MSTN; T vs C, TS vs S	Figure: original	ELISA; R&D Systems; DGDF80	72 h in blood Methods; 48 h for general posttests; Fasting; 08:00–10:00; duration NR Blood-specific 72 h retained; general measurement section states 48 h.	pp. 3, 4
Pazokian et al., 2022<br>MSTN; FT vs C, FTBFR vs C	Figure: original	ELISA; Cusabio; CSB-E11300h	48 h after 6-week intervention; 8–10 h overnight fast EDTA tube and serum wording conflict in source; no reclassification assumed.	pp. 4
Pazokian et al., 2022<br>FST; FT vs C, FTBFR vs C	Figure: original	ELISA; Cusabio; CSB-E-13516h (as printed)	48 h after 6-week intervention; 8–10 h overnight fast EDTA tube and serum wording conflict in source; no reclassification assumed.	pp. 4
Seo et al., 2021<br>MSTN; RT vs C	Reported numerical data	ELISA; VERSA Max reader; R&D Systems Myostatin Immunoassay; catalogue NR	At least 48 h without vigorous activity; exact last-session interval NR; 12 h; 08:00–09:00 Exercise-abstention instruction is distinguished from a directly timed final-session interval.	pp. 5
Seo et al., 2021<br>FST; RT vs C	Reported numerical data	ELISA; VERSA Max reader; R&D Systems Human Follistatin Immunoassay; catalogue NR	At least 48 h without vigorous activity; exact last-session interval NR; 12 h; 08:00–09:00 Exercise-abstention instruction is distinguished from a directly timed final-session interval.	pp. 5
Razi et al., 2026<br>MSTN; T vs P, TS vs S	Reported numerical data	ELISA; R&D Systems; DGDF80	48 h after final session; 12 h overnight fast; 08:00–10:00	pp. 8
Razi et al., 2026<br>FST; T vs P, TS vs S	Reported numerical data	ELISA; R&D Systems; DFN00	48 h after final session; 12 h overnight fast; 08:00–10:00	pp. 8
Turon-Skrzypinska et al., 2026<br>MSTN; VR vs C	Reported numerical data	ELISA; absorbance 450 nm; Envision analysis; SunRed Human MSTN test; catalogue NR	NR; sampled before hemodialysis at 3 months; NR Dialysis-relative timing is reported; it is not an exercise-session interval.	pp. 4
Mahboobi Semnan et al., 2026<br>MSTN; EMS vs C, RT vs C	Figure: revision	ELISA; ZellBio; RK01885	Blood-specific interval NR; Fasting instructed in assessment protocol; duration NR 48 h strenuous-activity avoidance is specified in anthropometric methods, not as a confirmed blood-sampling interval.	pp. 5

Study, outcome and comparisons	Extraction	Assay platform / manufacturer / kit	Postexercise interval / fasting / source caveat	PDF source
Mahboobi Semnan et al., 2026<br>FST; EMS vs C, RT vs C	Figure: revision	ELISA; ZellBio; RK00289	Blood-specific interval NR; Fasting instructed in assessment protocol; duration NR 48 h strenuous-activity avoidance is specified in anthropometric methods, not as a confirmed blood-sampling interval.	pp. 5
Arazi et al., 2020<br>MSTN; CRT vs C, VRT vs C	Figure: revision	ELISA; Shanghai Crystal Day Biotech; catalogue NR	48 h after last session; 12 h fast; 8 h sleep	pp. 4
Arazi et al., 2020<br>FST; CRT vs C, VRT vs C	Figure: revision	ELISA; Shanghai Crystal Day Biotech; catalogue NR	48 h after last session; 12 h fast; 8 h sleep	pp. 4
Dantas et al., 2020<br>FST; RYGB + exercise vs RYGB	Reported numerical data	ELISA; Thermo Fisher Scientific; catalogue NR	FST-specific interval NR; 72–96 h stated for OGTT; Fasting blood chemistry Do not automatically assign the OGTT interval to the FST specimen.	pp. 2, 3
Arrieta, H. (2019)	Reported numerical data	Serum; ELISA; R&D Systems GDF-8/myostatin Quantikine; acid activation	Interval after last exercise session NR; fasting morning draw. Total immunoreactive MSTN after activation, not free active ligand	Arrieta et al., 2019; full report, PDF pp. 1–11
Motahari Rad, M (2023)	Figure-derived data	Serum; ELISA; ZellBio human MSTN and human FST kits	48 h; 12-h overnight fast. Measured/analyzed *n* 15/15/13, not randomized 17/group	Motahari Rad et al., 2023; full report, PDF pp. 1–11
Bagheri, R. (2019)	Figure-derived data	Serum; ELISA; Glory Science Co., human myostatin/follistatin	48 h after final session; fasting. Target Figure 2 SD; no inference of receptor bioactivity	Bagheri et al., 2019; full report, PDF pp. 1–24
Diel, P. (2010)	Figure-derived data	Serum; Immuno-PCR Imperacer; FST antibodies AF669/BAF669 (R&D); human FST standard	At least 2 days rest stated for testing/biopsies; fasting blood. Human data are mean/SD; animal tissue SEM convention not applied; MYOPRO excluded from MSTN	Diel et al., 2010; full report, PDF pp. 1–9
Mafi, F (2019)	Figure-derived data	Plasma (Methods); serum in figure caption; FST ELISA R&D Systems; MSTN ELISA Immundiagnostik AG	48 h; 10-12-h overnight fast. Specimen-label inconsistency retained; source post values corrected for plasma-volume change; change SD not post SD	Mafi et al., 2019; full report, PDF pp. 1–27
Willoughby, D.S. (2004)	Figure-derived data	Serum; Noncommercial antibody-based immunoassay; Santa Cruz reagents	Blood immediately before and after test sessions; target Figure 4C does not separate them. Acute-on-chronic timing ambiguity; exclude-report sensitivity, not silently described as a resting ELISA estimate	Willoughby et al., 2004; full report, PDF pp. 1–9
Bagheri, R. (2021)	Figure-derived data	Serum; ELISA; Zellbio human MSTN/FST	Exact postexercise interval NR in inspected passage; sampling Methods retained. Figure 2 SEM; peri-training banana/dinner provision versus control not explicitly matched; eligibility and cointervention sensitivity flagged	Bagheri et al., 2021; full report, PDF pp. 1–7

Study, outcome and comparisons	Extraction	Assay platform / manufacturer / kit	Postexercise interval / fasting / source caveat	PDF source
Saremi, A. (2010)	Figure-derived data	Serum; In-house competitive ELISA; Santa Cruz SC-6884P standard and SC-6884 antibody	48 h after final session. Not a named commercial sandwich-ELISA kit; creatine group not used	Saremi et al., 2010; full report, PDF pp. 1–6
Shabkhiz, F (2021)	Reported numerical data	Serum; ELISA; R&D Systems GDF-8/myostatin kit	48 h; 10-h overnight fast. Separate T2D/non-T2D strata share one study cluster	Shabkhiz et al., 2021; full report, PDF pp. 1–15
Boeselt, T. (2017)	Reported numerical data	Serum; Quantikine ELISA; R&D Systems/Bio-Techne; dilution 1:4	Interval after final session NR. Use 3-month T1 only; no verified comparable 6-month control exposure	Boeselt et al., 2017; full report, PDF pp. 1–10
Farzanegi, P. (2021)	Reported numerical data	Serum; ELISA; Cusabio, China; catalog NR	48 h after final exercise session. Post means and SD in Results, not reconstructed from pre-post significance tests	Farzanegi et al., 2021; full report, PDF pp. 1–6
Mendez-Gutierrez A (2023)	Figure-derived data	Plasma; Immunoassay panel; target kit details referred to source supplementary methods	4-5 days; overnight fast. Figure 3J SE; outcome-specific *n* 15/16/16. Out-of-range concentrations excluded by original investigators; subset-selection limitation	Mendez-Gutierrez A (2023) et al., 2023; full report, PDF pp. 1–14
Shabani, Ramin (2018)	Figure-derived data	Serum; ELISA; ZellBio human myostatin	At least 48 h without strenuous exercise; fasting sampling. Graph SD; outcome-specific group sizes 10/9/10/6	Shabani et al., 2018; full report, PDF pp. 1–9
Riahy et al., 2024	Graph-derived mean/SD	Serum MSTN; ZellBio ELISA; 48 h postexercise	48 h; fasting	Riahy et al., 2024; full report, PDF pp. 1–13
Niazi et al., 2025	Graph-derived SD; Niazi means directly printed	Serum MSTN/FST; R&D ELISA	Fasting reported; exact interval after the final exercise session NR.	Niazi et al., 2025; full report, PDF pp. 1–11
Nasiri et al., 2022	Graph-derived mean/SD	Serum MSTN/FST; Figure 2	Exact postexercise sampling interval NR in the source information available for extraction.	Nasiri et al., 2022; full report, PDF pp. 1–9

Study, outcome and comparisons	Extraction	Assay platform / manufacturer / kit	Postexercise interval / fasting / source caveat	PDF source
Rasi et al., 2023	Reported numerical data	Serum MSTN; 48 h	One direct mean/SD primary effect	Rasi et al., 2023; Table 1; PDF p. 6
Jazi et al., 2024	Reported numerical data	Serum MSTN; 48 h	One direct mean/SD effect	Jazi et al., 2024; Table 2; PDF p. 8
Jaafari et al., 2024	Reported numerical data	Serum FST; MSTN denominator unresolved	Two FST effects; MSTN not synthesized because the denominator was unresolved; duration excluded from dose regression.	Jaafari et al., 2024; Table 3; PDF p. 7
Marvast et al., 2024	Reported numerical data	Serum MSTN/FST; 24/48 h conflict	Two direct mean/SD effects; source sampling contradiction retained; numerical error convention reviewed	Marvast et al., 2024; Table 3; PDF p. 4
Ramezani et al., 2022	Reported numerical data	Serum MSTN; 48 h	Three effects share one control and one study cluster	Ramezani et al., 2022; Table 2; PDF p. 5

NR, not reported or not sufficiently specified for the target marker. A catalogue for another analyte was not assigned to MSTN/FST. Source pages refer to the archived report PDF; report-level source locations and comparison-level mappings are supplied in File S2. Kit catalogue strings are reported as printed. Trial totals are reported in main-text Table 1, whereas outcome-specific input denominators are reported in Table S3A. Ghayomzadeh denominator sensitivities are reported in Table S4.

Supplementary Table S3B. Dose fields linked to each quantitative comparison

Study and comparison	Outcome	Frequency (sessions/week)	Duration (weeks)	Session duration (min)	Total sessions	Total exercise (min)
Ayaz et al., 2025; E vs C	FST	3	12	50	36	NR
Kortas et al., 2020; NW vs C	MSTN	3	12	NR	36	NR
Jang et al., 2016; PT vs C	FST	NR	8	NR	NR	NR
Rezaei et al., 2024; TST vs C	MSTN	3	8	NR	24	NR
Rezaei et al., 2024; TST vs C	FST	3	8	NR	24	NR
Shahrokhian et al., 2022; Nonlinear RT vs Control	MSTN	3	12	NR	36	NR
Guo et al., 2025; RT vs C	MSTN	3	36	40	108	4320
Ghayomzadeh et al., 2022; CT vs C	MSTN	3	26	NR	78	NR
Jeong et al., 2026; RE vs C	MSTN	3	12	60	36	2160
Jeong et al., 2026; RE vs C	FST	3	12	60	36	2160
Jeong et al., 2026; REEAA vs EAA	MSTN	3	12	60	36	2160
Jeong et al., 2026; REEAA vs EAA	FST	3	12	60	36	2160

Study and comparison	Outcome	Frequency (sessions/week)	Duration (weeks)	Session duration (min)	Total sessions	Total exercise (min)
Ataeinosrat et al., 2022; L vs C	MSTN	3	12	70	36	2520
Ataeinosrat et al., 2022; L vs C	FST	3	12	70	36	2520
Ataeinosrat et al., 2022; M vs C	MSTN	3	12	70	36	2520
Ataeinosrat et al., 2022; M vs C	FST	3	12	70	36	2520
Ataeinosrat et al., 2022; H vs C	MSTN	3	12	70	36	2520
Ataeinosrat et al., 2022; H vs C	FST	3	12	70	36	2520
Moqaddam et al., 2024; T vs C	MSTN	3	12	60	36	2160
Moqaddam et al., 2024; T vs C	FST	3	12	60	36	2160
Moqaddam et al., 2024; TS vs S	MSTN	3	12	60	36	2160
Moqaddam et al., 2024; TS vs S	FST	3	12	60	36	2160
Saeidi et al., 2023; T vs C	MSTN	3	12	60	36	2160
Saeidi et al., 2023; TS vs S	MSTN	3	12	60	36	2160
Pazokian et al., 2022; FT vs C	MSTN	3	6	NR	18	NR
Pazokian et al., 2022; FT vs C	FST	3	6	NR	18	NR
Pazokian et al., 2022; FTBFR vs C	MSTN	3	6	NR	18	NR
Pazokian et al., 2022; FTBFR vs C	FST	3	6	NR	18	NR
Seo et al., 2021; RT vs C	MSTN	3	16	60	48	2880
Seo et al., 2021; RT vs C	FST	3	16	60	48	2880
Razi et al., 2026; T vs P	MSTN	3	12	NR	36	NR
Razi et al., 2026; TS vs S	MSTN	3	12	NR	36	NR
Razi et al., 2026; T vs P	FST	3	12	NR	36	NR
Razi et al., 2026; TS vs S	FST	3	12	NR	36	NR
Turon-Skrzypinska et al., 2026; VR vs C	MSTN	3	NR	20	NR	NR
Mahboobi Semnan et al., 2026; EMS vs C	MSTN	2	8	20	16	320
Mahboobi Semnan et al., 2026; RT vs C	MSTN	2	8	20	16	320

Study and comparison	Outcome	Frequency (sessions/week)	Duration (weeks)	Session duration (min)	Total sessions	Total exercise (min)
Mahboobi Semnan et al., 2026; EMS vs C	FST	2	8	20	16	320
Mahboobi Semnan et al., 2026; RT vs C	FST	2	8	20	16	320
Arazi et al., 2020; CRT vs C	MSTN	3	8	NR	24	NR
Arazi et al., 2020; VRT vs C	MSTN	3	8	NR	24	NR
Arazi et al., 2020; CRT vs C	FST	3	8	NR	24	NR
Arazi et al., 2020; VRT vs C	FST	3	8	NR	24	NR
Dantas et al., 2020; RYGB + exercise vs RYGB	FST	3	NR	NR	NR	NR
Arrieta et al., 2019; Women multicomponent vs control	MSTN	2	NR	NR	NR	NR
Arrieta et al., 2019; Men multicomponent vs control	MSTN	2	NR	NR	NR	NR
Shabkhiz et al., 2021; Without T2D RT vs control	MSTN	3	12	NR	36	NR
Shabkhiz et al., 2021; T2D RT vs control	MSTN	3	12	NR	36	NR
Boeselt et al., 2017; 3-month concurrent vs control	MSTN	2	NR	90	NR	NR
Farzanegi et al., 2021; Upper RT vs control	MSTN	3	8	NR	24	NR
Farzanegi et al., 2021; Lower RT vs control	MSTN	3	8	NR	24	NR
Farzanegi et al., 2021; Combined RT vs control	MSTN	3	8	NR	24	NR
Farzanegi et al., 2021; Upper RT vs control	FST	3	8	NR	24	NR
Farzanegi et al., 2021; Lower RT vs control	FST	3	8	NR	24	NR
Farzanegi et al., 2021; Combined RT vs control	FST	3	8	NR	24	NR
Motahari Rad et al., 2023; A-R concurrent vs control	MSTN	3	12	NR	36	NR

Study and comparison	Outcome	Frequency (sessions/week)	Duration (weeks)	Session duration (min)	Total sessions	Total exercise (min)
Motahari Rad et al., 2023; R-A concurrent vs control	MSTN	3	12	NR	36	NR
Motahari Rad et al., 2023; A-R concurrent vs control	FST	3	12	NR	36	NR
Motahari Rad et al., 2023; R-A concurrent vs control	FST	3	12	NR	36	NR
Bagheri et al., 2019; LB RT vs control	FST	3	8	NR	24	NR
Bagheri et al., 2019; UB RT vs control	FST	3	8	NR	24	NR
Bagheri et al., 2019; UB+LB RT vs control	FST	3	8	NR	24	NR
Bagheri et al., 2019; LB RT vs control	MSTN	3	8	NR	24	NR
Bagheri et al., 2019; UB RT vs control	MSTN	3	8	NR	24	NR
Bagheri et al., 2019; UB+LB RT vs control	MSTN	3	8	NR	24	NR
Diel et al., 2010; RT vs control	FST	3	NR	NR	NR	NR
Diel et al., 2010; Endurance vs control	FST	3	NR	NR	NR	NR
Mafi et al., 2019; RT vs placebo	MSTN	3	8	NR	24	NR
Mafi et al., 2019; RT+EP vs EP	MSTN	3	8	NR	24	NR
Mafi et al., 2019; RT vs placebo	FST	3	8	NR	24	NR
Mafi et al., 2019; RT+EP vs EP	FST	3	8	NR	24	NR
Willoughby et al., 2004; Heavy RT vs control	MSTN	3	12	NR	36	NR
Bagheri et al., 2021; RT+placebo vs control	FST	3	8	NR	24	NR
Bagheri et al., 2021; RT+placebo vs control	MSTN	3	8	NR	24	NR
Saremi et al., 2010; RT+placebo vs control	MSTN	3	8	NR	24	NR
Shabani et al., 2018; Resistance vs control	MSTN	3	8	65	24	1560
Shabani et al., 2018; Aerobic vs control	MSTN	3	8	65	24	1560
Shabani et al., 2018; Concurrent vs control	MSTN	3	8	65	24	1560

Study and comparison	Outcome	Frequency (sessions/week)	Duration (weeks)	Session duration (min)	Total sessions	Total exercise (min)
Mendez-Gutierrez A et al., 2023; Moderate concurrent vs control	FST	3	24	NR	72	NR
Mendez-Gutierrez A et al., 2023; Vigorous concurrent vs control	FST	3	24	NR	72	NR
Riahy et al., 2024; HIIT vs Control	MSTN	3	12	NR	36	NR
Riahy et al., 2024; MICT vs Control	MSTN	3	12	NR	36	NR
Niazi et al., 2025; LCRT vs Control	FST	3	12	NR	36	NR
Niazi et al., 2025; MCRT vs Control	FST	3	12	NR	36	NR
Niazi et al., 2025; HCRT vs Control	FST	3	12	NR	36	NR
Niazi et al., 2025; LCRT vs Control	MSTN	3	12	NR	36	NR
Niazi et al., 2025; MCRT vs Control	MSTN	3	12	NR	36	NR
Niazi et al., 2025; HCRT vs Control	MSTN	3	12	NR	36	NR
Nasiri et al., 2022; Pilates vs Control	MSTN	3	8	NR	24	NR
Nasiri et al., 2022; Pilates vs Control	FST	3	8	NR	24	NR
Rasi et al., 2023; Aerobic vs Control	MSTN	3	12	45	36	NR
Azamian Jazi et al., 2024; Elastic-band RT vs Control	MSTN	3	12	NR	36	NR
Barzegari Marvast et al., 2024; RT vs Control	MSTN	NR	12	NR	36	NR
Barzegari Marvast et al., 2024; RT vs Control	FST	NR	12	NR	36	NR
Ramezani et al., 2022; Low intensity vs Control	MSTN	3	12	NR	36	NR
Ramezani et al., 2022; Moderate intensity vs Control	MSTN	3	12	NR	36	NR
Ramezani et al., 2022; High intensity vs Control	MSTN	3	12	NR	36	NR
Jaafari et al., 2024; Combined+BFR vs Control	FST	3	NR	NR	NR	NR
Jaafari et al., 2024; Combined vs Control	FST	3	NR	NR	NR	NR

Note. NR indicates that the dose component could not be confirmed from the report. Dose fields were used only when prespecified coverage and variation criteria were met.

Supplementary Table S4. Sensitivity and leave-one-study-out analyses

Outcome	Analysis	Effects	Hedges' g	95% CI	P value	τ²	I² (%)
MSTN	Within-study aggregation rho=0	33	1.25	0.67 to 1.83	<0.001	2.4184	95.52
MSTN	Within-study aggregation rho=0.5	33	1.21	0.64 to 1.78	<0.001	2.2446	93.68
MSTN	Within-study aggregation rho=0.8	33	1.15	0.59 to 1.71	<0.001	2.0976	92.68
MSTN	Exclude |g| > 3	43	0.61	0.34 to 0.89	<0.001	0.5796	78.36
FST	Within-study aggregation rho=0	23	1.57	0.91 to 2.23	<0.001	2.0891	94.76
FST	Within-study aggregation rho=0.5	23	1.39	0.83 to 1.95	<0.001	1.3624	89.92
FST	Within-study aggregation rho=0.8	23	1.15	0.69 to 1.60	<0.001	0.7754	82.38
FST	Exclude |g| > 3	30	1.00	0.69 to 1.32	<0.001	0.4587	73.40
MSTN	Exclude newly recovered reports	24	1.66	0.72 to 2.60	0.002	2.6547; 0.1214	—
MSTN	Newly recovered reports only	33	0.92	0.18 to 1.65	0.018	2.0362; 0.0000	—
MSTN	Exclude all graph-derived estimates	23	0.94	0.22 to 1.66	0.014	1.6620; 0.0154	—
MSTN	Exclude change-score effects	54	1.27	0.65 to 1.88	<0.001	2.5886; 0.0331	—
MSTN	Exclude noncommercial Willoughby assay	56	1.33	0.77 to 1.89	<0.001	2.2555; 0.0258	—
MSTN	Exclude nonrandomized comparisons	56	1.28	0.69 to 1.87	<0.001	2.4683; 0.0266	—
MSTN	Exclude |g| > 3	43	0.56	0.22 to 0.90	0.002	0.5546; 0.0434	—
MSTN	Exclude latest recovery-stage increment	51	1.25	0.63 to 1.87	<0.001	2.5301; 0.0000	—
MSTN	Exclude Jaafari et al., 2024	57	1.25	0.68 to 1.82	<0.001	2.4053; 0.0262	—
MSTN	Exclude Niazi et al., 2025	54	1.15	0.60 to 1.69	<0.001	2.1138; 0.0319	—
MSTN	Arazi plotted error interpreted as SEM	57	1.21	0.64 to 1.78	<0.001	2.4031; 0.0232	—
MSTN	Exclude newly recovered noncommercial assay reports	55	1.27	0.71 to 1.83	<0.001	2.2011; 0.0253	—

Outcome	Analysis	Effects	Hedges' g	95% CI	P value	τ^2	I^2 (%)
MSTN	Exclude Bagheri 2021 with unmatched feeding documentation	56	1.28	0.70 to 1.87	<0.001	2.4596; 0.0265	—
MSTN	Exclude Ghayomzadeh change-score effect only	56	1.26	0.67 to 1.85	<0.001	2.4940; 0.0266	—
MSTN	Ghayomzadeh denominator sensitivity *n*=18/19	57	1.25	0.68 to 1.82	<0.001	2.4057; 0.0262	—
FST	Exclude newly recovered reports	20	1.64	0.65 to 2.63	0.004	2.1607; 0.1561	—
FST	Newly recovered reports only	22	1.52	0.48 to 2.56	0.009	2.2239; 0.0000	—
FST	Exclude all graph-derived estimates	13	1.31	0.41 to 2.22	0.010	1.2449; 0.0000	—
FST	Exclude change-score effects	40	1.51	0.84 to 2.18	<0.001	2.1261; 0.0029	—
FST	Exclude ACTIBATE detection-limited subset	40	1.65	0.97 to 2.33	<0.001	2.1227; 0.0763	—
FST	Exclude $\|g\| > 3$	30	1.00	0.62 to 1.37	<0.001	0.5061; 0.0000	—
FST	Exclude latest recovery-stage increment	39	1.61	0.88 to 2.33	<0.001	2.3518; 0.0726	—
FST	Exclude Jaafari et al., 2024	40	1.59	0.90 to 2.28	<0.001	2.2257; 0.0677	—
FST	Exclude Niazi et al., 2025	39	1.40	0.83 to 1.98	<0.001	1.5563; 0.0069	—
FST	Arazi plotted error interpreted as SEM	42	1.48	0.84 to 2.12	<0.001	2.0127; 0.0266	—
FST	Exclude newly recovered noncommercial assay reports	40	1.66	0.98 to 2.33	<0.001	2.0952; 0.0720	—
FST	Exclude Bagheri 2021 with unmatched feeding documentation	41	1.63	0.95 to 2.31	<0.001	2.1594; 0.0472	—
MSTN	Multilevel CR2 leave out Kortas et al., 2020	56	1.27	0.68 to 1.86	<0.001	—	—
MSTN	Multilevel CR2 leave out Rezaei et al., 2024	56	1.30	0.72 to 1.88	<0.001	—	—
MSTN	Multilevel CR2 leave out Shahrokhian et al., 2022	56	1.26	0.67 to 1.85	<0.001	—	—

Outcome	Analysis	Effects	Hedges' g	95% CI	P value	τ²	I² (%)
MSTN	Multilevel CR2 leave out Guo et al., 2025	56	1.24	0.65 to 1.83	<0.001	—	—
MSTN	Multilevel CR2 leave out Ghayomzadeh et al., 2022	56	1.26	0.67 to 1.85	<0.001	—	—
MSTN	Multilevel CR2 leave out Jeong et al., 2026	55	1.28	0.69 to 1.87	<0.001	—	—
MSTN	Multilevel CR2 leave out Ataeinosrat et al., 2022	54	1.17	0.60 to 1.73	<0.001	—	—
MSTN	Multilevel CR2 leave out Moqaddam et al., 2024	55	1.16	0.60 to 1.72	<0.001	—	—
MSTN	Multilevel CR2 leave out Saeidi et al., 2023	55	1.16	0.60 to 1.72	<0.001	—	—
MSTN	Multilevel CR2 leave out Pazokian et al., 2022	55	1.24	0.65 to 1.83	<0.001	—	—
MSTN	Multilevel CR2 leave out Seo et al., 2021	56	1.31	0.73 to 1.89	<0.001	—	—
MSTN	Multilevel CR2 leave out Razi et al., 2026	55	1.15	0.60 to 1.70	<0.001	—	—
MSTN	Multilevel CR2 leave out Turon-Skrzypinska et al., 2026	56	1.29	0.71 to 1.88	<0.001	—	—
MSTN	Multilevel CR2 leave out Mahboobi Semnan et al., 2026	55	1.25	0.66 to 1.84	<0.001	—	—
MSTN	Multilevel CR2 leave out Arazi et al., 2020	55	1.23	0.64 to 1.82	<0.001	—	—
MSTN	Multilevel CR2 leave out Arrieta et al., 2019	55	1.30	0.71 to 1.88	<0.001	—	—
MSTN	Multilevel CR2 leave out Shabkhiz et al., 2021	55	1.28	0.69 to 1.87	<0.001	—	—
MSTN	Multilevel CR2 leave out Boeselt et al., 2017	56	1.28	0.69 to 1.87	<0.001	—	—
MSTN	Multilevel CR2 leave out Farzanegi et al., 2021	54	1.22	0.63 to 1.81	<0.001	—	—

Outcome	Analysis	Effects	Hedges' g	95% CI	P value	τ^2	I^2 (%)
MSTN	Multilevel CR2 leave out Motahari Rad et al., 2023	55	1.27	0.68 to 1.86	<0.001	—	—
MSTN	Multilevel CR2 leave out Bagheri et al., 2019	54	1.28	0.69 to 1.86	<0.001	—	—
MSTN	Multilevel CR2 leave out Mafi et al., 2019	55	1.26	0.66 to 1.85	<0.001	—	—
MSTN	Multilevel CR2 leave out Willoughby et al., 2004	56	1.33	0.77 to 1.89	<0.001	—	—
MSTN	Multilevel CR2 leave out Bagheri et al., 2021	56	1.28	0.70 to 1.87	<0.001	—	—
MSTN	Multilevel CR2 leave out Saremi et al., 2010	56	1.19	0.62 to 1.76	<0.001	—	—
MSTN	Multilevel CR2 leave out Shabani et al., 2018	54	1.29	0.71 to 1.88	<0.001	—	—
MSTN	Multilevel CR2 leave out Riahy et al., 2024	55	1.26	0.67 to 1.85	<0.001	—	—
MSTN	Multilevel CR2 leave out Niazi et al., 2025	54	1.15	0.60 to 1.69	<0.001	—	—
MSTN	Multilevel CR2 leave out Nasiri et al., 2022	56	1.31	0.74 to 1.89	<0.001	—	—
MSTN	Multilevel CR2 leave out Rasi et al., 2023	56	1.18	0.61 to 1.75	<0.001	—	—
MSTN	Multilevel CR2 leave out Azamian Jazi et al., 2024	56	1.25	0.66 to 1.84	<0.001	—	—
MSTN	Multilevel CR2 leave out Barzegari Marvast et al., 2024	56	1.27	0.68 to 1.86	<0.001	—	—
MSTN	Multilevel CR2 leave out Ramezani et al., 2022	54	1.30	0.72 to 1.88	<0.001	—	—
FST	Multilevel CR2 leave out Ayaz et al., 2025	41	1.63	0.95 to 2.31	<0.001	—	—
FST	Multilevel CR2 leave out Jang et al., 2016	41	1.63	0.96 to 2.31	<0.001	—	—
FST	Multilevel CR2 leave out Rezaei et al., 2024	41	1.62	0.94 to 2.30	<0.001	—	—

Outcome	Analysis	Effects	Hedges' g	95% CI	P value	τ^2	I^2 (%)
FST	Multilevel CR2 leave out Jeong et al., 2026	40	1.59	0.91 to 2.27	<0.001	—	—
FST	Multilevel CR2 leave out Ataeinosrat et al., 2022	39	1.50	0.84 to 2.16	<0.001	—	—
FST	Multilevel CR2 leave out Moqaddam et al., 2024	40	1.45	0.83 to 2.08	<0.001	—	—
FST	Multilevel CR2 leave out Pazokian et al., 2022	40	1.62	0.93 to 2.31	<0.001	—	—
FST	Multilevel CR2 leave out Seo et al., 2021	41	1.62	0.94 to 2.30	<0.001	—	—
FST	Multilevel CR2 leave out Razi et al., 2026	40	1.45	0.83 to 2.08	<0.001	—	—
FST	Multilevel CR2 leave out Mahboobi Semnan et al., 2026	40	1.65	0.97 to 2.33	<0.001	—	—
FST	Multilevel CR2 leave out Arazi et al., 2020	40	1.52	0.85 to 2.20	<0.001	—	—
FST	Multilevel CR2 leave out Dantas et al., 2020	41	1.63	0.94 to 2.31	<0.001	—	—
FST	Multilevel CR2 leave out Farzanegi et al., 2021	39	1.56	0.87 to 2.25	<0.001	—	—
FST	Multilevel CR2 leave out Motahari Rad et al., 2023	40	1.65	0.96 to 2.33	<0.001	—	—
FST	Multilevel CR2 leave out Bagheri et al., 2019	39	1.53	0.85 to 2.21	<0.001	—	—
FST	Multilevel CR2 leave out Diel et al., 2010	40	1.66	0.98 to 2.33	<0.001	—	—
FST	Multilevel CR2 leave out Mafi et al., 2019	40	1.51	0.84 to 2.18	<0.001	—	—
FST	Multilevel CR2 leave out Bagheri et al., 2021	41	1.63	0.95 to 2.31	<0.001	—	—
FST	Multilevel CR2 leave out Mendez-Gutierrez A et al., 2023	40	1.65	0.97 to 2.33	<0.001	—	—
FST	Multilevel CR2 leave out Niazi et al., 2025	39	1.40	0.83 to 1.98	<0.001	—	—

Outcome	Analysis	Effects	Hedges’ g	95% CI	P value	τ^2	I^2 (%)
FST	Multilevel CR2 leave out Nasiri et al., 2022	41	1.64	0.96 to 2.31	<0.001	—	—
FST	Multilevel CR2 leave out Barzegari Marvast et al., 2024	41	1.59	0.90 to 2.28	<0.001	—	—
FST	Multilevel CR2 leave out Jaafari et al., 2024	40	1.59	0.90 to 2.28	<0.001	—	—

Correlation-sensitivity rows use study aggregation with Knapp–Hartung inference. Primary-model leave-one-family-out rows use multilevel REML and study-clustered CR2. Separate conventional working-independence diagnostics use REML/Knapp–Hartung. Paired τ^2 values denote between-study and within-study components; single τ^2 values belong to univariate models. These are exploratory specification checks, not independent confirmatory hypotheses.

Supplementary Table S5A. Exploratory exercise-modality subgroup estimates

Outcome	Model or comparison	Effects	Studies	Estimate	95% CI	P value	I^2 (%)
MSTN	Resistance-based	34	20	1.10	0.42 to 1.78	0.003	90.89
MSTN	Aerobic/concurrent	14	9	0.66	-0.12 to 1.43	0.085	84.45
MSTN	HIFT/CrossFit	6	3	4.23	2.63 to 5.82	0.008	0.00
MSTN	Other structured exercise	3	2	-0.31	-4.93 to 4.32	0.555	55.89
FST	Resistance-based	28	15	1.40	0.78 to 2.02	<0.001	84.32
FST	Aerobic/concurrent	9	6	0.57	0.12 to 1.01	0.023	38.66
FST	HIFT/CrossFit	4	2	4.30	-1.98 to 10.58	0.073	0.00
FST	Other structured exercise	1	1	—	— to —	—	—

Dependent effects were aggregated within study at $\rho = 0.5$, then fitted by REML with modified Knapp–Hartung inference and variance multiplier $\max(1, q)$. Degrees of freedom are $k - 1$, with k the number of contributing study clusters in that subgroup. One trial can contribute to more than one modality; subgroup counts must not be summed as independent studies. A one-study category is described but not pooled.

Supplementary Table S5B. Meta-regression and heterogeneity diagnostics for exercise modality

Analysis domain	Outcome	Model or comparison	Estimate	95% CI	P value	Effective df	Pseudo-R^2 (%)
Coefficient	MSTN	Modality model: resistance-based reference intercept	1.14	0.51 to 1.76	0.001	18.71	—
Coefficient	MSTN	Aerobic/concurrent versus resistance-based	-0.29	-1.08 to 0.50	0.379	4.72	—
Coefficient	MSTN	HIFT/CrossFit versus resistance-based	3.13	1.91 to 4.35	0.005	2.54	—
Coefficient	MSTN	Other structured exercise versus resistance-based	-1.53	-5.47 to 2.40	0.149	1.20	—
Coefficient	MSTN	Binary model: resistance-based reference intercept	1.23	0.62 to 1.83	<0.001	17.09	—

Analysis domain	Outcome	Model or comparison	Estimate	95% CI	P value	Effective df	Pseudo-R² (%)
Coefficient	MSTN	Other modalities versus resistance-based	0.06	-0.86 to 0.98	0.866	4.36	—
Coefficient	FST	Modality model: resistance-based reference intercept	1.54	0.75 to 2.34	<0.001	14.16	—
Coefficient	FST	Aerobic/concurrent versus resistance-based	-0.58	-1.90 to 0.74	0.290	4.12	—
Coefficient	FST	HIFT/CrossFit versus resistance-based	2.88	-0.18 to 5.95	0.054	1.23	—
Coefficient	FST	Other structured exercise versus resistance-based	-1.28	-2.08 to -0.49	0.004	14.16	—
Coefficient	FST	Binary model: resistance-based reference intercept	1.60	0.86 to 2.35	<0.001	13.66	—
Coefficient	FST	Other modalities versus resistance-based	-0.05	-1.20 to 1.10	0.906	3.98	—
Omnibus HTZ	MSTN	F (3, 1.95)	36.24	—	0.029	1.95	—
Omnibus HTZ	FST	F (3, 2.33)	5130.34	—	<0.001	2.33	—
Heterogeneity	MSTN	Modality model: residual Q	282.71	—	<0.001	—	40.17
Heterogeneity	MSTN	Binary model: residual Q	449.06	—	<0.001	—	0.00
Heterogeneity	FST	Modality model: residual Q	206.50	—	<0.001	—	38.07
Heterogeneity	FST	Binary model: residual Q	290.78	—	<0.001	—	0.00

Supplementary Table S5C. Exercise-modality restriction and leave-class-out sensitivity analyses

Outcome	Model or comparison	Effects	Studies	Estimate	95% CI	P value	I² (%)
MSTN	Leave out Resistance-based	23	14	1.29	0.23 to 2.35	0.021	95.86
MSTN	Leave out Aerobic/concurrent	43	25	1.35	0.63 to 2.06	<0.001	93.80
MSTN	Leave out HIFT/CrossFit	51	30	0.91	0.41 to 1.41	<0.001	91.19
MSTN	Leave out Other structured exercise	54	31	1.31	0.73 to 1.90	<0.001	93.40
MSTN	Resistance-based only	34	20	1.10	0.42 to 1.78	0.003	90.89
FST	Leave out Resistance-based	14	9	1.27	0.00 to 2.54	0.050	94.79
FST	Leave out Aerobic/concurrent	33	18	1.63	0.94 to 2.33	<0.001	89.23
FST	Leave out HIFT/CrossFit	38	21	1.14	0.68 to 1.60	<0.001	84.52
FST	Leave out Other structured exercise	41	22	1.45	0.87 to 2.02	<0.001	90.09

Outcome	Model or comparison	Effects	Studies	Estimate	95% CI	P value	I² (%)
FST	Resistance-based only	28	15	1.40	0.78 to 2.02	<0.001	84.32

All modality analyses were post hoc and exploratory. One-study subgroups were not pooled. Omnibus effective denominator degrees of freedom were 1.95 for MSTN and 2.33 for FST; nominal tests do not establish reliable modality differences or ranking.

Supplementary Table S6A. Coverage and feasibility of prespecified training-dose variables

Outcome	Variable	Effects	Studies	Unique values	Range	Decision
MSTN	frequency	56	32	2	2.00 to 3.00	Coverage reporting; no additional model
MSTN	weeks	53	30	6	6.00 to 36.00	Exploratory linear only; exclude calendar-month reports lacking exact weeks
MSTN	minutes	19	11	7	20.00 to 90.00	Coverage reporting; no additional model
MSTN	sessions	53	30	7	16.00 to 108.00	Exploratory linear only; exclude calendar-month reports lacking exact weeks
MSTN	total_minutes	16	8	6	320.00 to 4320.00	Coverage reporting; no additional model
FST	frequency	40	21	2	2.00 to 3.00	Coverage reporting; no additional model
FST	weeks	37	20	5	6.00 to 24.00	Exploratory linear only; exclude calendar-month reports lacking exact weeks
FST	minutes	11	6	4	20.00 to 70.00	Coverage reporting; no additional model
FST	sessions	36	19	6	16.00 to 72.00	Exploratory linear only; exclude calendar-month reports lacking exact weeks
FST	total_minutes	10	5	4	320.00 to 2880.00	Coverage reporting; no additional model

Note. A dose variable was modelled only when independent-study coverage and between-study variation were sufficient. Variables with invariant values or sparse reporting were not modelled.

Supplementary Table S6B. Exploratory dose meta-regression models

Outcome	Moderator	Term	Effects	Studies	Estimate	95% CI	P value	df	Status
MSTN	Duration per 4 weeks	Intercept	53	30	1.28	0.17 to 2.38	0.032	4.63	Exploratory
MSTN	Duration per 4 weeks	Slope	53	30	0.03	-0.33 to 0.40	0.717	1.93	Exploratory
MSTN	Sessions per 12 sessions	Intercept	53	30	1.28	0.23 to 2.32	0.026	4.92	Exploratory
MSTN	Sessions per 12 sessions	Slope	53	30	0.03	-0.30 to 0.37	0.707	2.03	Exploratory

Outcome	Moderator	Term	Effects	Studies	Estimate	95% CI	P value	df	Status
FST	Duration per 4 weeks	Intercept	37	20	1.99	-0.78 to 4.77	0.121	4.46	Exploratory
FST	Duration per 4 weeks	Slope	37	20	-0.09	-1.54 to 1.35	0.833	2.50	Exploratory
FST	Sessions per 12 sessions	Intercept	36	19	2.04	-0.77 to 4.85	0.119	4.66	Exploratory
FST	Sessions per 12 sessions	Slope	36	19	-0.09	-1.50 to 1.33	0.851	2.66	Exploratory

Note. Slope estimates are exploratory and should not be interpreted as an optimal dose. CI, confidence interval; df, effective degrees of freedom.

Supplementary Table S7. GRADE evidence profile for the two primary outcomes

Outcome	Evidence base	Summary effect	Main limitations	Certainty	Interpretation
MSTN	33 studies; 57 effects	*1.25 (0.68 to 1.82)*	Serious risk of bias and inconsistency; domain judgments reviewed by CQ/CY	Low	Mean concentration difference, not clinical benefit
FST	23 studies; 42 effects	*1.58 (0.92 to 2.23)*	Serious risk of bias and inconsistency; domain judgments reviewed by CQ/CY	Low	Mean concentration difference, not clinical benefit

GRADE addresses the mean circulating concentration difference. CQ and CY reviewed one-level downgrades for risk of bias and inconsistency, resulting in Low certainty for both outcomes. No additional imprecision downgrade was applied to the pooled-average estimand; this does not establish a precise magnitude or consistent effect in a new setting. Graph-exclusion intervals exclude zero, but availability, extraction and between-study uncertainty remain. Circulating concentrations do not establish clinical benefit or intracellular mediation.

Supplementary Table S7 (continued). Domain-level certainty judgments for the two primary outcomes

Outcome	GRADE domain	Judgment	Levels down	Reason
MSTN	Risk of bias	Serious	1	MSTN has 32 randomized and one nonrandomized primary study families. Source-specific concerns justify one downgrade; CQ and CY reviewed the contributing-study assessments.
MSTN	Inconsistency	Serious	1	Working-independence reference-model I^2 was 92.56%, with a prediction interval of −1.74 to 4.57. Excluding $\|g\| > 3$ reduced the primary estimate from 1.25 to 0.56. Substantial variation and sensitivity of magnitude support one downgrade; the prediction interval concerns between-setting variation, not uncertainty in the pooled mean.
MSTN	Indirectness	Not serious	0	Circulating MSTN was directly measured in adult exercise interventions, matching the biochemical outcome assessed here. This judgment applies to concentration differences, not clinical benefit, diagnostic performance, or muscle intracellular signaling; none of those was established by this synthesis.
MSTN	Imprecision	Not serious for direction	0	The primary CR2 interval excludes zero for the pooled mean direction, as does the graph-exclusion sensitivity interval. Effect magnitude remains uncertain and prediction intervals cross zero; this judgment does not establish a precise magnitude or consistent effects across settings.
MSTN	Publication bias	Uncertain; no additional downgrade	0	Formal asymmetry tests were not performed; their absence does not establish absence of bias. Seventeen of 220 sought reports remained unavailable after renewed retrieval, and the original search omissions remain unresolved. Result-dependent missingness was not established, so no additional downgrade was assigned; publication and availability bias remain explicit limitations.
FST	Risk of bias	Serious	1	FST has 23 randomized primary study families; unrelated ROBINS-I judgments are not imported. Source-specific concerns justify one downgrade; CQ and CY reviewed the contributing-study assessments.
FST	Inconsistency	Serious	1	Working-independence reference-model I^2 was 92.89%, with a prediction interval of −1.46 to 5.35. Excluding $\|g\| > 3$ reduced the primary estimate from 1.58 to 1.00. Substantial variation and sensitivity of magnitude support one downgrade; the prediction interval concerns between-setting variation, not uncertainty in the pooled mean.

Outcome	GRADE domain	Judgment	Levels down	Reason
FST	Indirectness	Not serious	0	The question concerns measured circulating FST concentrations in adults. FST is neither muscle-specific nor a validated clinical surrogate, but this does not make the directly measured biochemical outcome indirect for that question. Clinical benefit or a confirmed muscle-signaling response was not inferred.
FST	Imprecision	Not serious for direction	0	The primary CR2 interval excludes zero for the pooled mean direction, as does the graph-exclusion sensitivity interval. Effect magnitude remains uncertain and prediction intervals cross zero; this judgment does not establish a precise magnitude or consistent effects across settings.
FST	Publication bias	Uncertain; no additional downgrade	0	Formal asymmetry tests were not performed; their absence does not establish absence of bias. Seventeen of 220 sought reports remained unavailable after renewed retrieval, and the original search omissions remain unresolved. Result-dependent missingness was not established, so no additional downgrade was assigned; publication and availability bias remain explicit limitations.

Supplementary Table S8A. RoB 2 domain-level and overall judgements

Study	D1	D2	D3	D4	D5	Overall
Ayaz et al., 2025	Low	Some concerns	Some concerns	Low	Low	Some concerns
Zhuang et al., 2025	Some concerns	Some concerns	Some concerns	Low	Some concerns	Some concerns
Jeong et al., 2026	Low	Some concerns	Low	Low	Low	Some concerns
Zhang et al., 2024	Some concerns	Low	Some concerns	Low	Low	Some concerns
Ataeinosrat et al., 2022	Some concerns	Some concerns	Low	Low	Some concerns	Some concerns
Moqaddam et al., 2024	Low	Some concerns	Low	Low	Some concerns	Some concerns
Hofmann et al., 2016	Some concerns	Low	Some concerns	Low	Some concerns	Some concerns
Seo et al., 2021	Low	Low	Low	Low	Some concerns	Some concerns
Saeidi et al., 2023	Some concerns	Low	Some concerns	Low	Some concerns	Some concerns
Arazi et al., 2020	Some concerns	Some concerns	Some concerns	Low	Some concerns	Some concerns
Mahboobi Semnan et al., 2026	Some concerns	Some concerns	Low	Low	Low	Some concerns
Rezaei et al., 2024	Some concerns	Some concerns	Low	Low	Some concerns	Some concerns
Antunes-Correa et al., 2020	Some concerns	Some concerns	High	Low	Some concerns	High
Dantas et al., 2020	Low	Some concerns	Low	Low	Low	Some concerns
Shahrokhian et al., 2022	Some concerns	Some concerns	Some concerns	Low	Some concerns	Some concerns
Binns et al., 2017	Low	Some concerns	Low	Low	Low	Some concerns
Kortas et al., 2020	Some concerns	Some concerns	Some concerns	Low	Some concerns	Some concerns
Zhou et al., 2021	Low	Some concerns	Low	Low	Low	Some concerns
Guo et al., 2025	Some concerns	Low	Low	Low	Some concerns	Some concerns
Brooks et al., 2010	Some concerns	Some concerns	Low	Low	Some concerns	Some concerns
Dawson et al., 2021	Some concerns	Some concerns	Low	Low	Low	Some concerns
Ghayomzadeh et al., 2022	Low	Some concerns	Low	Low	Some concerns	Some concerns
Gould et al., 2025	Some concerns	Some concerns	Some concerns	Low	Low	Some concerns
Zuo et al., 2023	Low	Some concerns	Some concerns	Low	Some concerns	Some concerns
Watson et al., 2017	Some concerns	Some concerns	Low	Low	Some concerns	Some concerns
Radziminska et al., 2018	Some concerns	Low	Some concerns	Low	Some concerns	Some concerns
Razi et al., 2026	Low	Some concerns	Some concerns	Low	Some concerns	Some concerns

Study	D1	D2	D3	D4	D5	Overall
Turon-Skrzypinska et al., 2026	Some concerns	Low	Low	Low	Some concerns	Some concerns
Brown et al., 2024	Low	Some concerns	Low	Low	Low	Some concerns
Pazokian et al., 2022	Some concerns	Low	Low	Low	Some concerns	Some concerns
Jang et al., 2016	Low	Some concerns	Low	Low	Some concerns	Some concerns
Arrieta et al., 2019	Some concerns	Some concerns	Some concerns	Low	Some concerns	Some concerns
Motahari Rad et al., 2023	Some concerns	Some concerns	Some concerns	Low	Some concerns	Some concerns
Bagheri et al., 2019	Some concerns	Some concerns	Low	Low	Some concerns	Some concerns
Bagheri et al., 2020	Some concerns	Some concerns	Some concerns	Low	Some concerns	Some concerns
Diel et al., 2010	Some concerns	Some concerns	Some concerns	Low	Some concerns	Some concerns
Mafi et al., 2019	Some concerns	Some concerns	Some concerns	Low	Some concerns	Some concerns
Ghobadi et al., 2024	Some concerns	Some concerns	Low	Low	Some concerns	Some concerns
Willoughby et al., 2004	Some concerns	Some concerns	Low	Low	Some concerns	Some concerns
Koczulla et al., 2020	Some concerns	Some concerns	Low	Low	Some concerns	Some concerns
Bagheri et al., 2021	Some concerns	Some concerns	Low	Low	Some concerns	Some concerns
Saremi et al., 2010	Some concerns	Some concerns	Some concerns	Low	Some concerns	Some concerns
Lenk et al., 2012	Some concerns	Some concerns	Some concerns	Low	Some concerns	Some concerns
Shabkhiz et al., 2021	Some concerns	Some concerns	Low	Low	Some concerns	Some concerns
Schiffer et al., 2011	Some concerns	Some concerns	Some concerns	Low	Some concerns	Some concerns
Heisterberg et al., 2018	Some concerns	Some concerns	Some concerns	Low	Some concerns	Some concerns
Nilsen TS et al., 2016	Some concerns	Some concerns	Some concerns	Low	Some concerns	Some concerns
Hulmi et al., 2009 / Mero et al., 2013	Some concerns	Some concerns	Some concerns	Low	Some concerns	Some concerns
Lewis et al., 2007	Some concerns	Some concerns	Some concerns	Low	Some concerns	Some concerns
Santos et al., 2015	Some concerns	Some concerns	Low	Low	Some concerns	Some concerns
Lundberg et al., 2013	Some concerns	Some concerns	Low	Low	Some concerns	Some concerns
Laurentino et al., 2012	Some concerns	Some concerns	Low	Low	Some concerns	Some concerns
Walker et al., 2004	Some concerns	Some concerns	Low	Low	Some concerns	Some concerns
Mendez-Gutierrez A et al., 2023	Some concerns	Some concerns	High	Low	Some concerns	High
Bozyilan et al., 2026	Some concerns	Some concerns	Low	Low	Some concerns	Some concerns
Strojek et al., 2021	Some concerns	Some concerns	Some concerns	Low	Some concerns	Some concerns
Farzanegi et al., 2021	Some concerns	Some concerns	Low	Low	Some concerns	Some concerns
Barati et al., 2025	Some concerns	Some concerns	Low	Low	Some concerns	Some concerns
Shabani et al., 2018	Some concerns	Some concerns	Low	Low	Some concerns	Some concerns
Karimifard et al., 2023	Some concerns	Some concerns	Some concerns	Low	Some concerns	Some concerns
Bijeh et al., 2022	Some concerns	Some concerns	Some concerns	Low	Some concerns	Some concerns
Riahy et al., 2024	Some concerns	Some concerns	Some concerns	Low	Some concerns	Some concerns
Niazi et al., 2025	Some concerns	Some concerns	Some concerns	Low	Some concerns	Some concerns
Nasiri et al., 2022	Some concerns	Some concerns	Some concerns	Low	Some concerns	Some concerns

Study	D1	D2	D3	D4	D5	Overall
Sönmez et al., 2026	Some concerns	Some concerns	High	Low	Some concerns	High
de Souza et al., 2014	Some concerns	Some concerns	Some concerns	Low	Some concerns	Some concerns
Rasi et al., 2023	Some concerns	Some concerns	Low	Low	Some concerns	Some concerns
Hooshmandi et al., 2022	Some concerns	Some concerns	Low	Some concerns	Some concerns	Some concerns
Azamian Jazi et al., 2024	Some concerns	Some concerns	Low	Low	Some concerns	Some concerns
Jaafari et al., 2024	Some concerns	Some concerns	Low	Low	Some concerns	Some concerns
Barzegari Marvast et al., 2024	Some concerns	Some concerns	Some concerns	Low	Some concerns	Some concerns
Ramezani et al., 2022	Some concerns	Some concerns	Low	Low	Some concerns	Some concerns
Watson et al., 2022	Some concerns	Some concerns	Some concerns	Low	Some concerns	Some concerns

D1, randomization; D2, deviations from intended interventions; D3, missing outcome data; D4, outcome measurement; D5, selection of the reported result. CQ and CY reviewed all judgments. Inability to blind exercise participants alone is not automatically High risk. Measurement judgments are outcome-specific; Hooshmandi's laboratory MSTN/IGF-I and ultrasound-dependent MSTN/CSA ratios differ in D4, as retained in File S2. Izaddoust et al. (2017) is a companion/subset report of Shabani et al. (2018); its family-level RoB 2 assessment is represented by the Shabani et al. (2018) row, with an overall judgment of Some concerns. It is not an additional independent assessment.

Reassessment notes. Hofmann, Saeidi, Antunes-Correa and Radziminska explicitly report random allocation and were moved from ROBINS-I to RoB 2. Their D1 judgments reflect incomplete concealment or sequence reporting, not demonstrated nonrandom allocation. Antunes-Correa had 33 of 44 randomized patients analyzed after exclusions including death, heart-failure decompensation, cardiovascular events and cancer; D3 and overall risk were judged High. A minimum attendance requirement also raised D2 concerns. Hofmann had blood samples for 91 women at baseline and 70 at six months; Saeidi had 60 of 68 complete follow-up; Radziminska had 74 of 84 complete follow-up. The latter three received Some concerns for D3 because missing-data explanations or robustness analyses did not establish absence of material bias. Lack of a prospectively fixed biomarker analysis plan supported Some concerns in D5, without asserting proven selective reporting.

Shahrokhian explicitly reports random division into groups, although the abstract/results describe training and control whereas Methods describes three arms. RoB 2 was retained; unclear allocation safeguards, post-allocation exclusion rules, missing participant-flow denominators and the absence of a prospectively fixed analysis plan support Some concerns in D1, D2, D3 and D5. Pazokian reports independent block randomization and all 10 participants per arm analyzed: D2–D4 remain Low. Concealment safeguards and a prospectively fixed analysis plan were not established, so D1 and D5 are Some concerns, yielding overall Some concerns. Multiple Some concerns domains were not automatically converted to High when the identified reporting gaps did not establish substantially reduced confidence. No algorithm override was used to retain an overall judgment above five Low domains.

Supplementary Table S8B. ROBINS-I domain-level and overall judgements

Study	D1	D2	D3	D4	D5	D6	D7	Overall
Planella-Farrugia et al., 2019	Serious	Moderate	Low	Moderate	Moderate	Low	No information	Serious
Benavente et al., 2024	Serious	No information	Low	Moderate	Moderate	Low	Moderate	Serious
Micielska et al., 2019	Serious	Moderate	Low	Moderate	No information	Low	No information	Serious
Boeselt et al., 2017	Serious	Moderate	Low	Moderate	No information	Low	No information	Serious
Irimia et al., 2017	Serious	Moderate	Low	Moderate	No information	Low	No information	Serious

Note. D1, confounding; D2, selection of participants; D3, classification of interventions; D4, deviations from intended interventions; D5, missing data; D6, measurement of outcomes; D7, selection of the reported result.

Planella-Farrugia initially randomized 15 controls, 14 resistance-exercise participants and nine resistance-plus-nutrition participants using EPIDAT, then recruited five additional controls. Published combined-control results do not separate the randomized-only comparison. ROBINS-I and its Serious confounding judgment were therefore retained for that comparison, and the design descriptor in Table 1 was corrected. The other ROBINS-I domain judgments are unchanged in this targeted allocation correction.

Supplementary Table S9. Secondary muscle-expression and FST/MSTN ratio outcomes

Study	Outcome and level	Comparison and sampling	Reported finding	Synthesis handling	Source location
Antunes-Correa et al., 2020	Muscle *FST* mRNA	AET, IMT and control; chronic intervention	*FST* mRNA increased after AET and decreased in control; IMT showed no clear change.	Narrative; baseline-normalized expression and small biopsy samples; not circulating FST.	Figure 3F; PDF pp7–8
Dantas et al., 2020	Muscle *MSTN* transcript; FST protein	RYGB + exercise versus RYGB; muscle biopsy	The report describes stronger MSTN downregulation and restoration/increase of FST with exercise.	Narrative tissue evidence; circulating FST in Table 2 is a separate primary input.	Figures 3–4; PDF pp11–13
Brooks et al., 2010	Muscle *MSTN* mRNA	EAA alone versus EAA + RT during bed rest/recovery	*MSTN* transcripts increased during bed rest in EAA alone; no corresponding within-group time effect in RT arms.	Narrative; bed-rest context and recovery phase kept distinct; no between-group claim inferred from differing within-group significance.	Results; Figure 2A
Dawson et al., 2021	Muscle *MSTN* mRNA	Resistance training versus stretching; 12 weeks	No clear post-intervention between-group difference in MSTN fold change (reported $p > 0.05$).	Narrative; median/IQR fold-change data, not a common mean/SD tissue estimate.	Figure 2; PDF p6
Gould et al.	Muscle *MSTN* mRNA	Aerobic versus concurrent training; acute bouts before/after training	Time-related expression changes were reported, without a group or group × time effect for MSTN.	Narrative acute-response evidence; not a resting chronic concentration or protein effect.	Figure 4C–D; PDF pp6,9
Watson et al., 2017	Muscle *MSTN* mRNA	Acute resistance bouts before/after 8-week training	*MSTN* mRNA was suppressed after acute exercise in both training states; control expression showed no clear change over time.	Narrative acute-response evidence; not proof of a chronic resting reduction.	Results; PDF p5
Jeong et al., 2026	Reported circulating FST/MSTN ratio change	Resistance exercise with/without EAA; factorial controls	RE + EAA showed a greater ratio increase than control and EAA-only groups.	Narrative reported percentage change; supplementation-matched contrasts retained conceptually, no pooled ratio reconstructed.	Section 3.4; Figure 4; PDF pp9–11
Mahboobi Semnan et al., 2026	Reported circulating FST/MSTN ratio	WB-EMS versus RT versus control	Reported ratio improvements were 48.9% with WB-EMS and 20.8% with RT; the report notes baseline variability.	Narrative reported ratio, distinct from separately extracted circulating MSTN/FST effects.	Results; Figure 5; PDF pp7,9
Pazokian et al., 2022	Reported circulating FST/MSTN ratio	Functional training with/without BFR versus control	Reported ratio changes were +47.1% (FT), +73.5% (FTBFR) and −4.5% (control).	Narrative reported ratio; no superiority inferred from different within-group changes.	Results; Figure 5; PDF p7
Bagheri, R. (2019)	MSTN/FST; serum; ELISA	Upper-, lower-, or combined-body RT; 3/week versus Nonexercise control; 8 weeks	Source-reported FST:MSTN ratios were assessed alongside the two circulating markers. Ratios were not reconstructed from marginal means or pooled as concentrations.	Three contrasts share one control; Figure 2 states SD	Bagheri et al., 2019; full report, PDF pp. 1–24
Bagheri, R (2020)	MSTN/FST; serum; immunoassays	Endurance then resistance or reverse order; 3/week versus Nonexercise control; 8 weeks	The report describes circulating MSTN/FST responses to different concurrent-training orders. The supplied pre-proof omits the target graph itself; no numerical effect or control dispersion was reconstructed from its caption.	Supplied pre-proof has Figure 2 caption but no actual target graph or extractable control dispersion; narrative only	Bagheri et al., 2020; full report, PDF pp. 1–22
Mafi, F (2019)	MSTN/FST; plasma in Methods; figure captions say serum	RT with or without epicatechin; 3/week versus Placebo or epicatechin-only matched strata; 8 weeks	The source reports FST:MSTN ratio responses as well as matched-supplement training contrasts. Ratios remain separate; their variance was not derived without a reported within-sample covariance.	Change-score SD; RT vs placebo and RT+EP vs EP only; specimen wording conflict retained	Mafi et al., 2019; full report, PDF pp. 1–27

Study	Outcome and level	Comparison and sampling	Reported finding	Synthesis handling	Source location
Ghobadi, H. (2024)	Circulating MSTN/FST	Autoregulated versus linear periodized resistance exercise; 3/week versus Active RT comparator; 8 weeks	Neither circulating FST nor MSTN differed significantly over time or between autoregulated and linear resistance-training groups. This is a direct active-training comparison, not evidence of no exercise effect versus nonexercise.	Narrative direct-training comparison; no nonexercise arm	Ghobadi et al., 2024; full report, PDF pp. 1–10
Willoughby, D.S. (2004)	MSTN; serum and muscle protein/mRNA; noncommercial immunoassay	Heavy resistance training; 3/week versus Nontraining control; 12 weeks	Muscle MSTN mRNA/protein and serum immunoreactivity were measured separately. Only compatible serum data enter the concentration model; tissue expression does not establish circulating bioactivity.	Serum and muscle levels separated; noncommercial assay sensitivity retained	Willoughby et al., 2004; full report, PDF pp. 1–9
Koczulla, A.R. (2020)	MSTN; circulating	Whole-body vibration at therapeutic frequency versus Low-frequency sham vibration with the same exercises; 12 weeks	The therapeutic-vibration group showed a within-group MSTN reduction of 465 pg/mL (95% CI for change −713 to −166); this within-group interval does not establish superiority over the low-frequency sham exercise comparator.	Direct vibration-dose contrast; not pooled as inactive control	Koczulla et al., 2020; full report, PDF pp. 1–9
Lenk, K. (2012)	Muscle MSTN mRNA/protein; circulating MSTN mentioned	Supervised exercise training versus Sedentary control; 12 weeks	After training, muscle MSTN mRNA and protein decreased by approximately 36% and 23% relative to baseline. Serum MSTN showed no reported significant between-group alteration, but numerical serum data were explicitly not shown. Tissue percentages were not converted into a circulating SMD.	Serum numerical data explicitly not shown; secondary muscle outcomes only	Lenk et al., 2012; full report, PDF pp. 1–8
Schiffer, T (2011)	MSTN mRNA; vastus lateralis	Strength or moderate endurance training versus Nontraining control; 12 weeks	The report did not identify significant chronic MSTN mRNA changes or clear strength-versus-endurance-versus-control differences. Tissue expression was not pooled with blood concentrations.	No clear chronic group differences; not pooled with blood concentrations	Schiffer et al., 2011; full report, PDF pp. 1–5
Heisterberg, M.F. (2018)	MSTN expression; skeletal muscle; RT-PCR	Heavy RT plus losartan or placebo versus Losartan without RT; 16 weeks	Muscle expression was evaluated in a losartan-by-training design. The matched losartan-plus-training versus losartan-without-training contrast is retained; the report does not establish a circulating MSTN effect, and no unmatched drug contrast enters the primary analysis.	Use losartan+RT versus losartan-only to isolate exercise; placebo+RT is not a matched drug comparison	Heisterberg et al., 2018; full report, PDF pp. 1–60
Micielska, K. (2019)	MSTN; circulating	High-intensity circuit training; 15 sessions versus No chronic training; two assessment bouts; 5 weeks	The report includes chronic high-intensity circuit training and separate assessment-bout responses. Target-graph dispersion could not be established as SD or SE; no primary SMD was calculated from an assumed error definition.	Not synthesized quantitatively because the target graph did not clearly identify the error-bar definition.	Micielska et al., 2019; full report, PDF pp. 1–25

Study	Outcome and level	Comparison and sampling	Reported finding	Synthesis handling	Source location
Nilsen TS (2016)	MSTN protein; muscle cellular measurements	Strength training versus Usual care without strength training; 16 weeks	Muscle MSTN protein did not change after the 16-week strength-training intervention in men receiving androgen-deprivation therapy. This cellular outcome is not a circulating concentration.	Secondary outcome; not circulating concentration	Nilsen TS (2016) et al., 2016; full report, PDF pp. 1–10
Hulmi, J.J. (2009)	MSTN mRNA; vastus lateralis	RT twice weekly with protein or placebo versus No resistance training; 21 weeks	Chronic resting and acute post-bout biopsies were reported. The approximately 31% placebo-group MSTN mRNA decrease occurred one hour after a bout and is not a chronic resting effect. Only this representative report was retained; overlapping companion reports were excluded.	Chronic resting and acute biopsy contrasts separated; overlapping companion reports excluded	Hulmi et al., 2009; full report, PDF pp. 1–12
Lewis, M.I. (2007)	MSTN mRNA; skeletal muscle	Resistance training with or without testosterone versus Matched no-training hormone stratum; 10 weeks	Muscle MSTN mRNA remained unchanged in the training/testosterone factorial study. Only hormone-matched exercise contrasts are conceptually eligible; tissue expression is not a blood concentration.	Hormone cointervention matched; do not mix unmatched testosterone effects	Lewis et al., 2007; full report, PDF pp. 1–12
Santos, A.R. (2015)	MSTN and canonical FST gene expression; muscle	Strength versus power resistance-training regimens; 3/week versus Power versus strength is randomized; separate nontraining control; 8 weeks	Muscle canonical FST mRNA increased approximately 4.2-fold with strength and 3.7-fold with power training; MSTN mRNA did not change. FSTL3 was separately measured and is not canonical FST. Training groups were randomized; the nontraining control was separately recruited.	Narrative tissue outcome; RoB 2 assessment targets randomized power-versus-strength comparison, not the separately recruited control contrast	Santos et al., 2015; full report, PDF pp. 1–8
Lundberg, T.R. (2013)	MSTN gene expression; vastus lateralis	Unilateral aerobic plus resistance exercise versus Contralateral resistance-only training; 5 weeks	Concurrent aerobic-plus-resistance training suppressed muscle MSTN expression in a within-person leg design. The paired active-training contrast was not treated as an independent exercise-versus-inactive comparison.	Paired active comparison; no independent-groups SMD	Lundberg et al., 2013; full report, PDF pp. 1–10
Laurentino, G.C. (2012)	MSTN/FST expression; skeletal muscle	Low-load RT with blood-flow restriction or high-load RT versus Low-load RT without restriction; 8 weeks	Muscle MSTN mRNA decreased approximately 45% with low-load blood-flow restriction and 41% with high-load training. Canonical FST and separately measured FSTL3 mRNA increased across training groups. These within-group tissue responses were not pooled as serum/plasma effects.	Active training contrasts and tissue expression only	Laurentino et al., 2012; full report, PDF pp. 1–7
Walker, K.S. (2004)	MSTN; plasma Western blot	Whole-body resistance training versus Elbow-flexor resistance training; 10 weeks	Plasma MSTN Western-blot immunoreactivity declined by approximately 20% in both whole-body and elbow-flexor training conditions. Relative immunoreactivity and an active comparator preclude pooling as an inactive-control mass-concentration effect.	Active comparator and relative immunoreactivity; narrative only	Walker et al., 2004; full report, PDF pp. 1–7

Study	Outcome and level	Comparison and sampling	Reported finding	Synthesis handling	Source location
Irimia, J.M. (2017)	MSTN gene expression; vastus lateralis	Flywheel resistance exercise during bed rest versus Bed rest without exercise; 84 days	Bed rest increased muscle MSTN expression, whereas the flywheel-exercise countermeasure attenuated the bed-rest response. The bed-rest tissue comparison is retained separately from ordinary ambulatory circulating outcomes.	Secondary tissue outcome; no conversion to circulating MSTN	Irimia et al., 2017; full report, PDF pp. 1–8
Bozyilan, E. (2026)	Circulating MSTN	Added HIIT; usual kickboxing retained in both groups versus Usual kickboxing training; 8 weeks	An eight-week HIIT add-on was compared with usual kickboxing, not with an inactive control. The active-training comparison is retained separately from the primary pooled estimand.	Active training comparator; narrative only	Bozyilan et al., 2026; full report, PDF pp. 1–15
Strojek, K. (2021)	MSTN; circulating	Supervised pelvic-floor muscle training twice weekly versus No intervention; 12 weeks	Pelvic-floor training was associated with a within-group decrease in median serum MSTN, with baseline imbalance between groups. Medians and IQRs were not converted to means and SDs under an unplanned assumption.	Median/IQR presentation and baseline imbalance; no unplanned conversion to means/SD	Strojek et al., 2021; full report, PDF pp. 1–10
Barati, N. (2025)	FST; circulating	Linear versus weekly undulating periodized RT versus Active RT comparator; 9 weeks	Neither linear nor weekly undulating training significantly changed circulating FST, and the between-group FST comparison was not significant ($p = 0.381$). Both arms trained; this does not estimate exercise versus inactivity.	Narrative active-training comparison	Barati et al., 2025; full report, PDF pp. 1–7
Karimifard, M. (2023)	MSTN/FST; circulating	Rest-pause or traditional resistance training; 3/week versus Nontraining control; 12 weeks	The source describes increased FST, decreased MSTN and higher FST:MSTN ratio after rest-pause or traditional training. The graph uncertainty definition and final control denominator remain insufficiently clear for a primary SMD; no error type or ratio covariance was invented.	Not synthesized quantitatively because the graph uncertainty type and final control denominator were insufficiently reported.	Karimifard et al., 2023; full report, PDF pp. 1–14
Bijeh et al., 2022	Serum MSTN/FST and source-reported MSTN:FST ratio	RT with/without soy milk; 3 sessions/week; 12 weeks	Undefined target-graph error bars; narrative only	Descriptive; ratios not reconstructed and active contrasts not pooled with nonexercise	Bijeh et al., 2022; full report, PDF pp. 1–14
Watson et al., 2022	Circulating and muscle MSTN	Aerobic versus combined aerobic/RT; 12 weeks	Author-manuscript conversion; independent of Gould after author re-review; narrative only	Descriptive; ratios not reconstructed and active contrasts not pooled with nonexercise	Watson et al., 2022; full report, PDF pp. 1–33
de Souza et al., 2014	Muscle MSTN mRNA	Strength, interval or concurrent training; 8 weeks	No clear training-related MSTN mRNA change; FSTL3 is not canonical FST. Tissue expression is not pooled with circulating concentrations.	Muscle-level narrative; distinct baseline/training history from Santos	de Souza et al., 2014; full report, PDF pp. 1–25
Hooshmandi et al., 2022	Source MSTN/IGF-I and MSTN/CSA ratios	High-intensity interval resistance training; 8 weeks	Source reports ratios rather than compatible absolute MSTN concentrations. No absolute marker mean/SD was reconstructed from ratios.	Ratios only; no reconstruction of absolute MSTN	Hooshmandi et al., 2022; full report, PDF pp. 1–8

Study	Outcome and level	Comparison and sampling	Reported finding	Synthesis handling	Source location
Sönmez et al., 2026	Serum MSTN; active-training comparison	HIIT versus moderate continuous training; 6 weeks	Six-week HIIT versus moderate continuous training; no nonexercise comparator, so no primary exercise-versus-nonexercise effect was added.	Active-comparator narrative only; no exercise versus nonexercise primary effect	Sönmez et al., 2026; full report, PDF pp. 1–12

Note. Evidence coverage: 36 descriptive report entries, including 19 with muscle-level measurements and six reporting circulating FST:MSTN ratios; these categories overlap. Bijeh reports the opposite MSTN:FST orientation, and Hooshmandi reports MSTN/IGF-I and MSTN/CSA, which are kept separate. Results are reported at their original measurement level and are not a vote-counting synthesis. Reported within-group changes do not establish a between-group effect. Acute-bout responses, resting chronic outcomes, transcript abundance, tissue protein and circulating ratios were not pooled. Ratio effects were not reconstructed from the ratio of group means or marginal biomarker SDs; participant-level covariance was not assumed. Ryan's uncontrolled report was removed after eligibility reassessment and is listed in Table S1, not included in this secondary synthesis. AET, aerobic exercise training; IMT, inspiratory muscle training; EAA, essential amino acids; RYGB, Roux-en-Y gastric bypass; RT, resistance training; WB-EMS, whole-body electromyostimulation; BFR, blood-flow restriction.

Evidence update. The original 138-record retrieval queue supplied 121 original records for assessment; 17 remained unavailable. The review contains 79 reports from 78 study families, and primary analyses contain 99 effects from 39 study families. CQ and CY reviewed eligibility decisions, numerical inputs and domain-level risk judgments in separate stages. The original 90 numerical effects were unchanged; nine directly reported effects were added. Expanded PubMed records were not screened or incorporated.

Supplementary Table S10. Operational exercise-modality assignments

Post hoc classification. HIFT/CrossFit requires an explicitly described mixed workout; interval resistance training remains resistance-based. Counts are primary circulating MSTN/FST effects. No primary moderator assignment is imposed on studies without a compatible effect.

Study	Reported training	Operational class	Primary effects MSTN/FST	Basis
Ayaz et al., 2025	Combined aerobic and resistance training	Aerobic/concurrent	0/1	Combined aerobic and resistance programme.
Zhuang et al., 2025	Whole-body vibration or resistance training	Resistance-based (RT); Other targeted modality (WBV)	0/0	Arm-specific classification; WBV was not assumed equivalent to conventional resistance training.
Jeong et al., 2026	Circuit resistance training	Resistance-based	2/2	Progressive loading, resistance or strength practice predominates; not a mixed CrossFit WOD.
Zhang et al., 2024	Low-load BFR resistance training or conventional high-intensity resistance training	Resistance-based	0/0	Progressive loading, resistance or strength practice predominates; not a mixed CrossFit WOD.
Ataeinosrat et al., 2022	Low-, moderate-, or high-intensity interval resistance training	Resistance-based	3/3	All three arms use 10 resistance exercises and sets at 40%, 60% or 80% 1RM with active resistance rest. Interval structure/intensity alone does not define HIFT.
Moqaddam et al., 2024	CrossFit/high-intensity functional training	HIFT/CrossFit	2/2	Explicit CrossFit/HIFT programme with mixed strength/endurance WODs; classification takes precedence over the general concurrent category.
Hofmann et al., 2016	Elastic-band resistance training	Resistance-based	0/0	Progressive loading, resistance or strength practice predominates; not a mixed CrossFit WOD.
Seo et al., 2021	Bodyweight plus elastic-band resistance training	Resistance-based	1/1	Progressive loading, resistance or strength practice predominates; not a mixed CrossFit WOD.

Study	Reported training	Operational class	Primary effects MSTN/FST	Basis
Saeidi et al., 2023	HIFT/CrossFit	HIFT/CrossFit	2/0	Explicit CrossFit/HIFT programme with mixed strength/endurance WODs; classification takes precedence over the general concurrent category.
Arazi et al., 2020	Variable- or constant-resistance training	Resistance-based	2/2	Progressive loading, resistance or strength practice predominates; not a mixed CrossFit WOD.
Mahboobi Semnan et al., 2026	Whole-body EMS or resistance training	Resistance-based	2/2	Conventional RT and voluntary bodyweight WB-EMS arms; bodyweight squats/lunges with EMS are classified as resistance-based, not passive stimulation.
Rezaei et al., 2024	TRX suspension training	Resistance-based	1/1	Progressive loading, resistance or strength practice predominates; not a mixed CrossFit WOD.
Antunes-Correa et al., 2020	Aerobic or inspiratory muscle training	Aerobic/concurrent (AET); Other targeted exercise (IMT)	0/0	Whole-body aerobic and isolated inspiratory training arms are distinguished; muscle FST is not a circulating moderator input.
Dantas et al., 2020	Combined aerobic and resistance training	Aerobic/concurrent	0/1	Combined aerobic and resistance programme.
Shahrokhian et al., 2022	Nonlinear or linear resistance training	Resistance-based	1/0	Progressive loading, resistance or strength practice predominates; not a mixed CrossFit WOD.
Binns et al., 2017	High-velocity resistance training	Resistance-based	0/0	Progressive loading, resistance or strength practice predominates; not a mixed CrossFit WOD.
Planella-Farrugia et al., 2019	Low-intensity resistance exercise	Resistance-based	0/0	Progressive loading, resistance or strength practice predominates; not a mixed CrossFit WOD.
Kortas et al., 2020	Nordic walking	Aerobic/concurrent	1/0	Nordic walking is endurance-oriented activity.
Zhou et al., 2021	Strength or balance training	Resistance-based (strength); Other targeted exercise (balance)	0/0	Active arms are separately described; no non-exercise primary contrast.
Guo et al., 2025	Individualized resistance training	Resistance-based	1/0	Progressive loading, resistance or strength practice predominates; not a mixed CrossFit WOD.
Brooks et al., 2010	Resistance exercise during bed rest	Resistance-based	0/0	Progressive loading, resistance or strength practice predominates; not a mixed CrossFit WOD.
Dawson et al., 2021	Periodized resistance training	Resistance-based	0/0	Progressive loading, resistance or strength practice predominates; not a mixed CrossFit WOD.
Ghayomzadeh et al., 2022	Concurrent training	Aerobic/concurrent	1/0	Concurrent aerobic and resistance training.
Gould et al., 2025	Aerobic or concurrent training	Aerobic/concurrent	0/0	Both aerobic and concurrent arms fit this broad category; reported biopsy responses are not primary circulating inputs.
Zuo et al., 2023	Functional or traditional resistance training	Resistance-based	0/0	Progressive loading, resistance or strength practice predominates; not a mixed CrossFit WOD.
Watson et al., 2017	Progressive resistance training	Resistance-based	0/0	Progressive loading, resistance or strength practice predominates; not a mixed CrossFit WOD.
Radziminska et al., 2018	Pelvic-floor muscle training	Other targeted exercise	0/0	Isolated pelvic-floor training, not a whole-body resistance/aerobic/HIFT moderator input.

Study	Reported training	Operational class	Primary effects MSTN/FST	Basis
Razi et al., 2026	HIFT/CrossFit	HIFT/CrossFit	2/2	Explicit CrossFit/HIFT programme with mixed strength/endurance WODs; classification takes precedence over the general concurrent category.
Turon-Skrzypinska et al., 2026	Intradialytic virtual-reality exercise	Aerobic/concurrent	1/0	Intradialytic VR uses a rehabilitation rotor/cycling, not a primarily strength-set prescription.
Brown et al., 2024	Aerobic exercise	Aerobic/concurrent	0/0	Aerobic training.
Benavente et al., 2024	Whole-body resistance training	Resistance-based	0/0	Progressive loading, resistance or strength practice predominates; not a mixed CrossFit WOD.
Pazokian et al., 2022	Functional training with or without BFR	Resistance-based	2/2	Functional exercise with or without BFR is resistance-based; the word functional alone does not establish mixed HIFT/CrossFit.
Jang et al., 2016	Open- and closed-chain pulley exercises	Resistance-based	0/1	Progressive loading, resistance or strength practice predominates; not a mixed CrossFit WOD.
Arrieta, H. (2019)	Twice-weekly multicomponent strength, balance and gait training	Other multicomponent	2/0	Two nonoverlapping sex strata clustered within one trial; exact weeks not inferred
Motahari Rad, M (2023)	Concurrent aerobic/resistance in opposite sequences; 3/week	Aerobic/concurrent	2/2	Graphical SD; analyzed rather than randomized *n* used
Bagheri, R. (2019)	Upper-, lower-, or combined-body RT; 3/week	Resistance-based	3/3	Three contrasts share one control; Figure 2 states SD
Bagheri, R (2020)	Endurance then resistance or reverse order; 3/week	No primary moderator assignment	0/0	Supplied pre-proof has Figure 2 caption but no actual target graph or extractable control dispersion; narrative only
Diel, P. (2010)	Resistance or endurance; 3/week	Aerobic/concurrent; Resistance-based	0/2	MYOPRO is propeptide, not circulating mature MSTN; human SD convention differs from animal SEM
Mafi, F (2019)	RT with or without epicatechin; 3/week	Resistance-based	2/2	Change-score SD; RT vs placebo and RT+EP vs EP only; specimen wording conflict retained
Ghobadi, H. (2024)	Autoregulated versus linear periodized resistance exercise; 3/week	No primary moderator assignment	0/0	Narrative direct-training comparison; no nonexercise arm
Willoughby, D.S. (2004)	Heavy resistance training; 3/week	Resistance-based	1/0	Serum and muscle levels separated; noncommercial assay sensitivity retained
Koczulla, A.R. (2020)	Whole-body vibration at therapeutic frequency	No primary moderator assignment	0/0	Direct vibration-dose contrast; not pooled as inactive control
Bagheri, R. (2021)	RT+placebo or RT+BCAA; 3/week	Resistance-based	1/1	RT+placebo comparison retained with uncertainty in banana/dinner matching across groups; exclusion sensitivity reported; Figure 2 SEM converted to SD
Saremi, A. (2010)	RT+placebo or RT+creatine; 3/week	Resistance-based	1/0	Only RT+placebo vs control; creatine arm not isolated
Lenk, K. (2012)	Supervised exercise training	No primary moderator assignment	0/0	Serum numerical data explicitly not shown; secondary muscle outcomes only
Shabkhiz, F (2021)	Resistance training; 3/week	Resistance-based	2/0	Two nonoverlapping health strata remain one study cluster
Schiffer, T (2011)	Strength or moderate endurance training	No primary moderator assignment	0/0	No clear chronic group differences; not pooled with blood concentrations

Study	Reported training	Operational class	Primary effects MSTN/FST	Basis
Heisterberg, M.F. (2018)	Heavy RT plus losartan or placebo	No primary moderator assignment	0/0	Use losartan+RT versus losartan-only to isolate exercise; placebo+RT is not a matched drug comparison
Micielska, K. (2019)	High-intensity circuit training; 15 sessions	No primary moderator assignment	0/0	Not synthesized quantitatively because the target graph did not clearly identify the error-bar definition.
Nilsen TS (2016)	Strength training	No primary moderator assignment	0/0	Secondary outcome; not circulating concentration
Hulmi, J.J. (2009)	RT twice weekly with protein or placebo	No primary moderator assignment	0/0	Chronic resting and acute biopsy contrasts separated; linked to Hulmi companion report
Lewis, M.I. (2007)	Resistance training with or without testosterone	No primary moderator assignment	0/0	Hormone cointervention matched; do not mix unmatched testosterone effects
Boeselt, T. (2017)	Individualized high-intensity aerobic/strength training twice weekly	Aerobic/concurrent	1/0	Use T1; source T2 table conflicts with explicit control follow-up of only 3 months; no 6-month contrast
Santos, A.R. (2015)	Strength versus power resistance-training regimens; 3/week	No primary moderator assignment	0/0	Narrative tissue outcome; RoB 2 assessment targets randomized power-versus-strength comparison, not the separately recruited control contrast
Lundberg, T.R. (2013)	Unilateral aerobic plus resistance exercise	No primary moderator assignment	0/0	Paired active comparison; no independent-groups SMD
Laurentino, G.C. (2012)	Low-load RT with blood-flow restriction or high-load RT	No primary moderator assignment	0/0	Active training contrasts and tissue expression only
Walker, K.S. (2004)	Whole-body resistance training	No primary moderator assignment	0/0	Active comparator and relative immunoreactivity; narrative only
Irimia, J.M. (2017)	Flywheel resistance exercise during bed rest	No primary moderator assignment	0/0	Secondary tissue outcome; no conversion to circulating MSTN
Mendez-Gutierrez A (2023)	Moderate or vigorous concurrent exercise; 3/week	Aerobic/concurrent	0/2	Figure 3J outcome-specific *n* and SE; chronic MSTN detected in fewer than half and not quantitatively reported
Bozyilan, E. (2026)	Added HIIT; usual kickboxing retained in both groups	No primary moderator assignment	0/0	Active training comparator; narrative only
Strojek, K. (2021)	Supervised pelvic-floor muscle training twice weekly	No primary moderator assignment	0/0	Median/IQR presentation and baseline imbalance; no unplanned conversion to means/SD
Farzanegi, P. (2021)	Upper-, lower-, or combined-body resistance training; 3/week	Resistance-based	3/3	Target post means/SD directly reported in Results; shared control retained
Barati, N. (2025)	Linear versus weekly undulating periodized RT	No primary moderator assignment	0/0	Narrative active-training comparison
Shabani, Ramin (2018)	Resistance, aerobic or combined training; 3/week, 65 min/session	Aerobic/concurrent; Resistance-based	3/0	One study contributes both resistance and aerobic/concurrent categories; subgroup study counts are not additive
Karimifard, M. (2023)	Rest-pause or traditional resistance training; 3/week	No primary moderator assignment	0/0	Not synthesized quantitatively because the graph uncertainty type and final control denominator were insufficiently reported.
Bijeh et al., 2022	RT with/without soy milk; 3 sessions/week	Resistance-based	0/0	Undefined target-graph error bars; narrative only

Study	Reported training	Operational class	Primary effects MSTN/FST	Basis
Riahy et al., 2024	HIIT or MICT; 3 sessions/week	Aerobic/concurrent	2/0	Two primary effects; analysis *n* 13/12/12
Niazi et al., 2025	Circuit RT at 40/60/80% 1RM; 3 sessions/week	Resistance-based	3/3	Six primary effects; printed means and graph-derived SD
Nasiri et al., 2022	Progressive Pilates; 3 sessions/week	Other structured exercise	1/1	Two primary effects; 11/11 analyzed; vector-graph mean/SD
Watson et al., 2022	Aerobic versus combined aerobic/RT	Aerobic/concurrent	0/0	Author-manuscript conversion; companion to Gould/ExTra CKD family; narrative only
Sönmez et al., 2026	HIIT versus moderate continuous training	Aerobic/concurrent	0/0	Active-comparator narrative only; no exercise versus nonexercise primary effect
de Souza et al., 2014	Strength, interval or concurrent training	Mixed eligible arms	0/0	Muscle-level narrative; distinct baseline/training history from Santos
Izaddoust et al., 2017	Resistance training	Resistance-based	0/0	Companion/subset report of Shabani et al., 2018; no additional independent primary effect. Family-level RoB 2 assessment is reported under Shabani et al., 2018 in Table S8A.
Rasi et al., 2023	Aerobic; 3/week, 45 min	Aerobic/concurrent	1/0	One direct mean/SD primary effect
Hooshmandi et al., 2022	High-intensity interval resistance training	Resistance-based	0/0	Ratios only; no reconstruction of absolute MSTN
Jazi et al., 2024	Elastic-band resistance training; 3/week	Resistance-based	1/0	One direct mean/SD effect
Jaafari et al., 2024	Combined training with or without BFR	Aerobic/concurrent	0/2	Two FST effects; MSTN not synthesized because the denominator was unresolved; duration excluded from dose regression.
Marvast et al., 2024	Progressive RT; 2–4/week	Resistance-based	1/1	Two direct mean/SD effects; source sampling contradiction retained; numerical error convention reviewed
Ramezani et al., 2022	Low/moderate/high interval intensity; 3/week	Aerobic/concurrent	3/0	Three effects share one control and one study cluster

Supplementary Table S11. Registry provenance and bounded linkage audit

Audit item	Finding
Direct ClinicalTrials.gov export	103 records; 102 routed to the registry queue; one consolidated with a publication record.
Additional queue sources	69 records through CENTRAL/Embase; 171 queue records in total, mapping to 162 stable registry identifiers.
Current ClinicalTrials.gov records retrieved	121 current NCT snapshots in the audit: all 115 NCT identifiers in the 162-identifier queue plus six additional included-study NCT identifiers outside that queue. The other 47 queue identifiers were non-NCT; snapshot retrieval does not establish historical outcome concordance.
Exact linkage to included reports	Two identifiers in the queue matched included studies (Hofmann and Rezaei).
Other candidate reports	45 identifiers with report/reference candidates; eligibility and study equivalence not adjudicated.
No link identified	72 identifiers with no result-report link in the bounded check; this does not mean unpublished.
Other unresolved identifiers	43 queue identifiers had unresolved publication status; 72 had no result-report link identified, 45 had other unadjudicated report/reference candidates, and two matched included reports by exact identifier. These four categories sum to 162; no category is an estimate of unpublished trials.
Included-study registration coverage	15 of the original 33 studies reported registration identifiers. Zuo is displayed once as ChiCTR2200059775, matching the publisher abstract and PubMed. Historical source strings remain in the audit and are not counted as separate verified registrations.
Current outcome lists	Seven included-study NCT records retrieved. One lists muscle myostatin expression; six lack explicit target entries. Neither finding establishes prospective concordance or outcome switching.
Unpublished trials / prospective concordance	Not estimable from this audit. No claim that registry linkage substitutes for small-study-effect testing.

The historical 171-record registry-linkage queue remains separate from the original 138-report retrieval queue. Renewed retrieval supplied 121 original report records; 17 remained not retrieved. Unavailable originals were not reclassified as registrations.

Supplementary File S1. Full electronic search strategies

The following search strategies reproduce the database-specific syntax recorded in the frozen search log and used to generate the exported record sets. Subject headings, field tags and platform settings are reported for reproducibility. No date or language limits were applied unless explicitly stated. Search-sensitivity limitation. The original bibliographic strategies omitted the standalone abbreviation MSTN and truncation, and the PubMed strategy omitted the Exercise MeSH heading and the standalone keyword exercise. These omissions may have reduced retrieval sensitivity. During revision, an expanded PubMed query was executed and exported, but its additional records were not eligibility-assessed or incorporated into an updated synthesis. This check does not establish search completeness or the absence of an effect on the findings. The historical strategies and search dates below remain those used for the evidence assessed in this review.

Web of Science Core Collection

TS=((myostatin OR "growth differentiation factor 8" OR "GDF-8" OR GDF8 OR follistatin) AND ("exercise training" OR "physical training" OR "training program" OR "training programme" OR "aerobic training" OR "endurance training" OR "resistance training" OR "strength training" OR "weight training" OR "concurrent training" OR "combined training" OR "circuit training" OR "interval training" OR HIIT OR CrossFit OR "whole-body vibration" OR "whole body vibration" OR "exercise intervention" OR "exercise program" OR "exercise programme"))

Scopus

TITLE-ABS-KEY((myostatin OR "growth differentiation factor 8" OR "GDF-8" OR GDF8 OR follistatin) AND ("exercise training" OR "physical training" OR "training program" OR "training programme" OR "aerobic training" OR "endurance training" OR "resistance training" OR "strength training" OR "weight training" OR "concurrent training" OR "combined training" OR "circuit training" OR "interval training" OR HIIT OR CrossFit OR "whole-body vibration" OR "whole body vibration" OR "exercise intervention" OR "exercise program" OR "exercise programme"))

Embase.com

('myostatin'/exp OR 'follistatin'/exp OR myostatin:ti,ab,kw OR 'growth differentiation factor 8':ti,ab,kw OR 'gdf 8':ti,ab,kw OR gdf8:ti,ab,kw OR follistatin:ti,ab,kw) AND ('exercise training'/exp OR 'resistance training'/exp OR 'aerobic exercise'/exp OR 'high intensity interval training'/exp OR 'exercise therapy'/exp OR 'exercise training':ti,ab,kw OR 'physical training':ti,ab,kw OR 'training program':ti,ab,kw OR 'training programme':ti,ab,kw OR 'aerobic training':ti,ab,kw OR 'endurance training':ti,ab,kw OR 'resistance training':ti,ab,kw OR 'strength training':ti,ab,kw OR 'weight training':ti,ab,kw OR 'concurrent training':ti,ab,kw OR 'combined training':ti,ab,kw OR 'circuit training':ti,ab,kw OR 'interval training':ti,ab,kw OR hiit:ti,ab,kw OR crossfit:ti,ab,kw OR 'whole body vibration':ti,ab,kw OR 'exercise intervention':ti,ab,kw OR 'exercise program':ti,ab,kw OR 'exercise programme':ti,ab,kw) NOT ('animal'/exp NOT 'human'/exp)

SPORTDiscus with Full Text, EBSCOhost

((TI (myostatin OR "growth differentiation factor 8" OR "GDF-8" OR GDF8 OR follistatin)) OR (AB (myostatin OR "growth differentiation factor 8" OR "GDF-8" OR GDF8 OR follistatin)) OR (SU (myostatin OR "growth differentiation factor 8" OR "GDF-8" OR GDF8 OR follistatin))) AND ((TI ("exercise training" OR "physical training" OR "training program" OR "training programme" OR "aerobic training" OR "endurance training" OR "resistance training" OR "strength training" OR "weight training" OR "concurrent training" OR "combined training" OR "circuit training" OR "interval training" OR HIIT OR CrossFit OR "whole-body vibration" OR "whole body vibration" OR "exercise intervention" OR "exercise program" OR "exercise programme")) OR (AB ("exercise training" OR "physical training" OR "training program" OR "training programme" OR "aerobic training" OR "endurance training" OR "resistance training" OR "strength training" OR "weight training" OR "concurrent training" OR "combined training" OR "circuit training" OR "interval training" OR HIIT OR CrossFit OR "whole-body vibration" OR "whole body vibration" OR "exercise intervention" OR "exercise program" OR "exercise programme")) OR (SU ("exercise training" OR "physical training" OR "training program" OR "training programme" OR "aerobic training" OR "endurance training" OR "resistance training" OR "strength training" OR "weight training" OR "concurrent training" OR "combined training" OR "circuit training" OR "interval training" OR HIIT OR CrossFit OR "whole-body vibration" OR "whole body vibration" OR "exercise intervention" OR "exercise program" OR "exercise programme")))

Cochrane CENTRAL

(myostatin OR "growth differentiation factor 8" OR "GDF-8" OR GDF8 OR follistatin) AND ("exercise training" OR "physical training" OR "training program" OR "training programme" OR "aerobic training" OR "endurance training" OR "resistance training" OR "strength training" OR "weight training" OR "concurrent training" OR "combined training" OR "circuit training" OR "interval training" OR HIIT OR CrossFit OR "whole-body vibration" OR "whole body vibration" OR "exercise intervention" OR "exercise program" OR "exercise programme")

PubMed

(myostatin[Title/Abstract] OR "growth differentiation factor 8"[Title/Abstract] OR "GDF-8"[Title/Abstract] OR GDF8[Title/Abstract] OR follistatin[Title/Abstract]) AND ("Resistance Training"[MeSH Terms] OR "Exercise Therapy"[MeSH Terms] OR "Physical Conditioning, Human"[MeSH Terms] OR "exercise training"[Title/Abstract] OR "physical training"[Title/Abstract] OR "training program"[Title/Abstract] OR "training programme"[Title/Abstract] OR "aerobic training"[Title/Abstract] OR "endurance training"[Title/Abstract] OR "resistance training"[Title/Abstract] OR "strength training"[Title/Abstract] OR "weight training"[Title/Abstract] OR "concurrent training"[Title/Abstract] OR "combined training"[Title/Abstract] OR "circuit training"[Title/Abstract] OR "interval training"[Title/Abstract] OR HIIT[Title/Abstract] OR CrossFit[Title/Abstract] OR "whole-body vibration"[Title/Abstract] OR "whole body vibration"[Title/Abstract] OR "exercise intervention"[Title/Abstract] OR "exercise program"[Title/Abstract] OR "exercise programme"[Title/Abstract])

MEDLINE Ultimate, EBSCOhost

((MH "Myostatin+") OR (MH "Follistatin+") OR TI (myostatin OR "growth differentiation factor 8" OR "GDF-8" OR GDF8 OR follistatin) OR AB (myostatin OR "growth differentiation factor 8" OR "GDF-8" OR GDF8 OR follistatin)) AND ((MH "Resistance Training+") OR (MH "Exercise Therapy+") OR (MH "Physical Conditioning, Human+") OR TI ("exercise training" OR "physical training" OR "training program" OR "training programme" OR "aerobic training" OR "endurance training" OR "resistance training" OR "strength training" OR "weight training" OR "concurrent training" OR "combined training" OR "circuit training" OR "interval training" OR HIIT OR CrossFit OR "whole-body vibration" OR "whole body vibration" OR "exercise intervention" OR "exercise program" OR "exercise programme") OR AB ("exercise training" OR "physical training" OR "training program" OR "training programme" OR "aerobic training" OR "endurance training" OR "resistance training" OR "strength training" OR "weight training" OR "concurrent training" OR "combined training" OR "circuit training" OR "interval training" OR HIIT OR CrossFit OR "whole-body vibration" OR "whole body vibration" OR "exercise intervention" OR "exercise program" OR "exercise programme"))

ClinicalTrials.gov Expert Search

AREA[BasicSearch](EXPANSION[None](myostatin OR "growth differentiation factor 8" OR "GDF-8" OR GDF8 OR follistatin OR MSTN)) AND AREA[BasicSearch](exercise OR training OR "physical activity" OR "exercise training" OR "physical training" OR "training program" OR "training programme" OR "aerobic training" OR "endurance training" OR "resistance training" OR "strength training" OR "weight training" OR "concurrent training" OR "combined training" OR "circuit training" OR "interval training" OR HIIT OR CrossFit OR "whole-body vibration" OR "whole body vibration" OR "exercise intervention" OR "exercise program" OR "exercise programme")
